# Stereo-Controlled Liquid Phase Synthesis of Phosphorothioate Oligonucleotides on a Soluble Support

**DOI:** 10.1021/acs.joc.3c01006

**Published:** 2023-07-10

**Authors:** Petja Rosenqvist, Verneri Saari, Ella Pajuniemi, Alejandro Gimenez Molina, Mikko Ora, Andras Horvath, Pasi Virta

**Affiliations:** † Department of Chemistry, University of Turku, 20500 Turku, Finland; ‡ Chemical Process Research & Development, Janssen Pharmaceutical Companies of Johnson & Johnson, 2340 Beerse, Belgium

## Abstract

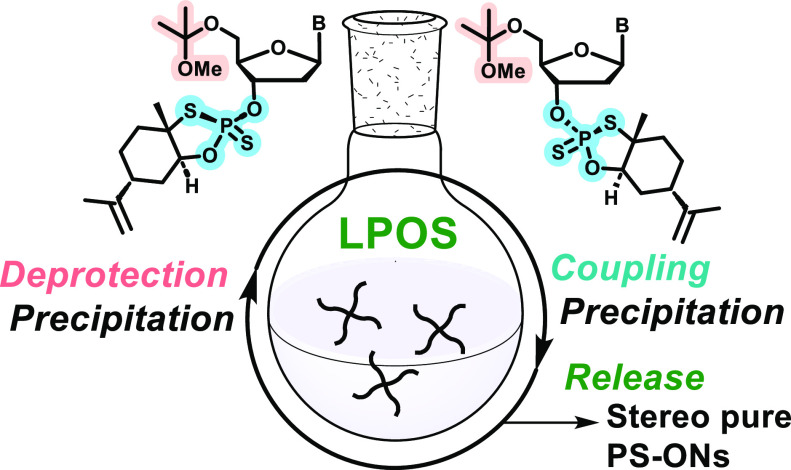

5′-*O*-(2-Methoxyisopropyl) (MIP)-protected 2′-deoxynucleosides as chiral P(V)-building blocks, based on the limonene-derived oxathiaphospholane sulfide, were synthesized and used for the assembly of di-, tri-, and tetranucleotide phosphorothioates on a tetrapodal pentaerythritol-derived soluble support. The synthesis cycle consisted of two reactions and two precipitations: (1) the coupling under basic conditions, followed by neutralization and precipitation and (2) an acid catalyzed 5′-*O*-deacetalization, followed by neutralization and precipitation. The simple P(V) chemistry together with the facile 5′-*O*-MIP deprotection proved efficient in the liquid phase oligonucleotide synthesis (LPOS). Ammonolysis released nearly homogeneous Rp or Sp phosphorothioate diastereomers in ca. 80% yield/synthesis cycle.

## Introduction

The importance of therapeutic oligonucleotides (ONs) for treating human diseases is exponentially increasing.^1^ ON-based treatments have progressed beyond niche applications into areas of therapeutic targets, including hepatitis B and cardiovascular diseases, which require significantly increased amounts of ONs due to the large number of potential patients.^2^ The growing need for ONs has challenged current ON manufacturing that relies on the automated solid phase ON synthesis (SPOS).^3^ SPOS has many operational benefits, but its suitability for real-time optimized large-scale processing is limited. This and the recognized sustainability issues are prompting to develop alternative synthetic strategies aiming to greener reagents, improved reagent efficiency, better scalability, and minimized waste streams.^4^ In this context, the liquid phase-occurring technologies, based on soluble supports, are under investigation, which facilitate isolation of the growing ON intermediates by membrane filtration or precipitation and allow ON chain elongation in real time-optimized and process–suitable reaction conditions.^5−7^

**Scheme 1:**
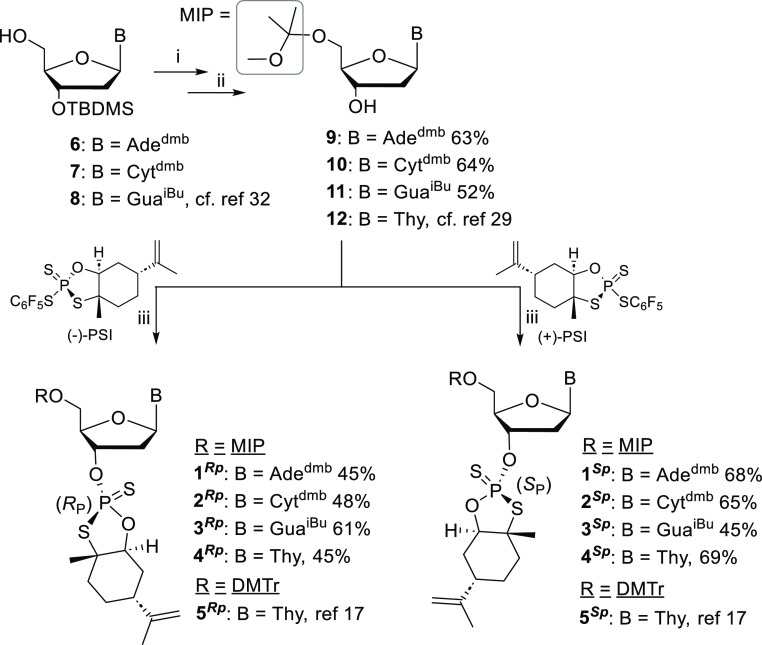
Synthesis of 3′-*O*-Ψ-Loaded 5′-*O*-MIP-2′-deoxynucleosides Conditions: (i) 2,2-dimethoxypropane, THF, TsOH·H_2_O, at r.t. for 3 h; (ii) TBAF·3H_2_O, THF, at r.t. for 2 h—overnight; (iii) (−)-PSI/(+)-PSI, DBU, MeCN. at r.t. for 2 h.

An additional sustainability challenge of ONs is the growing interest toward enantiopure phosphorothioates.^8−15^ The proper Rp/Sp-design may not only lead to enhanced efficacy,^16^ reduced toxicity, and improved delivery of ONs but also to a substantial investment considering the control and regulation of stereochemical integrity and screening of the potential ON drug candidates. Recently, Baran introduced a limonene-based P(V)-chemistry,^17,18^ an extension of the work by Stec,^19−22^ which will likely have a significant impact in preparation of enantiopure phosphorothioate ONs. Furthermore, the simple and fast redox-neutral coupling chemistry, based on reactivity of the oxathiaphospholane sulfide moiety (Scheme 1), may suit well for the liquid phase oligonucleotide synthesis (LPOS).^23^

**Scheme 2:**
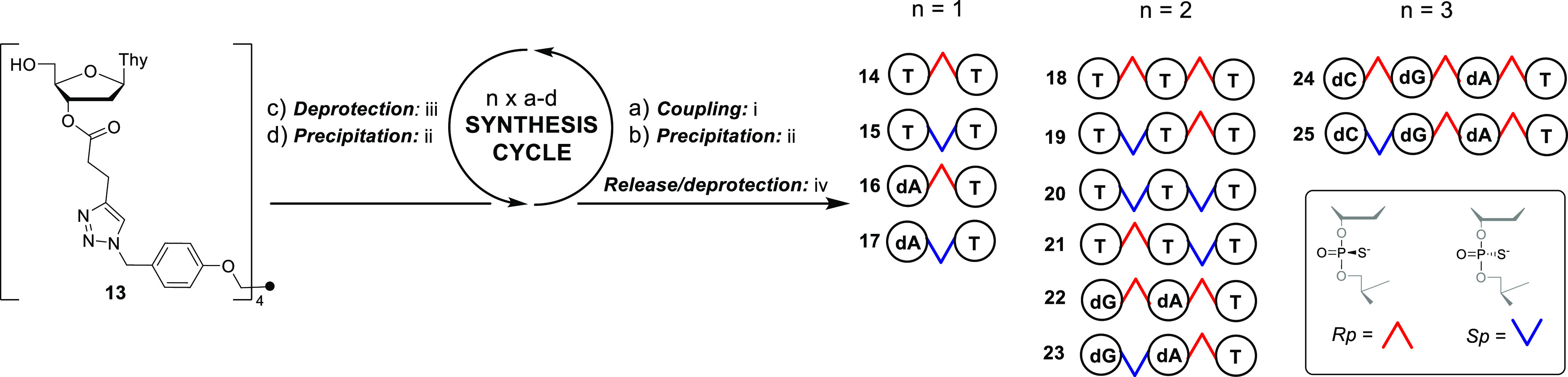
Stereo-Controlled LPOS of Di-, Tri-, and Tetranucleotide Phosphorothioates Conditions: (i) 1.7 equiv **1–4**^**Rp/Sp**^, 2.7 equiv DBU (equiv/5′-OH group), Py/MeCN (2:3, v/v), for 15–30 min at r.t., followed by addition of acetic acid (3 equiv); (ii) product mixture precipitated in 2-propanol; (iii) 5% DCA in MeOH/DCM, for 3–6 min at r.t., followed by addition of pyridine (2 equiv in comparison to DCA); (iv) 25% aqueous ammonia, for 5 h at 55 °C.

In the present study, 5′-*O*-(2-methoxyisopropyl) (MIP)-protected 2′-deoxynucleosides as chiral limonene-based oxathiaphospholane sulfide [named as (+) and (−)-Ψ] building blocks (**1–4**^**Rp/Sp**^, Scheme 1) were synthesized and their applicability for the stereo-controlled LPOS of di-, tri-, and tetranucleotide phosphorothioates using a tetrapodal precipitative soluble support was demonstrated (Scheme 2). This is a preliminary study, which will guide to find procedures for stereo-controlled LPOS of longer ON sequences, but especially of short ON fragments^24^ or blockmers^25,26^ that can be ligated to gain full length ON products.

## Results and Discussion

### 3′-*O*-Ψ-Loaded 5′-*O*-MIP-2′-deoxynucleosides

4,4′-Dimethoxytrityl (DMTr) is an established 5′-*O*-protecting group in SPOS^27^ but causes issues in LPOS.^4^ The reversibility of the deprotection (Sn1) and its hard reproducibility, being dependent on the scale, concentration, ON sequence and its length, may lead to a marked depurination in solution (primarily in case of DNA). Despite the promising examples, in which scavengers (e.g., silanes^28^ and thiols^24^) are used in the detritylation cocktail, we favored MIP as an alternative 5′-*O*-protecting group.^29^ The pseudo irreversible acid-catalyzed removal of MIP yields volatile byproducts: acetone and methanol. This, together with a faster reaction rate,^30^ leads to cleaner products and reduced depurination, which is a clear improvement in comparison to DMTr when used in solution. Synthesis of 3′-*O*-Ψ-loaded 5′-*O*-MIP-2′-deoxynucleosides (**1**^**Rp**^**–4**^**Rp**^ and **1**^**Sp**^**–4**^**Sp**^) is described in Scheme 1. Amidine-protected nucleobases have recently been used with the limonene-based P(V)-chemistry in SPOS.^18^ In our preliminary trials, issues related to premature cleavage of benzoyl at adenine and cytosine were noticed. To ensure better stability, 2,4-dimethylbenzoyl (dmb, ca. 20-fold more stable to alcoholysis than Bz^31^) was used for 2′-deoxyadosine and 2′-deoxycytidine (dA^dmb^ and dC^dmb^). Standard isobutyryl was used for 2′-deoxyguanosine (dG^iBu^), and no N3-protection was needed for thymidine. Dmb protection was performed using a similar protocol than used typically for the benzoylation. MIP introduction was done using the 3′-*O*-*tert*-butyldimethlysilyl (TBDMS)-protected 2′-deoxynucleosides (**6–8**^32^) as subjects for acetalization in a 1:1-mixture of dimethoxypropane and THF in the presence of a catalytic amount of TsOH·H_2_O (preparation of **6** and **7** described in the Supporting Information). After an extractive work up (or a flash chromatography in case of **9**), the 3′-*O*-TBDMS group was removed, which gave the desired 5′-*O*-MIP-dA^dmb^ (**9**), dC^dmb^ (**10**), and dG^iBu^ (**11**) in 87, 64, and 52% isolated yield (over two steps), respectively. 5′-*O*-MIP-T (**12**) has been published previously.^29^ The 5′-*O*-MIP protected 2′deoxynucleosides (**9–12**) were treated with commercially available (−)- and (+)-Ψ-reagents (1.3 equiv) in the presence of stoichiometric amount of 1,8-diazabicyclo[5.4.0]undec-7-ene (DBU) (1.3 equiv), which gave the desired Ψ-loaded products (**1**^**Rp**^**–4**^**Rp**^ and **1**^**Sp**^**–4**^**Sp**^) in 45–69% isolated yields. Due to the modest selectivity between the 5′-*O*- and 3′-*O*-acetalization, a complex synthesis route for the building blocks (**1**^**Rp**^**–4**^**Rp**^ and **1**^**Sp**^**–4**^**Sp**^) is described. Work on the optimized direct selective 5′-*O*-MIP protection is under way, which will reduce the price and carbon footprint of the 5′-*O*-MIP building blocks, making them real competitive alternatives for DMTr-protected ones.

### Stereocontrolled LPOS Using 3′-*O*-Ψ-Loaded 2′-deoxynucleosides

**Figure 1:**
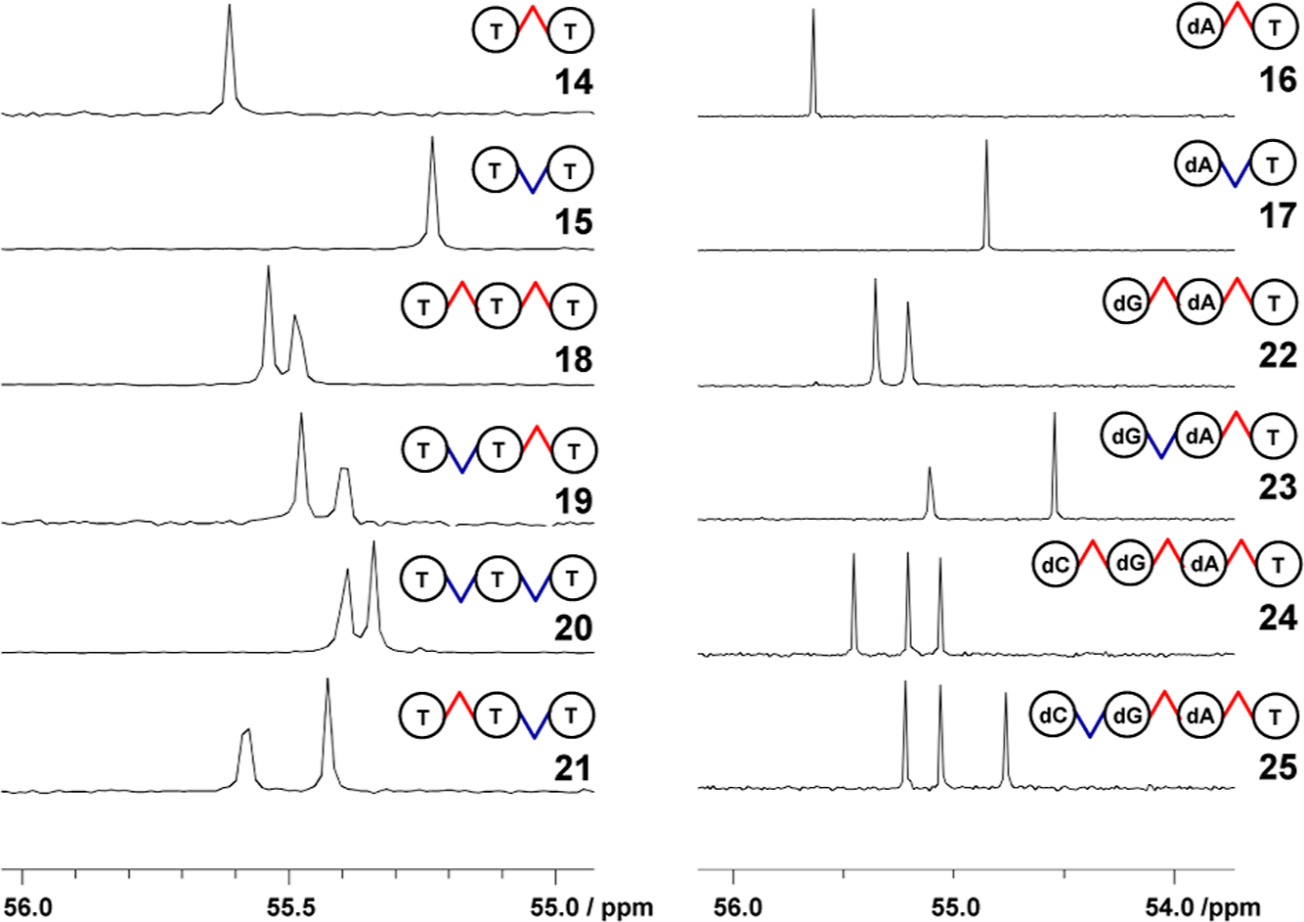
^31^P NMR (200 MHz, D_2_O) spectra **14–25**. cf. conditions in General Methods.

We have previously used a pentaerytritol-derived branching unit as a soluble support for LPOS.^33−37^ The protected tetrapodal nucleotide constructs assembled on this unit can be isolated from the reaction media by precipitation in protic antisolvents. The same branching unit, loaded by thymidine and an ester linker is used in the present study (**13**, Scheme 2). First, we evaluated the compatibility of 5′-*O*-MIP vs 5′-*O*-DMTr-protected 3′-*O*-Ψ-loaded thymidine building blocks (**4**^**Rp**^, **4**^**Sp**^ vs **5**^**Rp**^, and **5**^**Sp**^(17)) for the assembly of dithymidine phosphorothioates **14** and **15** (Scheme 2). The efficiency of the coupling and 5′-deprotection was monitored by RP HPLC (a and c/synthesis cycle, Scheme 2. A mixture of pyridine/MeCN (2:3, v/v) was used as a solvent system for the coupling. The building blocks (**1–5**^**Rp/Sp**^) and the tetrapodal nucleotide constructs were readily soluble into this system, and it consisted of recoverable low boiling point volatiles and was tolerated well in the following precipitation step (b/synthesis cycle). Quantitative coupling was obtained in 15 min by using building blocks **4**^**Rp/Sp**^ or **5**^**Rp/Sp**^ (1.7 equiv/5′-OH group, 0.17 mol L^–1^) in the presence of an excess of DBU (2.7 equiv/5′-OH group, 0.27 mol L^–1^). No marked change in the coupling efficiency was observed, whether 5′-*O*-DMTr or MIP-protected building blocks were used (Figure S59). After the coupling, the reaction mixtures were neutralized by addition acetic acid (3 equiv used to neutralize DBU, not pyridine) and precipitated in 2-propanol, being an optimal antisolvent (2-propanol, MeOH and Et_2_O tested) to provide near quantitative recovery of the tetrapodal nucleotide constructs and efficient removal of the contaminants. For the removal of the 5′-*O*-DMTr and MIP, the precipitates were dissolved in a mixture of dichloroacetic acid (DCA) (5% for MIP and 20% for DMTr removal) in dichloromethane (DCM)/MeOH (2:1, v/v). Traces of tritylated products could be observed even after a prolonged DCA treatment (2 h, no scavengers used), whereas complete MIP removal was achieved in 3–6 min (Figure S60). The deprotection mixtures were neutralized by addition of pyridine (2 equiv in comparison to DCA) and precipitated in 2-propanol (d/synthesis cycle). The residues were exposed to aqueous ammonia that released dithymidine phosphorothioates **14** and **15** in average 77% yield (calculated from **13** and based on UV-absorbance of aqueous solutions of the products at λ = 260 nm). ^31^P NMR confirmed the stereochemical purity of the products (Figure 1).

**Figure 2:**
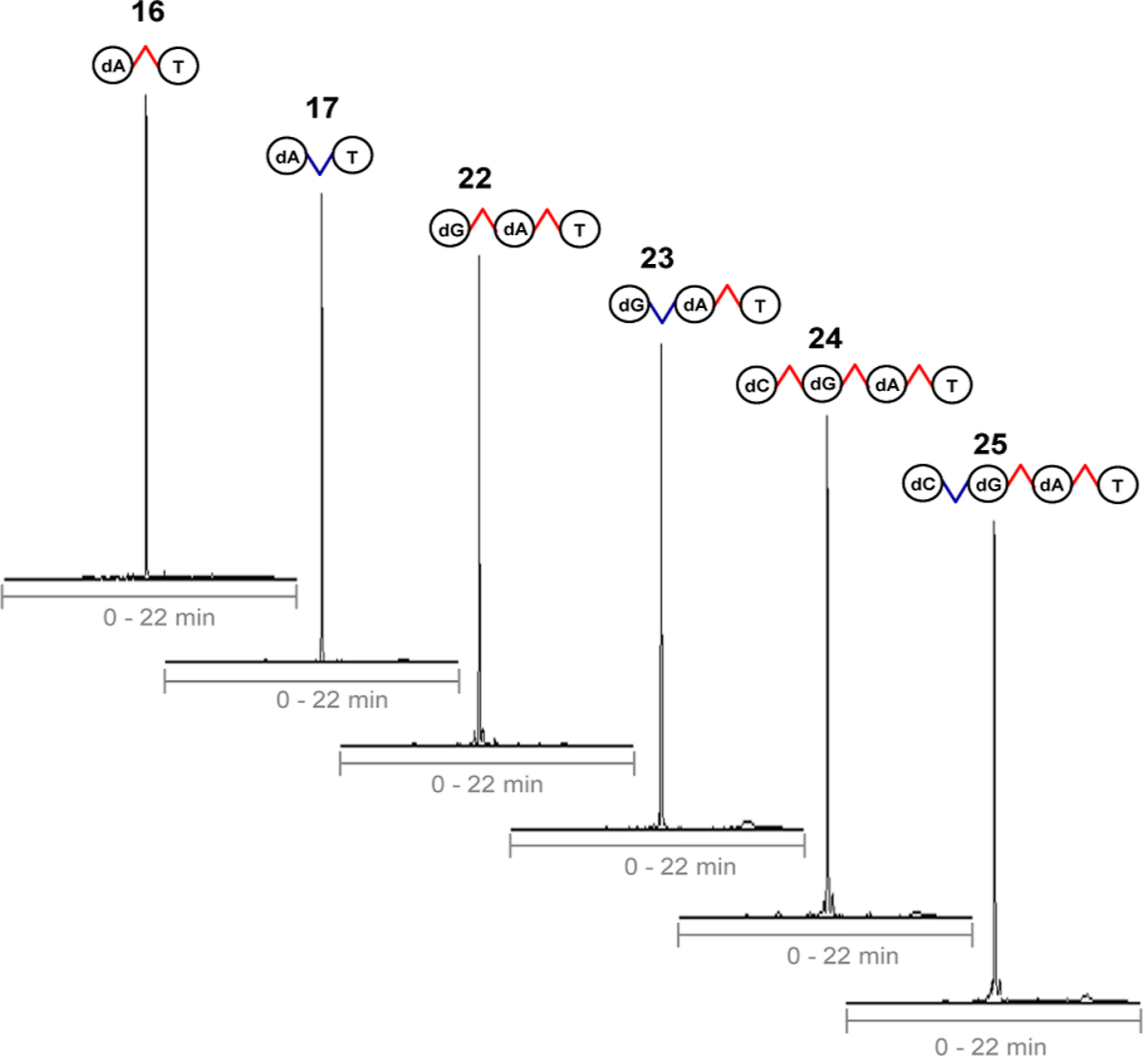
Examples of RP HPLC profiles of crude product (**16**, **17**, **22–24**) mixtures. cf. conditions in General Methods.

Encouraged by the successful MIP-Ψ-combination in LPOS, all stereoisomers of trithymidine phosphorothioates (**18–21**) and heteromeric di-, tri-, and tetranucleotide phosphorothioates (**16**, **17**, **22–25**) were then assembled using **1–4**^**Rp/Sp**^. As above, RP HPLC was used to monitor each coupling and deprotection. A longer (30 min) coupling time was needed for the 2′-deoxycytidine and guanosine building blocks **2**^**Rp**^, **2**^**Sp**^, **3**^**Rp**^, and **3**^**Sp**^. Concentrated ammonia released di- (**16**, **17**), tri- (**22**, **23**), and tetranucleotide phosphorothioates (**24**, **25**) in 74–81, 56–66, and 53–59% yields (based on UV-absorbance of the released ONs at λ = 260 nm), respectively, referring to ca. 80% average yield/synthesis cycle. RP HPLC profiles of the crude product mixtures of heteromeric nucleotides are described in Figure 2 (Figure S61). As seen, the limonene-based P(V) chemistry resulted in nearly quantitative couplings that led to efficient chain elongation (>95% purity in each case). The determined overall yields remained lower than expected due to the precipitation efficiency of the soluble support constructs. In each product, ^31^P NMR was used to confirm the stereochemical purity of the phosphorothioate linkages (Figure 1). In general, ^31^P NMR works well for this purpose as distinct and resolved ^31^P resonance signals were observed. Evidence of the stereochemical integrity was provided by exposing the heteronucleotide products (**16**,**17**, **22–25**) to snake venom phosphodiesterase^38−42^ that selectively cleaved Rp-isomers of the phosphodiester linkages (Figures S62–S64).

## Conclusions

5′-*O*-(2-Methoxypropane-2-yl) (MIP)-protected 2′-deoxynucleosides, 3′-*O*-loaded by chiral limonene-based oxathiaphospholane sulfide (i.e. Ψ-moiety) (**1–4**^**Rp**^ and **1–4**^**Sp**^), were synthesized and used for stereo-controlled LPOS of di-, tri-, and tetranucleotides on a precipitative soluble support (**13**). The facile acid-catalyzed removal of MIP and redox-neutral coupling of the Ψ-building blocks proved a useful combination in LPOS. The target nucleotides were obtained in ca. 80% average yield/synthesis cycle that consisted of (1) coupling of the P(V) building blocks (1.7 equiv/5′-OH group) in the presence of DBU (2.7 equiv/5′-OH group), followed by neutralization (AcOH) and precipitation in 2-propanol, and (2) MIP-deprotection using 5% DCA in a mixture of MeOH/DCM, followed by neutralization (pyridine) and precipitation in 2-propanol. ^31^P NMR spectroscopy confirmed the stereo chemical purity of the products (**14–25**). In addition, the stereochemical Rp/Sp-integrity (of **16**, **17**, **22–25**) was verified by exposing the nucleotides to snake venom phosphodiesterase that selectively hydrolysed Rp-isomers of the phosphorothioate linkages. This LPOS-compatible procedure may find applications in a scalable preparation of stereopure phosphorothioate ONs. The further development of this methodology may evaluate how long ON sequences can be assembled maintaining still the sufficient efficiency and purity of the ON products. The precipitation efficiency and/or solubility of the tetrapodal ON constructs may need an adjusted nucleobase protecting group scheme.^43^ An orthogonal linker chemistry would allow preparation of protected blockmers^25,26^ or fragments,^24^ which with an appropriate ligation chemistry may be used for the assembly of full-length ON products. It may be emphasized that recent improvements in liquid phase utilize tetra- and pentameric fragments for the convergent assembly of ONs in a kg scale.^24^ Some benefits of LPOS may be highlighted in the stereo-controlled synthesis. The real time monitoring and controlling of stereochemical integrity of phosphorothioate ONs are limited in the monomer-based assembly on a solid phase. In addition, the end products are contaminated by accumulating diastereomeric byproducts, potentially formed in each coupling during chain elongation. The stereo-controlled LPOS offers better access to real time monitoring of the couplings. With an efficient ligation chemistry of the corresponding pre-characterized and homogenized ON blockmers or segments, assembly of high quality stereo pure ONs may be improved.

## Experimental Section

### General Methods

NMR spectra were recorded on Bruker Avance 500 and 600 MHz instruments. 31P-NMR spectra of phosphorothioate oligomers were recorded in a mixture of 0.2 M pH 7.4 sodium cacodylate buffer and D_2_O (4:1, v/v). Mass spectra were recorded on a Bruker microQTOF ESI mass spectrometer. DCM and DMF were dried over 4 Å molecular sieves and MeCN, and MeOH over 3 Å molecular sieves. DBU was dried over CaH_2_. The homogeneity of the phosphorothioates and the composition of the samples withdrawn from the reaction solutions were analyzed by RP HPLC (an analytical C18 column, 4.6 × 250 mm, 5 μm, flow rate 1 mL/min) using a mixture of 50 mM TEAA-buffer and MeCN. The samples from the reaction mixture were eluted by using linear gradient from 40 to 70% MeCN over 20 min. Signals were recorded on a UV detector at a wavelength of 260 nm.

#### *N*^4^-(2,4-Dimethylbenzoyl)-5′-*O*-(2-methoxypropane-2-yl)-2′-deoxyadenosine (**9**)

To a solution of **6** (67 g, 135 mmol) in THF (330 mL) and 2,2-dimethoxypropane (330 mL) at 0–5 °C TsOH·H_2_O (1.28 g, 6.73 mmol, 0.05 equiv) was added. The reaction mixture was allowed to warm up, then stirred at room temperature for 3 h, quenched by addition of triethylamine, and concentrated. The residue was eluted though a short silica gel column (heptane/ethyl acetate, 3:1–2:1, v/v). The product fractions were combined and evaporated to dryness to give 55 g (72%) of 3′-*O*-*tert*-butyldimethylsilyl-*N*^4^-(2,4-dimethylbenzoyl)-5′-*O*-(2-methoxypropane-2-yl)-2′-deoxyadenosine as white solid. The intermediate product (55 g, 97 mmol) was dissolved in THF (550 mL), and the mixture was cooled to 0–5 °C. TBAF·3H_2_O (31 g, 97 mmol) was added and the mixture was stirred at room temperature overnight. The reaction solution was concentrated and purified by silica gel column chromatography (*n*-heptane/ethyl acetate, 1:1, v/v) to give 39 g (63% from **6**) of the product (**9**) as white solid. ^1^H NMR (500 MHz, DMSO-*d*_6_): δ 11.01 (s, 1H), 8.71 (s, 1H), 8.65 (s, 1H), 7.50 (d, 1H, *J* = 7.7 Hz), 7.11 (s, 1H), 7.08 (d, 1H, *J* = 8.0 Hz), 6.52 (t, 1H, *J* = 6.5 Hz), 5.48 (d, 1H, *J* = 4.4 Hz), 4.52 (m, 1H), 4.08–3.97 (m, 1H), 3.59 (dd, 1H, *J* = 10.5, 4.0 Hz), 3.50 (dd, 1H, *J* = 10.5, 5.4 Hz), 3.00 (s, 3H), 2.91–2.83 (m, 1H, *J* = 12.9, 6.3 Hz), 2.48–2.39 (m, 4H), 2.32 (s, 3H), 1.25 (s, 6H); ^13^C{1H} NMR (126 MHz, DMSO-*d*_6_): δ 167.8, 151.9, 151.7, 150.1, 142.8, 140.2, 136.7, 132.5, 131.5, 128.4, 126.1, 125.3, 99.6, 86.0, 83.5, 70.8, 61.0, 47.8, 39.5, 24.2, 24.1, 20.9, 19.8. HRMS (ESI) *m*/*z*: [M – H]^−^ calcd for C_23_H_28_N_5_O_5_^–^, 454.2096; found, 454.2111.

#### *N*^4^-(2,4-Dimethylbenzoyl)-5′-*O*-(2-methoxypropane-2-yl)-2′-deoxycytidine (**10**)

To a solution of **7** (58.0 g, 123 mmol) in THF (290 mL) and 2,2-dimethoxypropane (290 mL), TsOH·H_2_O (2.33 g, 12.26 mmol, 0.1 equiv) was added. The reaction was stirred at 25 °C for 2 h and quenched by addition of NaHCO_3_ (21 g, 245 mmol, 2.0 equiv) and Et_3_N (58 mL). The mixture was stirred for 30 min and concentrated to dryness. The residue was dissolved in THF (290 mL) and TBAF·3H_2_O (64.0 g, 245 mmol, 2.0 equiv) was added. After stirring at 25 °C for 2 h, the mixture was concentrated to dryness. The residue was re-dissolved in DCM (580 mL) and washed with water. The organic phase was separated, dried over Na_2_SO_4_, filtered, and concentrated to dryness. The crude was purified by silica gel chromatography (EtOAc/DCM = 1:2–1:0, v/v) to give 34 g (64%) of the product (**10**) as white solid. ^1^H NMR (500 MHz, DMSO-*d*_6_): δ 11.08 (s, 1H), 8.34 (d, 1H, *J* = 7.5 Hz), 7.40 (d, 1H, *J* = 7.8 Hz), 7.35 (d, 1H, *J* = 7.4 Hz), 7.10 (s, 1H), 7.08 (d, 1H, *J* = 8.1 Hz), 6.14 (t, 1H, *J* = 6.0 Hz), 5.36 (d, 1H, *J* = 4.5 Hz), 4.31–4.17 (m, 1H), 3.98 (q, 1H, *J* = 3.9 Hz), 3.60 (dd, 1H, *J* = 10.8, 3.5 Hz), 3.53 (dd, 1H, *J* = 10.8, 4.1 Hz), 3.12 (s, 3H), 2.36 (s, 3H), 2.35–2.32 (m, 1H), 2.31 (s, 3H), 2.15–2.05 (m, 1H), 1.32 (s, 3H), 1.30 (s, 3H); ^13^C{1H} NMR (126 MHz, DMSO-*d*_6_): δ 169.6, 162.8, 154.4, 144.6, 140.5, 136.4, 132.1, 131.4, 128.3, 126.1, 99.8, 95.6, 86.2, 86.0, 69.9, 60.2, 48.1, 40.9, 24.1, 24.1 20.8, 19.7; HRMS (ESI) *m*/*z*: [M + H]^+^calcd for C_22_H_30_N_3_O_6_^+^, 432.2135; found, 432.2129.

#### *N*^2^-Isobutyryl-5′-*O*-(2-methoxypropan-2-yl)-2′-deoxyguanosine (**11**)

To a solution of **8** (175 g, 304 mmol) in THF (875 mL) and 2,2-dimethoxypropane (875 mL), TsOH·H_2_O (5.78 g, 30.4 mmol, 0.1 equiv) was added. After stirring at 10 °C for 1 h, TEA (175 mL) and THF (875 mL) were added into the reaction mixture. The reaction mixture was washed with 5% NaHCO_3_ aqueous solution and separated. The organic phase was concentrated under reduced pressure and the residue was triturated with MTBE to give a white solid (128 g). 120 g (185 mmol) of the solid was dissolved in THF (1.2 L), and TBAF·3H_2_O (116.7 g, 370.4 mmol, 2.0 equiv) was added. After stirring at 6–12 °C for 4 h, DCM (1.8 L) was added into the mixture. The mixture was washed with aqueous 10% NH_4_Cl solution. The combined organic phase was dried over Na_2_SO_4_, filtered, and concentrated under reduced pressure. The crude product was purified by silica gel column chromatography (ethyl acetate/acetone = 4:1, v/v) to give **11** as off-white solid (61 g, yield: 52%). ^1^H NMR (500 MHz, DMSO-*d*_6_): δ 12.07 (s, 1H), 11.68 (s, 1H), 8.19 (s, 1H), 6.23 (t, 1H, *J* = 6.5 Hz), 5.41 (d, 1H, *J* = 4.0 Hz), 4.41–4.38 (m, 1H), 3.95–3.92 (m, 1H), 3.51 (dd, 1H, *J* = 10.5, 4.0 Hz), 3.45 (dd, 1H, *J* = 10.5, 5.5 Hz), 3.02 (s, 3H), 2.80–2.75 (m, 1H), 2.67–2.62 (m, 1H), 2.34–2.30 (m, 1H), 1.26 (s, 6H), 1.13 (d, 6H, *J* = 7.0 Hz); ^13^C{1H} NMR (126 MHz, DMSO-*d*_6_): δ 180.1, 154.8, 148.4, 148.1, 137.3, 120.2, 99.6, 85.8, 82.9, 70.6, 60.9, 47.8, 39.5, 34.8, 24.1, 24.1, 18.8, 18.8; HRMS (ESI) *m*/*z*: [M + H]^+^calcd for C_18_H_28_N_5_O_6_^+^, 410.2040; found, 410.2031.

#### *N*^6^-(2,4-Dimethylbenzoyl)-5′-*O*-(2-methoxypropan-2-yl)-2′-deoxyadenosine Loaded with (−)-Ψ (**1**^**Rp**^)

*N*^6^-(2,4-Dimethylbenzoyl)-5′-*O*-(1-methoxy-1-methylethyl)-2′-deoxyadenosine (**9**, 0.78 g, 1.72 mmol) and (−)-Ψ-reagent (1.00 g, 2.24 mmol) (dried over P_2_O_5_ overnight) were dissolved in anhydrous MeCN (8.0 mL), and DBU (335 μL, 2.24 mmol) was added dropwise. The mixture was stirred at room temperature for 2 h and filtrated through a short silica gel column by eluting with 1% pyridine in EtOAc. The product fractions were combined and washed with sat. aq NaHCO_3_ (50 mL), brine (50 mL), and KH_2_PO_4_ (50 mL). The organic phase was dried over MgSO_4,_ filtrated, and concentrated under vacuum. The crude product was purified by a silica gel chromatography eluting with a mixture of pyridine and EtOAc (1:99, v/v). The product fractions were combined and evaporated to dryness. The residue was co-evaporated twice with DCM to yield the desired product **1**^**Rp**^ as a white foam (0.51 g, 45%). ^1^H NMR (500 MHz, CD_3_CN): δ 9.23 (br s, 1H), 8.65 (s, 1H), 8.36 (s, 1H), 7.53 (d, 1H, *J* = 8.0 Hz), 7.16 (s, 1H), 7.11 (d, 1H, *J* = 8.0 Hz), 6.50 (dd, 1H, *J* = 6.5 and 6.5 Hz), 5.52 (m, 1H), 5.07 (s, 1H), 4.98 (s, 1H), 4.54 (m, 1H), 4.35 (m, 1H), 3.65 (dd, 1H, *J* = 10.5 and 3.5 Hz), 3.62 (dd, 1H, *J* = 10.5 and 4.0 Hz), 3.10 (s, 3H), 3.06 (m, 1H), 2.75 (m, 1H), 2.66 (m, 1H), 2.47 and 2.38 (2× s, 6H), 2.34, 2.12, 2.02–1.94 and 1.85 (m, 6H), 1.83, and 1.70 (2× s, 6H), 1.31 and 1.30 (2× s, 6H). ^13^C{1H} NMR (126 MHz, CD_3_CN): δ 167.9, 152.5, 152,5 150.3, 146.4, 142.5, 141.7, 137.7, 133.0, 132.4, 128.6, 126.8, 124.7, 111.8, 100.8, 86.9, 85.1 (*J* = 8.1 Hz), 84.5, 79.8 (*J* = 7.7 Hz), 66.8, 61.1, 48.6, 39.4, 38.9 (*J* = 2.9 Hz), 34.2 (*J* = 8.8 Hz), 27.9 (*J* = 15.4 Hz), 24.3, 24.2, 23.5, 22.4, 21.7, 21.0, and 19.8; ^31^P NMR (202 MHz, CD_3_CN): δ = 100.65. HRMS (ESI) *m*/*z*: [M + H]^+^ calcd for C_33_H_45_N_5_O_6_PS_2_^+^, 702,2543; found, 702.2534.

#### *N*^6^-(2,4-Dimethylbenzoyl)-5′-*O*-(2-methoxypropan-2-yl)-2′-deoxyadenosine Loaded with (+)-Ψ (**1**^**Sp**^)

**1**^**Sp**^ was synthesized as above (cf. **1**^**Rp**^) using **9** and (+)-Ψ-reagent as starting materials. The product **1**^**Sp**^ was obtained as a white foam (0.83 g, 68%). ^1^H NMR (500 MHz, CD_3_CN): δ 9.44 (br s, 1H), 8.62 (s, 1H), 8.33 (s, 1H), 7.50 (d, 1H, *J* = 7.5 Hz), 7.13 (s, 1H), 7.07 (d, 1H, *J* = 8.0 Hz), 6.50 (dd, 1H, *J* = 6.5 and 6.5 Hz), 5.53 (m, 1H), 5.03 (s, 1H), 4.94 (s, 1H), 4.53 (m, 1H), 4.36 (m, 1H), 3.67 (dd, 1H, *J* = 10.5 and 3.5 Hz), 3.64 (dd, 1H, *J* = 10.5 and 4.5 Hz), 3.09 (s, 3H), 3.03 (m, 1H), 2.73 (m, 1H), 2.65 (m, 1H), 2.46 and 2.36 (2× s, 6H), 2.31, 2.11, 2.01–1.94 and 1.85 (m, 6H), 1.81, and 1.70 (2× s, 6H), 1.31 and 1.30 (2× s, 6H); ^13^C{1H} NMR (126 MHz, CD_3_CN): δ 167.9, 152.5, 152.4 150.3, 146.3, 142.6, 141.7, 137.6, 133.1, 132.3, 128.5, 126.8, 124.7, 111.7, 100.7, 87.0, 85.2 (*J* = 5.4 Hz), 84.6, 80.0 (*J* = 7.8 Hz), 66.7, 61.0, 48.6, 39.4, 38.7 (*J* = 5.4 Hz), 34.2 (*J* = 9.3 Hz), 27.9 (*J* = 15.4 Hz), 24.3, 24.2, 23.5, 22.4, 21.7, 21.0 and 19.8; ^31^P NMR (202 MHz, CD_3_CN): δ = 100.16; HRMS (ESI) *m*/*z*: [M + H]^+^ calcd for C_33_H_45_N_5_O_6_PS_2_^+^, 702,2543; found, 702.2548.

#### *N*^4^-(2,4-Dimethylbenzoyl)-5′-*O*-(2-methoxypropan-2-yl)-2′-deoxycytidine Loaded with (−)-Ψ (**2**^**Rp**^)

**2**^**Rp**^ was synthesized as above (cf. **1**^**Rp**^) using **10** and (−)-Ψ-reagent as starting materials. The product **2**^**Rp**^ was obtained as a white foam in 48% yield. ^1^H NMR (500 MHz, (CD_3_CN): δ 8.98 (br s, 1H), 8.28 (d, 1H, *J* = 7.5 Hz), 7.48 (d, 1H, *J* = 8.0 Hz), 7.45 (d, 1H, *J* = 7.0 Hz), 7.16 (s, 1H), 7.13 (d, 1H, *J* = 8.0 Hz), 6.20 (dd, 1H, *J* = 6.5 and 6.5 Hz), 5.29 (m, 1H), 5.05 (s, 1H), 4.97 (s, 1H), 4.50 (m, 1H), 4.39 (m, 1H), 3.69 (dd, 1H, *J* = 11.0 and 3.0 Hz), 3.64 (dd, 1H, *J* = 11.0 and 3.0 Hz), 3.21 (s, 3H), 2.74 (m, 1H), 2.66 (m, 1H), 2.44 and 2.37 (2× s, 6H), 2.35 (m, 2H), 2.33, 2.10, 1.99–1.93 and 1.85 (m, 5H), 1.83, and 1.69 (2× s, 6H), 1.38 and 1.35 (2× s, 6H); ^13^C{1H} NMR (126 MHz, (CD_3_CN): δ 169.9, 163.3, 155.3, 146.4, 145.1, 142.2, 137.7, 132.6, 132.4, 128.5, 126.9, 111.7, 100.9, 96.1, 87.5, 86.9, 85.5 (*J* = 8.0 Hz), 79.8 (*J* = 7.6 Hz), 66.8, 60.7, 48.9, 40.5 (*J* = 3.4 Hz), 39.4, 34.1 (*J* = 9.1 Hz), 27.8 (*J* = 15.4 Hz), 24.2, 24.2, 23.5, 22.4, 21.7, 20.9 and 19.7; ^31^P NMR (202 MHz, (CD_3_CN): δ 100.62; HRMS (ESI) *m*/*z*: [M + H]^+^ calcd for C_32_H_45_N_3_O_7_PS_2_^+^, 678,2431; found, 678.2439.

#### *N*^4^-(2,4-Dimethylbenzoyl)-5′-*O*-(2-methoxypropan-2-yl)-2′-deoxycytidine Loaded with (+)-Ψ-Reagent (**2**^**Sp**^)

**2**^**Sp**^ was synthesized as above (cf. **1**^**Rp**^) using **10** and (+)-Ψ-reagent as starting materials. The product **2**^**Sp**^ was obtained as a white foam in 65% yield. ^1^H NMR (500 MHz, (CD_3_)_2_CO): δ 9.65 (br s, 1H), 8.39 (d, 1H, *J* = 7.5 Hz), 7.59 (d, 1H, *J* = 7.5 Hz), 7.48 (d, 1H, *J* = 3.5 Hz), 7.15 (s, 1H), 7.13 (d, 1H, *J* = 8.0 Hz), 6.30 (dd, 1H, *J* = 6.5 and 6.5 Hz), 5.39 (m, 1H), 5.05 (s, 1H), 4.97 (s, 1H), 4.55 (m, 1H), 4.46 (m, 1H), 3.79 (dd, 1H, *J* = 11.0 and 3.0 Hz), 3.75 (dd, 1H, *J* = 10.5 and 3.0 Hz), 3.26 (s, 3H), 2.75 (m, 1H), 2.69 (m, 1H), 2.47 and 2.36 (2× s, 6H), 2.41 (m, 1H), 2.33, 2.14, 2.08–1.98 and 1.90 (m, 6H), 1.82 and 1.73 (2× s, 6H), 1.42 and 1.40 (2× s, 6H); ^13^C{1H} NMR (126 MHz, (CD_3_)_2_CO): δ 169.2, 162.9, 154.3, 145.6, 144.3, 141.2, 137.1, 132.2, 131.8, 128.0, 126.3, 111.2, 100.3, 95.5, 86.8, 86.1, 85.1 (*J* = 5.0 Hz), 79.5 (*J* = 7.7 Hz), 65.9, 60.2, 48.3, 39.9 (*J* = 5.9 Hz), 38.9, 33.6 (*J* = 9.1 Hz), 27.4 (*J* = 15.5 Hz), 23.8, 23.7, 23.1, 21.9, 21.1, 20.4 and 19.3; ^31^P NMR (202 MHz, (CD_3_)_2_CO): δ = 99.80; HRMS (ESI) *m*/*z*: [M + H]^+^ calcd for C_32_H_45_N_3_O_7_PS_2_^+^, 678.2431; found, 678.2445.

#### *N*^2^-Isobutyryl-5′-*O*-(2-methoxypropan-2-yl)-2′-deoxyguanosine Loaded with (−)-Ψ (**3**^**Rp**^)

**3**^**Rp**^ was synthesized as above (cf. **1**^**Rp**^) using **11** and (−)-Ψ-reagent as starting materials. The product **3**^**Rp**^ was obtained as a white foam in 61% yield. ^1^H NMR (500 MHz, CD_3_CN): δ 11.90 (br s, 1H), 9.35 (br s, 1H), 7.93 (s, 1H), 6.24 (dd, 1H, *J* = 7.0 and 7.0 Hz), 5.48 (m, 1H), 5.06 (s, 1H), 4.96 (s, 1H), 4.50 (m, 1H), 4.31 (m, 1H), 3.67 (dd, 1H, *J* = 10.5 and 4.0 Hz), 3.60 (dd, 1H, *J* = 10.5 and 4.5 Hz), 3.10 (s, 3H), 3.04 (m, 1H), 2.70 (septet, 1H, *J* = 6.5 Hz), 2.68–2.65 (m, 2H, H2″ and CH); 2.34, 2.06, 2.00–1.91, and 1.84 (m, 6H), 1.81 and 1.70 (2× s, 6H), 1.32 and 1.31 (2× s, 6H), 1.23 (d, 6H, *J* = 6.5 Hz); ^13^C{1H} NMR (126 MHz, CD_3_CN): δ = 180.4, 155.9, 149.1, 148.6, 146.4, 138.1, 121.8, 111.7, 100.8, 87.0, 85.1 (*J* = 7.3 Hz), 84.4, 79.9 (*J* = 7.6 Hz), 66.8, 61.1, 48.6, 39.4, 38.3 (*J* = 3.3 Hz), 36.3, 34.2 (*J* = 8.8 Hz), 27.9 (*J* = 15.5 Hz), 24.3, 24.2, 23.55, 22.4, 21.7, 18.8 and 18.7; ^31^P NMR (202 MHz, CD_3_CN): δ = 100.85; HRMS (ESI) *m*/*z*: [M + H]^+^ calcd for C_28_H_43_N_5_O_7_PS_2_^+^, 656.2336; found, 656.2326.

#### *N*^2^-Isobutyryl-5′-*O*-(2-methoxypropan-2-yl)-2′-deoxyguanosine Activated with (+)-Ψ (**3**^**Sp**^)

**3**^**Sp**^ was synthesized as above (cf. **1**^**Rp**^) using **11** and (+)-Ψ-reagent as starting materials. The product **3**^**Sp**^ was obtained as a white foam in 45% yield. ^1^H NMR (500 MHz, CD_3_CN): δ 12.01 (br s, 1H), 9.59 (br s, 1H), 7.95 (s, 1H), 6.25 (dd, 1H, *J* = 7.0 and 7.0 Hz), 5.46 (m, 1H), 5.03 (s, 1H), 4.93 (s, 1H), 4.48 (m, 1H), 4.31 (m, 1H), 3.68 (dd, 1H, *J* = 10.5 and 4.0 Hz), 3.62 (dd, 1H, *J* = 10.5 and 4.5 Hz), 3.11 (s, 3H), 2.99 (m, 1H), 2.72 (septet, 1H, *J* = 6.5 Hz), 2.68–2.64 (m, 2H), 2.31, 2.08, 2.01–1.91, and 1.84 (m, 6H), 1.80 and 1.70 (2× s, 6H), 1.32 and 1.31 (2× s, 6H), 1.22 (d, 6H, *J* = 6.5 Hz); ^13^C{1H} NMR (126 MHz, CD_3_CN): δ 180.5, 155.9, 149.0, 148.6, 146.4, 138.0, 121.7, 111.7, 100.8, 87.1, 85.3 (*J* = 5.4 Hz), 84.6, 80.2 (*J* = 8.1 Hz), 66.7, 61.0, 48.6, 39.4, 38.4 (*J* = 5.3 Hz), 36.3, 34.2 (*J* = 8.8 Hz), 27.9 (*J* = 15.4), 24.3, 24.2, 23.5, 22.4, 21.7, 18.8 and 18.8; ^31^P NMR (202 MHz, CD_3_CN): δ = 100.39; HRMS (ESI) *m*/*z*: [M + H]^+^ calcd for C_28_H_43_N_5_O_7_PS_2_^+^, 656.2336; found, 656.2333.

#### 5′-*O*-(2-Methoxypropan-2-yl)-thymidine Loaded with (−)-PSI-Reagent (**4**^**Rp**^)

**4**^**Rp**^ was synthesized as above (cf. **1**^**Rp**^) using **12** (−)-PSI-regent as starting materials. The product **4**^**Rp**^ was obtained as a white foam in 45% yield. ^1^H NMR (500 MHz, CDCl_3_): δ 8.01 (s, 1H), 7.63 (d, 1H, *J* = 1.50 Hz); 6.45 (dd, 1H, *J* = 9.0 and 5.5 Hz), 5.40 (m, 1H), 5.10 (s, 1H), 4.94 (s, 1H), 4.50 (m, 1H), 4.38 (m, 1H), 3.75 (dd, 1H, *J* = 11.0 and 2.5 Hz), 3.69 (dd, 1H, *J* = 10.5 and 2.0 Hz), 3.27 (s, 3H), 2.63 (m, 1H), 2.53 (m, 1H), 2.36 (m, 1H), 2.24 (m, 1H), 2.16 (m, 1H), 2.01–1.79 (m, 4H); 1.96 (d, 3H, *J* = 1.0 Hz), 1.83 (s, 3H), 1.73 (s, 3H), 1.43 (s, 6H); ^13^C{1H} NMR (126 MHz, CDCl_3_): δ 163.2, 150.0, 144.7, 135.3, 112.2, 111.1, 100.5, 86.1, 84.8 (*J* = 12.5 Hz), 84.6, 79.9 (*J* = 7.4 Hz), 66.0, 60.9, 49.0, 39.4 (*J* = 4.4 Hz), 38.8, 33.6 (*J* = 9.6 Hz), 27.7 (15.5 Hz), 24.5, 24.4, 23.4, 22.6, 21.8, 12.5; ^31^P NMR (202 MHz, CDCl_3_): δ 101.72 ppm. ESI^+^-HRMS *m*/*z*: [M + Na]^+^ calcd for C_24_H_37_N_2_O_7_PS_2_Na^+^, 583.1672; found, 583.1689.

#### 5′-*O*-(2-Methoxypropan-2-yl)-thymidine Loaded with (+)-PSI-Reagent (**4**^**Sp**^)

**4**^**Sp**^ was synthesized as above (cf. **1**^**Rp**^) using **12** and (+)-PSI-reagent as starting materials. The product **4**^**Sp**^ was obtained as a white foam in 69% yield. ^1^H NMR (500 MHz, CDCl_3_): δ 8.38 (s, 1H), 7.61 (d, 1H, *J* = 1.0 Hz), 6.45 (dd, 1H, *J* = 9.0 and 5.5 Hz), 5.39 (m, 1H), 5.07 (s, 1H), 4.91 (s, 1H), 4.48 (m, 1H), 4.37 (m, 1H), 3.75 (dd, 1H, *J* = 11.0 and 2.50 Hz), 3.68 (dd, 1H, *J* = 11.0 and 2.5 Hz), 3.26 (s, 3H), 2.60 (m, 1H), 2.50 (m, 1H), 2.30 (m, 1H), 2.22–2.12 (m, 2H), 1.99, 1.91–1.83 and 1.77 (m, 4H), 1.94 (d, 3H, *J* = 0.50 Hz), 1.81 (s, 3H), 1.71 (s, 3H), 1.41 (s, 6H); ^13^C{1H} NMR (126 MHz, CDCl_3_): δ 163.3, 150.2, 144.7, 135.3, 112.2, 111.2, 100.5, 86.1, 84.9, 84.8 (*J* = 14.5 Hz), 80.1 (*J* = 7.3 Hz), 65.8, 60.8, 49.0, 39.1 (*J* = 7.6 Hz), 38.8, 33.7 (*J* = 9.2 Hz), 27.8 (*J* = 15.9 Hz), 24.5, 24.4, 23.4, 22.7, 21.7, 12.5; ^31^P NMR (202 MHz, CDCl_3_): δ = 101.59 ppm. ESI^+^-HRMS *m*/*z*: [M + Na]^+^ calcd for C_24_H_37_N_2_O_7_PS_2_Na^+^, 583.1672; found, 583.1689.

### General Procedure for LPOS

#### a/Synthesis Cycle

The tetrapodal nucleoside construct (cf. **13**, 50 μmol), the Ψ -activated 2′-deoxynucleoside building block (**1–4**^**Rp/Sp**^, 0.33 mmol), and DBU (83 μL, 0.54 mmol) were dissolved in a mixture of pyridine/MeCN (2:3, v/v, 1.9 mL) under nitrogen. The reaction mixture was stirred for 15 min–1 h (at r.t.) and then acetic acid (36 μL, 0.60 mmol) was added.

#### b/Synthesis Cycle

The coupling mixture was added dropwise to cold (4 °C) 2-propanol (30 mL), resulting in white precipitation. The mixture was centrifugated, the 2-propanol supernatant was decanted off, and the precipitate was dried under vacuum.

#### c/Synthesis Cycle

The precipitate was dissolved in a mixture of DCA-dichloromethane/methanol (0.12:2:1, v/v, 1.6 mL). The mixture was stirred for 3–6 min (at r.t.) and neutralized by addition of pyridine (50 μL, 2 equiv compared to DCA).

#### d/Synthesis Cycle

The deprotection mixture was added dropwise to cold (4 °C) 2-propanol (30 mL), resulting in white precipitation. The mixture was centrifugated, the 2-propanol supernatant was decanted off, and the precipitate was dried under vacuum. By using this synthesis cycle and repeating it (*n* = 2 and 3), white powder of protected tetrapodal di-, tri-, and tetranucleotides were obtained in 95–98, 81–89, and 76–86% yields, respectively.

#### Release/Deprotection

The powders were dissolved in concentrated (25%) aqueous ammonia (2 mL) and the mixtures were incubated at 55 °C for 3 h (**14**, **15**, **18–21**) or overnight (**16**, **17**, **22–25**). The precipitated traces of the soluble support, i.e., tetrakis[(4-{[4-(3-amino-3-oxopropyl)-1*H*-1,2,3-triazol-1-yl-]methyl}phenoxy)-methyl]methane,^28^ was filtered off and the filtrate was evaporated to dryness. The residue was dissolved in water, washed with ethyl acetate, and subjected then to a RP HPLC analysis (Figure 2). The yields of the nucleotides (**14–17**: 74–81%, **18–23**: 56–66% and **24** and **25**: 59 and 53%) were determined according to UV-absorbance at λ = 260 nm. HRMS (ESI) *m*/*z*: [M – H]^−^: **14** and **15**: calcd, 561.1062; found, 561.1067 and 561.1069, **16** and **17**: calcd, 570.1178; found, 570.1169 and 570.1179, **18**–**21**: calcd, 881.1294; found, 881.1295, 881.1291, 881.1298 and 881.1298, in this order, **22** and **23**: calcd, 915.1474; found, 915.1494 and 915.1509, **24** and **25**: calcd, 1220.1719; found, 1220.1702 and 1220.1712; ^31^P NMR (200 MHz, D_2_O) of **14–25**: Figure 1.

### Enzymatic Hydrolysis

To confirm the stereochemical integrity of the phosphodiester linkages, nucleotides **16**, **17**, **22–25** were exposed to phosphodiesterase I extracted from venom of *Crotalus admanteus* (svPDE). The enzymatic reactions were carried out in sealed tubes immersed in an aluminum dry block heater. The enzymatic hydrolysis was followed in a 0.1 M Tris–HCl buffer (234 μL) at pH 8.5 and at 37 °C in the presence of svPDE (60 μL) and 0.15 mM MgCl_2_ (4.5 μL). The initial phosphorothioate substrate concentration was 0.33 mM. The aliquots (50 μL) withdrawn from the reaction solution (300 μL) were diluted with a 100 μL mixture of 50 mM TEAA buffer and filtered with minisart RC4 filters (0.2 μm). The composition of the samples was analyzed by RP-HPLC (Figures S61–S63).

## Data Availability

The data underlying this study are available in the published article and its Supporting Information.

## Supporting Information Available

^1^H and ^13^C NMR data for **1–4**^**Rp/Sp**^, **9–11**; synthesis and characterization data for **6** and **7**; and examples of RP HPLC profiles of the coupling reactions, crude products, and of the enzymatic degradation of **16**, **17**, and **22–25** (PDF)
