# Supplementary material for: Stereo-Controlled Liquid Phase Synthesis of Phosphorothioate Oligonucleotides on a Soluble Support
Source: J Org Chem. 2023 Jul 10;88(14):10156–63. doi: 10.1021/acs.joc.3c01006 (PMC10367069; doi:10.1021/acs.joc.3c01006)
Supplement: Supplementary file 1 — jo3c01006_si_001.pdf [file jo3c01006_si_001.pdf]

Supporting information to:

Stereo-controlled Liquid Phase Synthesis of Phosphorothioate Oligonucleotides on a Soluble Support

Petja Rosenqvist,[a] Verneri Saari,[a] Ella Pajuniemi,[a] Alejandro Gimenez Molina,[b] Mikko Ora,[a,*] Andras Horvath,[b] Pasi Virta[a,*]

[a]Department of Chemistry, University of Turku, 20500 Turku, Finland

[b]Chemical Process Research & Development, Janssen Pharmaceutical Companies of Johnson & Johnson, 2340 Beerse, Belgium

Contents:

**^{1}H ^{13}C and ^{31}P NMR spectra of 1^{Rp}**	S2-S4
**^{1}H ^{13}C and ^{31}P NMR spectra of 1^{Sp}**	S5-S7
**^{1}H ^{13}C and ^{31}P NMR spectra of 2^{Rp}**	S8-S10
**^{1}H ^{13}C and ^{31}P NMR spectra of 2^{Sp}**	S11-S13
**^{1}H ^{13}C and ^{31}P NMR spectra of 3^{Rp}**	S14-S16
**^{1}H ^{13}C and ^{31}P NMR spectra of 3^{Sp}**	S17-S19
**^{1}H ^{13}C and ^{31}P NMR spectra of 4^{Rp}**	S20-S22
**^{1}H ^{13}C and ^{31}P NMR spectra of 4^{Sp}**	S23-S25
**^{1}H and ^{13}C and spectra of 9**	S26
**^{1}H and ^{13}C and spectra of 10**	S27
**^{1}H and ^{13}C and spectra of 11**	S28
**Synthesis of *N*^{6}-(2,4-dimethylbenzoyl)-3'-*O*-tert-butyldimethylsilyl-2'-deoxyadenosine (6)**	S29
**^{1}H and ^{13}C and spectra of 6**	S30
**Synthesis of *N*^{4}-(2,4-dimethylbenzoyl)-3'-*O*-tert-butyldimethylsilyl-2'-deoxycytidine (7)**	S31
**^{1}H and ^{13}C and spectra of 7**	S32
**RP HPLC profiles of the coupling reactions with 4^{Rp} and 5^{Rp} on soluble support 13**	S33
**RP HPLC profiles of 5'-deacetalization and detritylation**	S34
**RP HPLC profiles of crude product (16,17,22-24) mixtures**	S35
**Product distribution of enzyme-catalyzed hydrolysis of 16 and 17**	S36
**Product distribution of enzyme-catalyzed hydrolysis of 22 and 23**	S37
**Product distribution of enzyme-catalyzed hydrolysis of 24 and 25**	S38

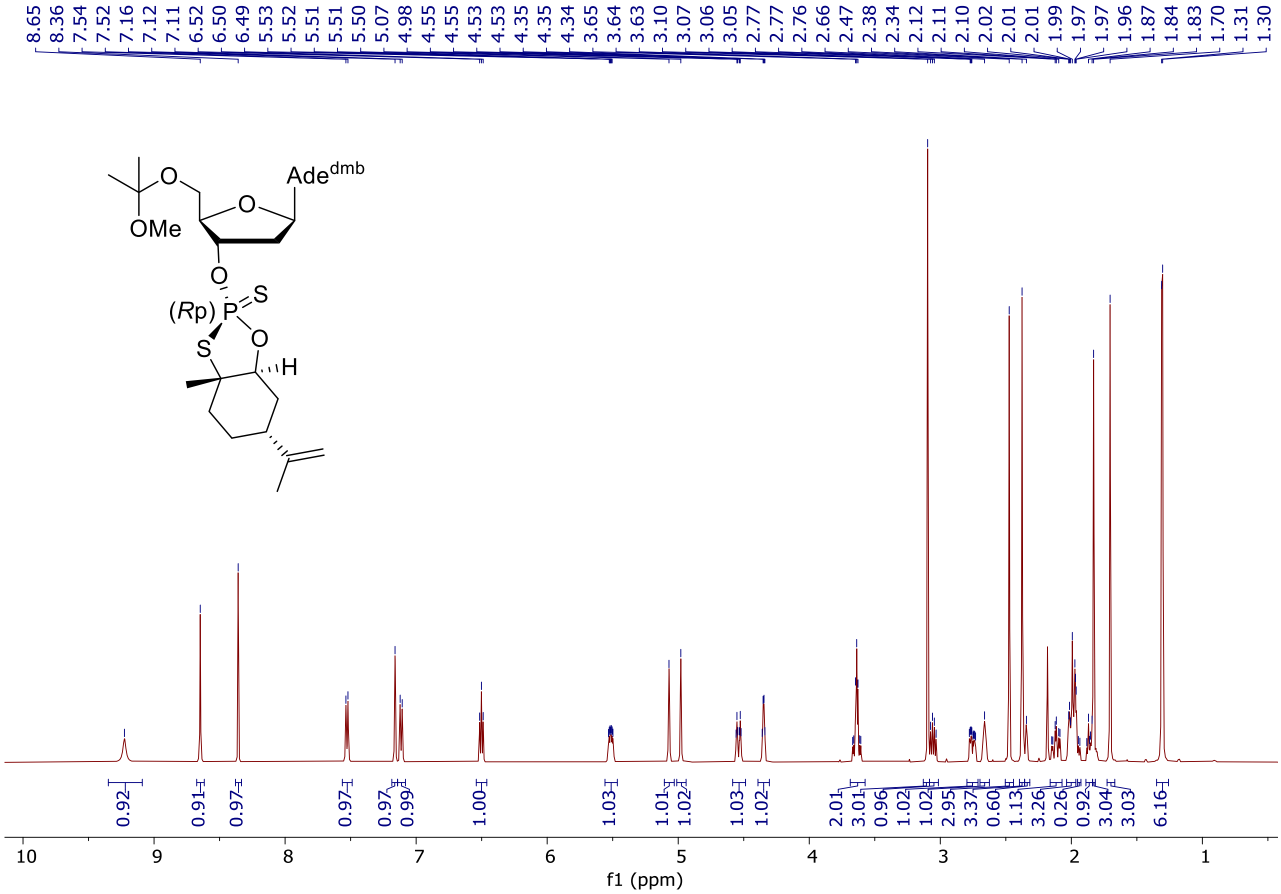

Figure S1. ^{1}H NMR (500 MHz, CD^{3}CN) spectrum of **1*^{Rp}***.

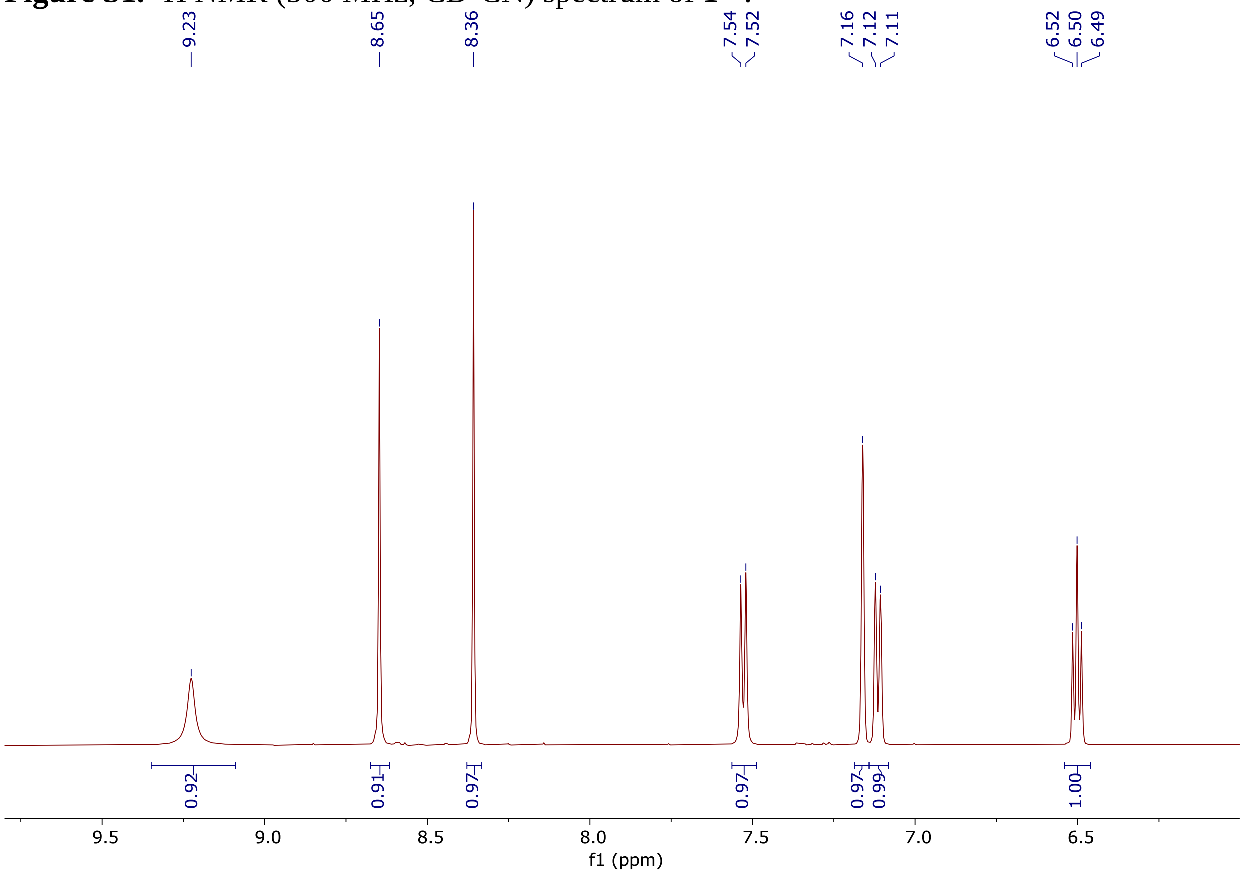

Figure S2. Magnified region (δ_H = 9.8 ppm to 6.0 ppm) of the ^{1}H NMR spectrum of **1*^{Rp}***.

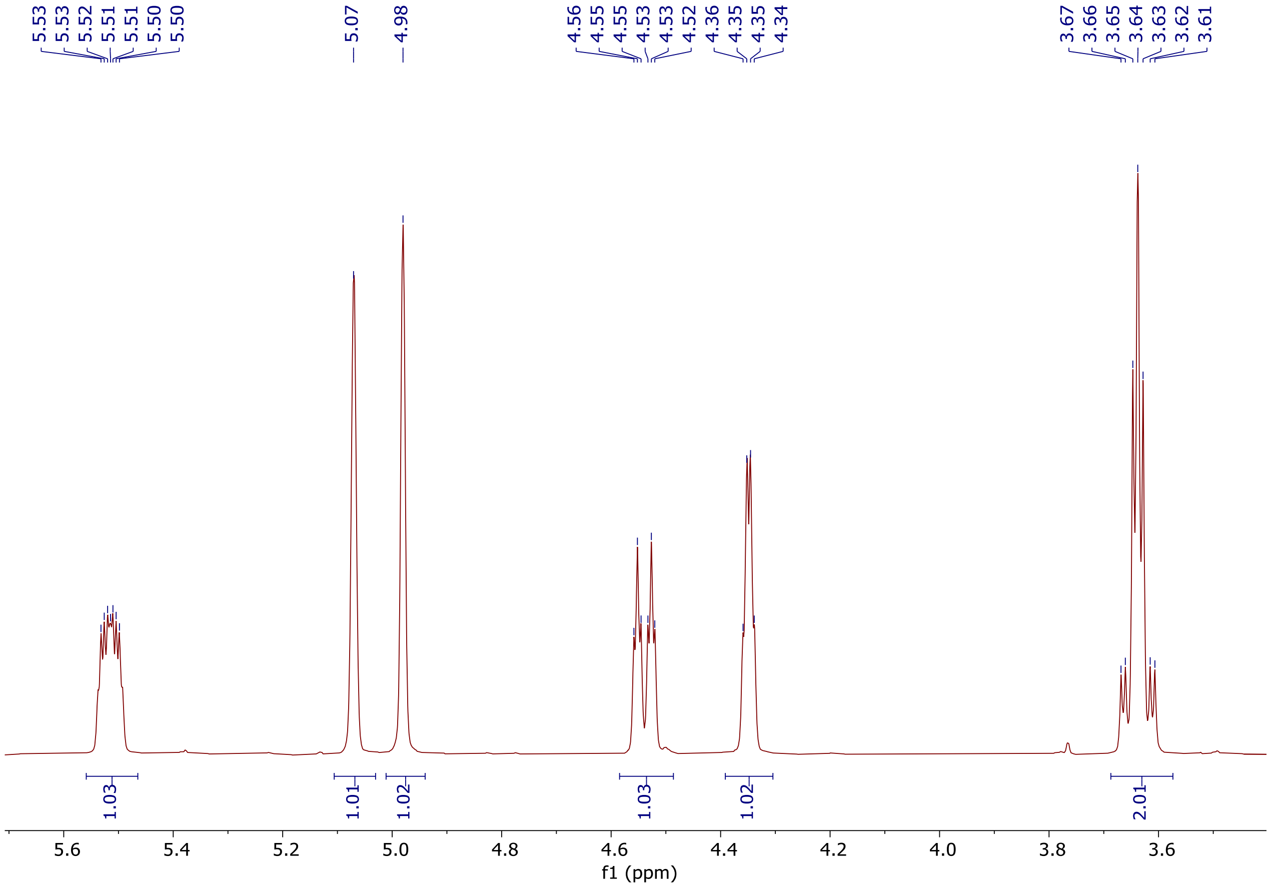

Figure S3. Magnified region (δ_H = 5.7 ppm to 3.4 ppm) of the ^{1}H NMR spectrum of $\mathbf{1}^{Rp}$.

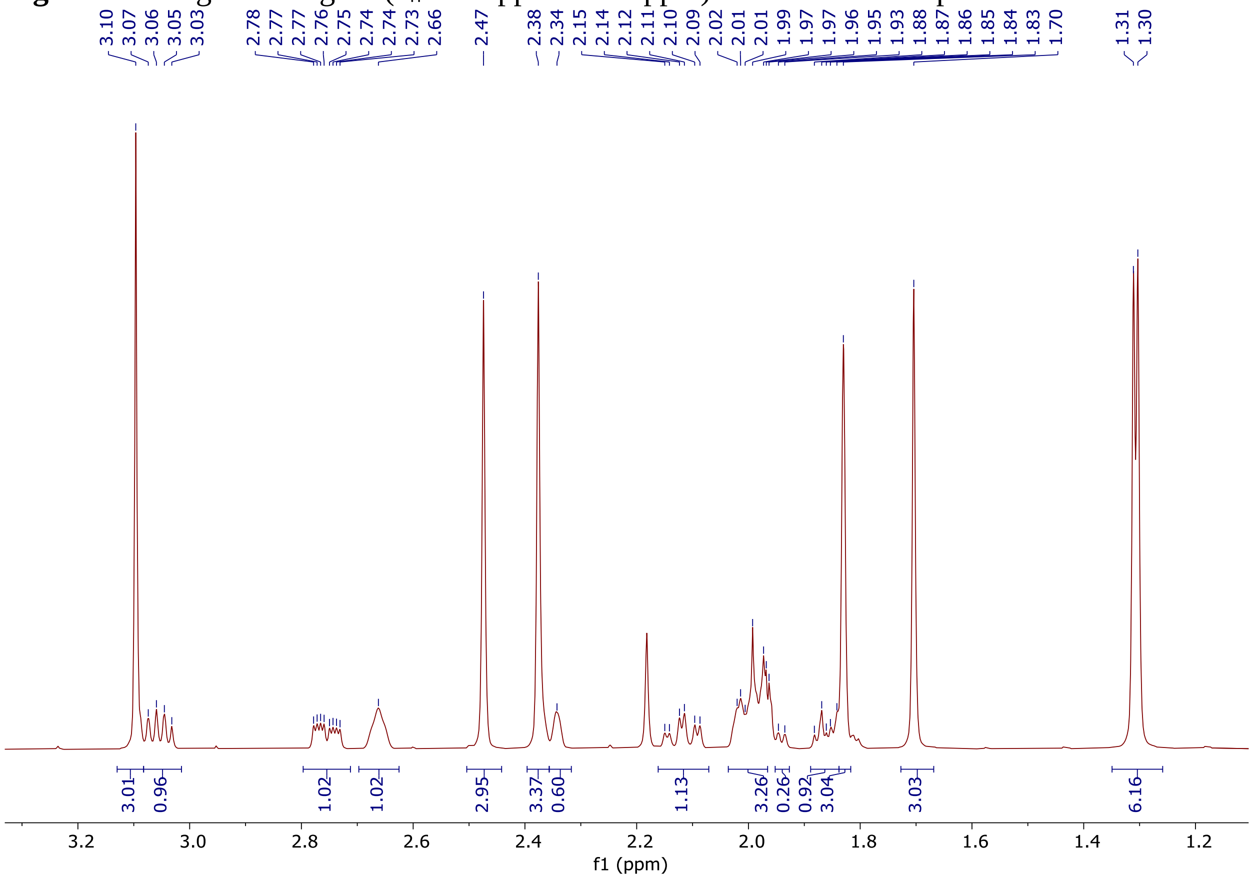

Figure S4. Magnified region (δ_H = 3.3 ppm to 1.1 ppm) of the ^{1}H NMR spectrum of $\mathbf{1}^{Rp}$.

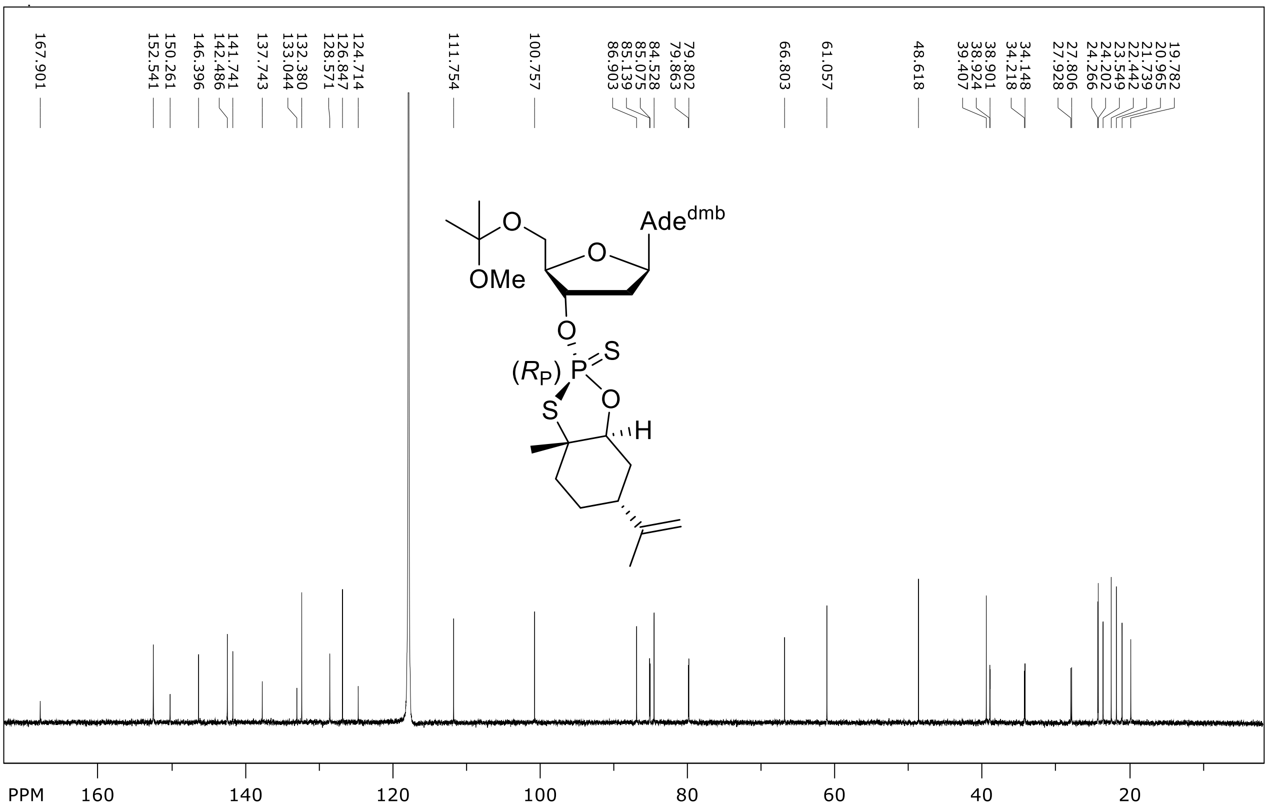

Figure S5. ^{13}C NMR (126 MHz, CD^{3}CN) spectrum of **1*^Rp^***.

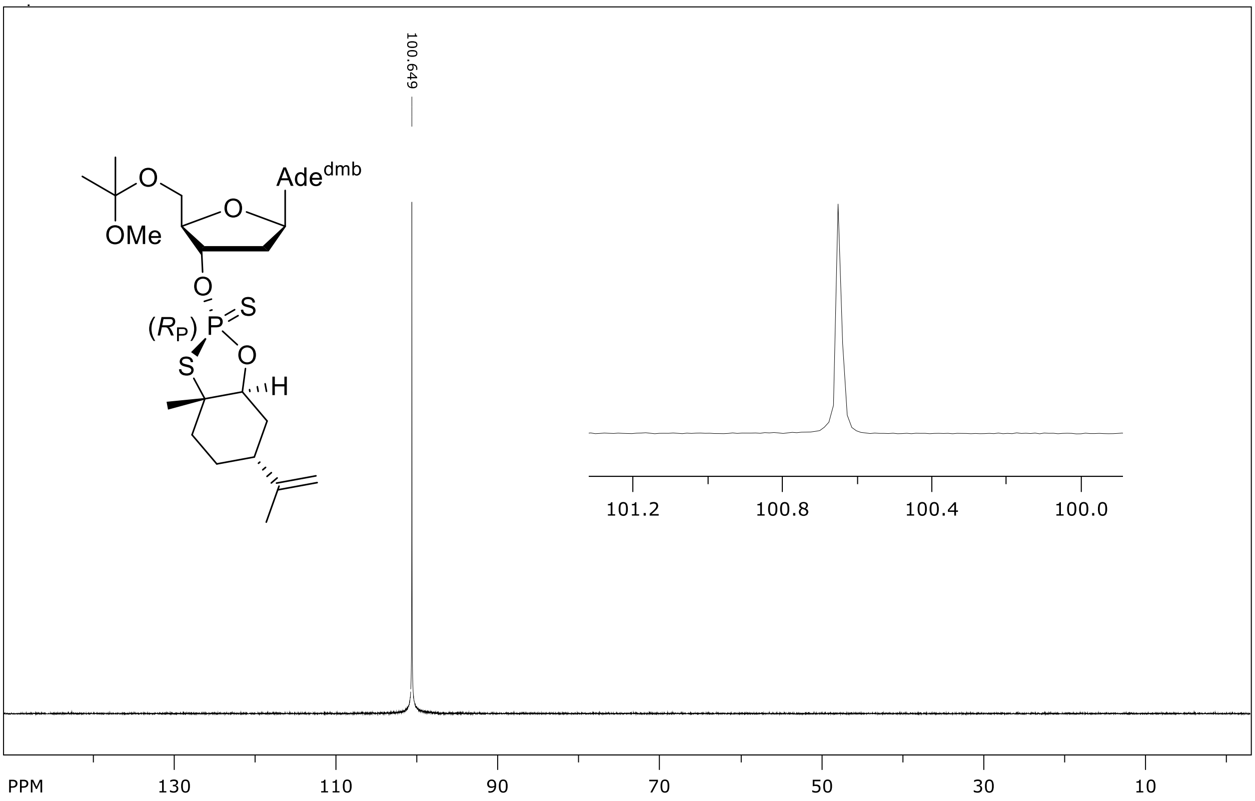

Figure S6. 202 MHz (CD$_3$CN) ^{31}P NMR (202 MHz, CD$_3$CN) spectrum of **1*^Rp^***.

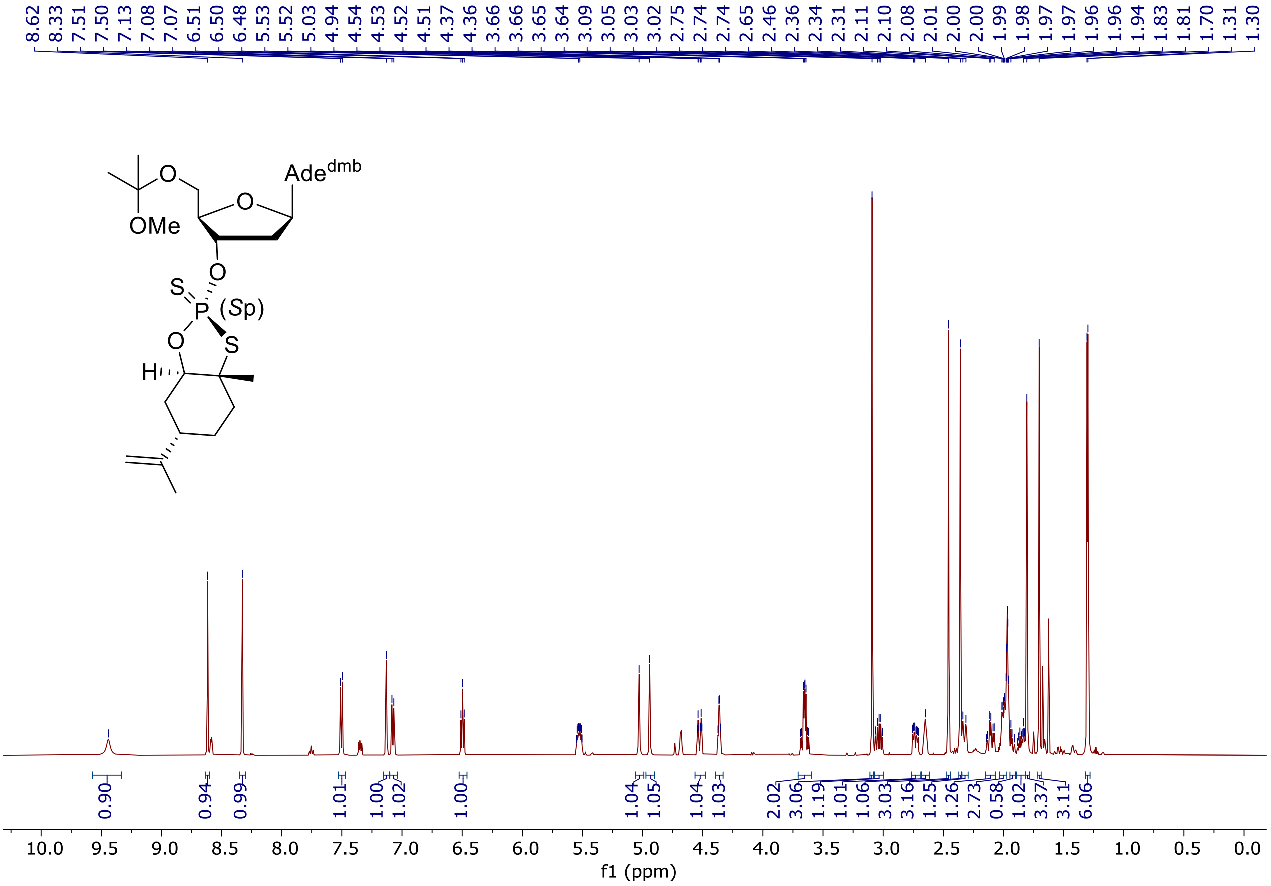

Figure S7. ^{1}H NMR (500 MHz, CD^3CN) spectrum of $\mathbf{1}^{\boldsymbol{Sp}}$.

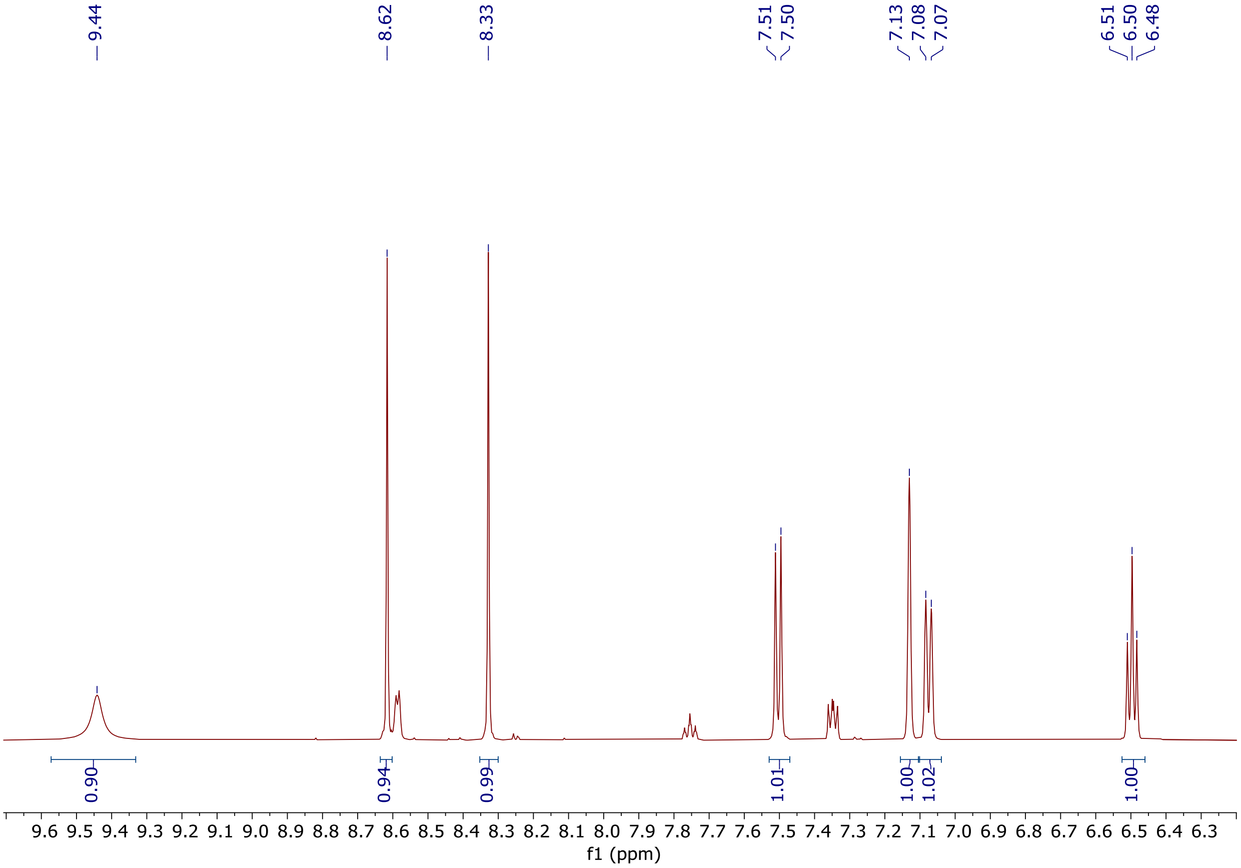

Figure S8. Magnified region (δ_H = 9.7 ppm to 6.2 ppm) of the ^{1}H NMR spectrum of $\mathbf{1}^{\boldsymbol{Sp}}$.

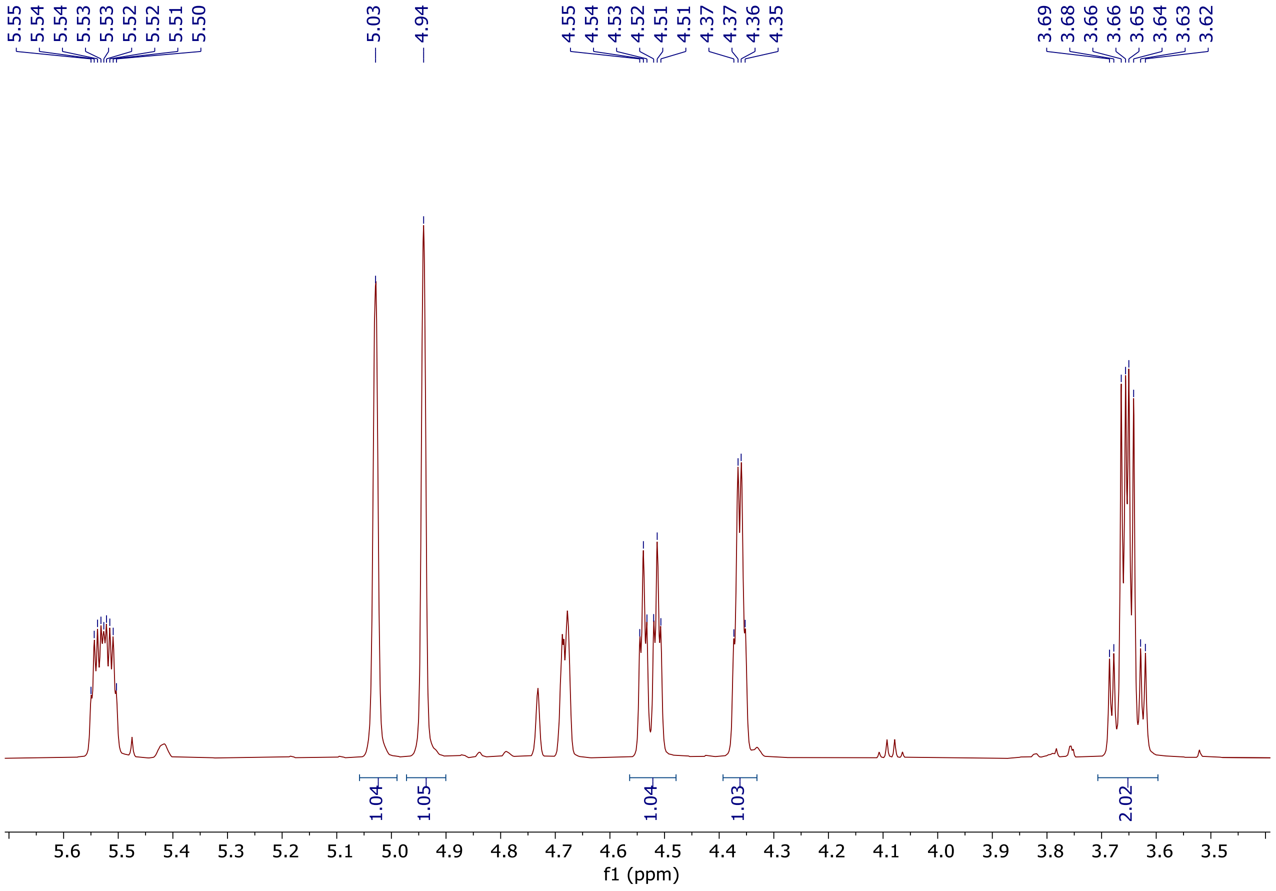

Figure S9. Magnified region (δ_H = 5.7 ppm to 3.4 ppm) of the ^{1}H NMR spectrum of $\mathbf{1}^{Sp}$.

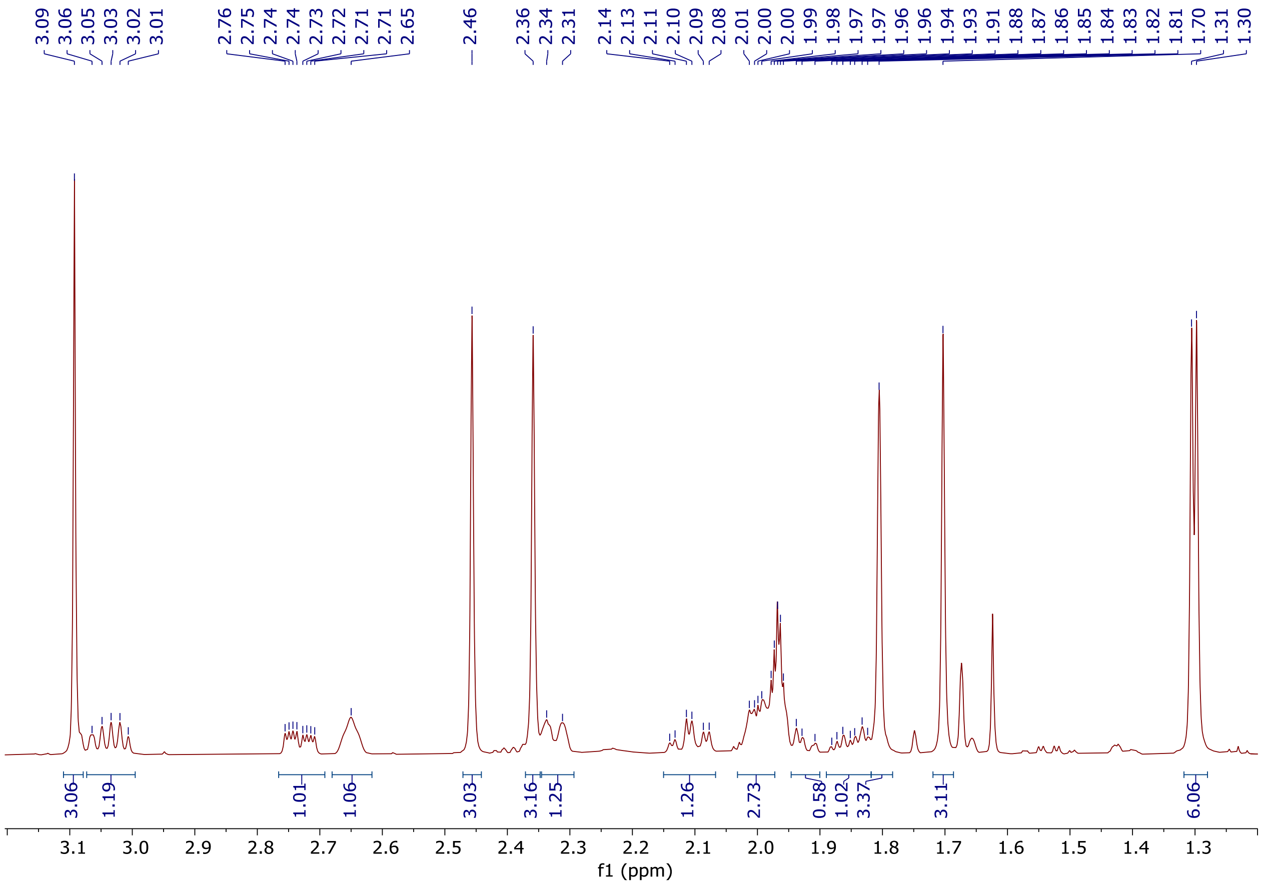

Figure S10. Magnified region (δ_H = 3.2 ppm to 1.2 ppm) of the ^{1}H NMR spectrum of $\mathbf{1}^{Sp}$.

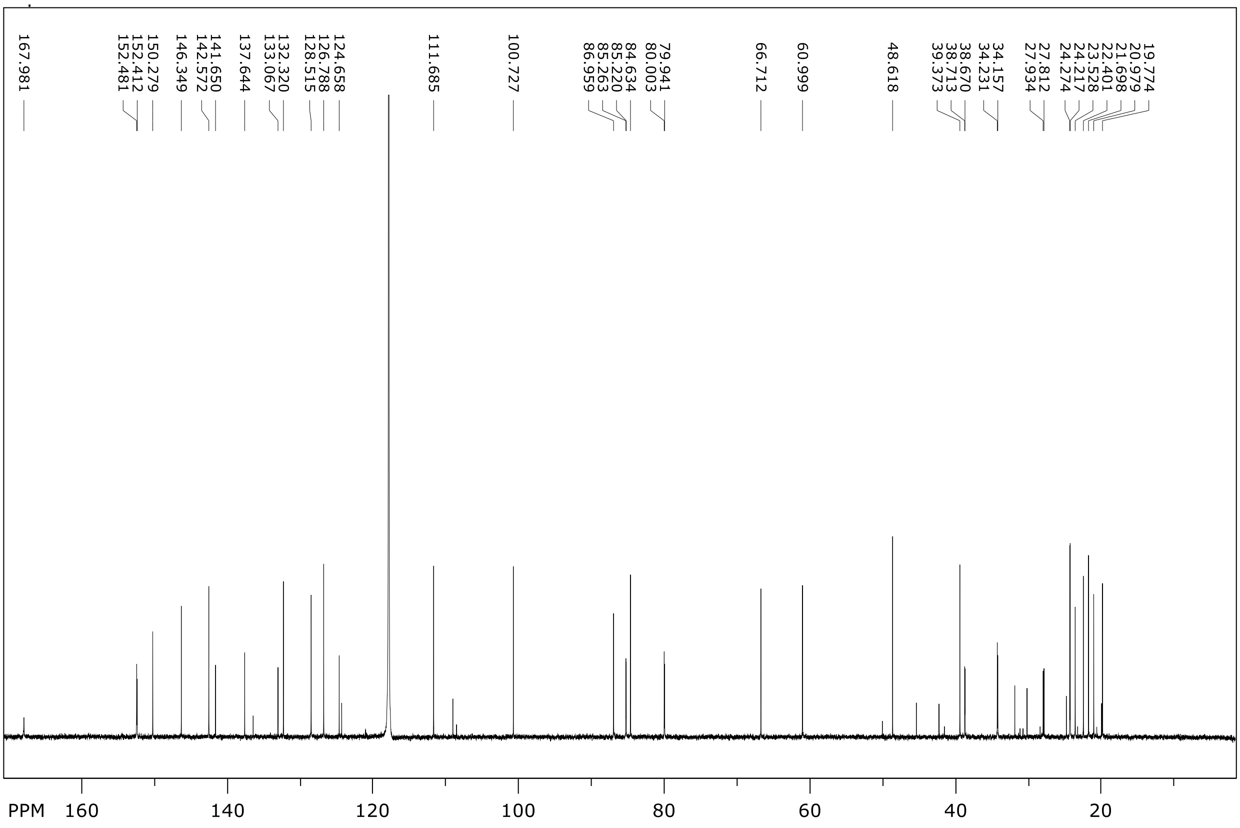

Figure S11. ^{13}C NMR (126 MHz, CD_3CN) spectrum of **1**^{Sp}.

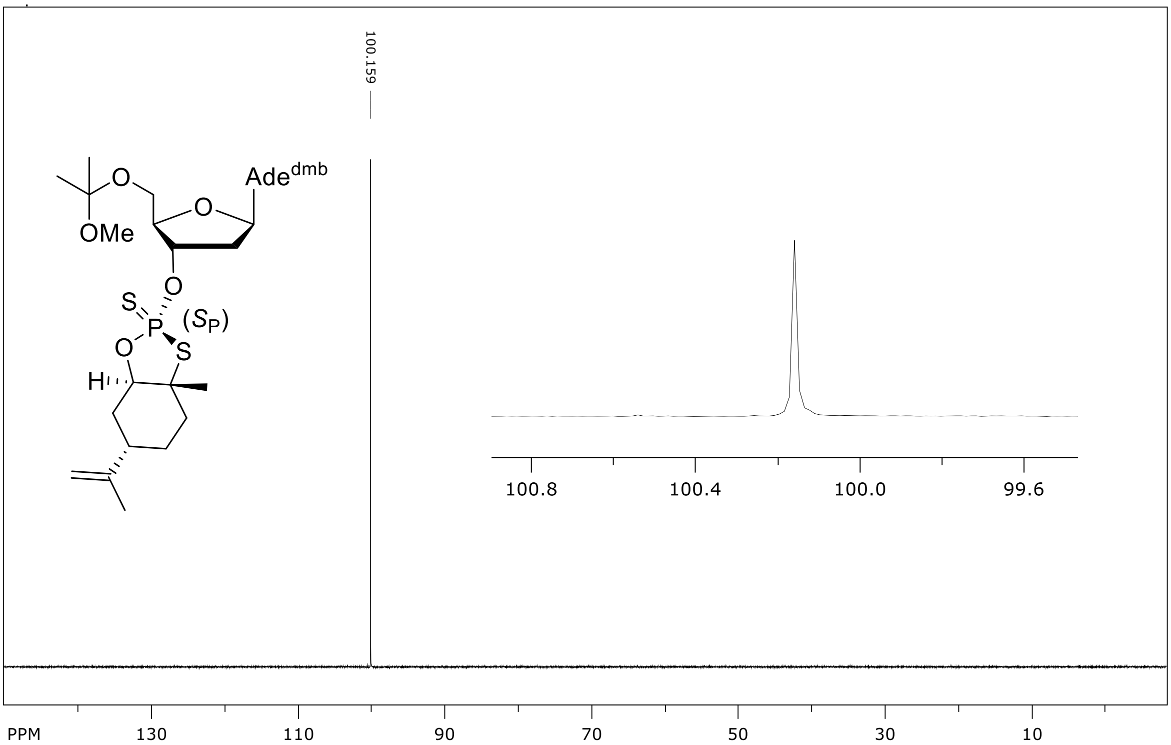

Figure S12. ^{31}P NMR (202 MHz, CD_3CN) spectrum of **1**^{Sp}.

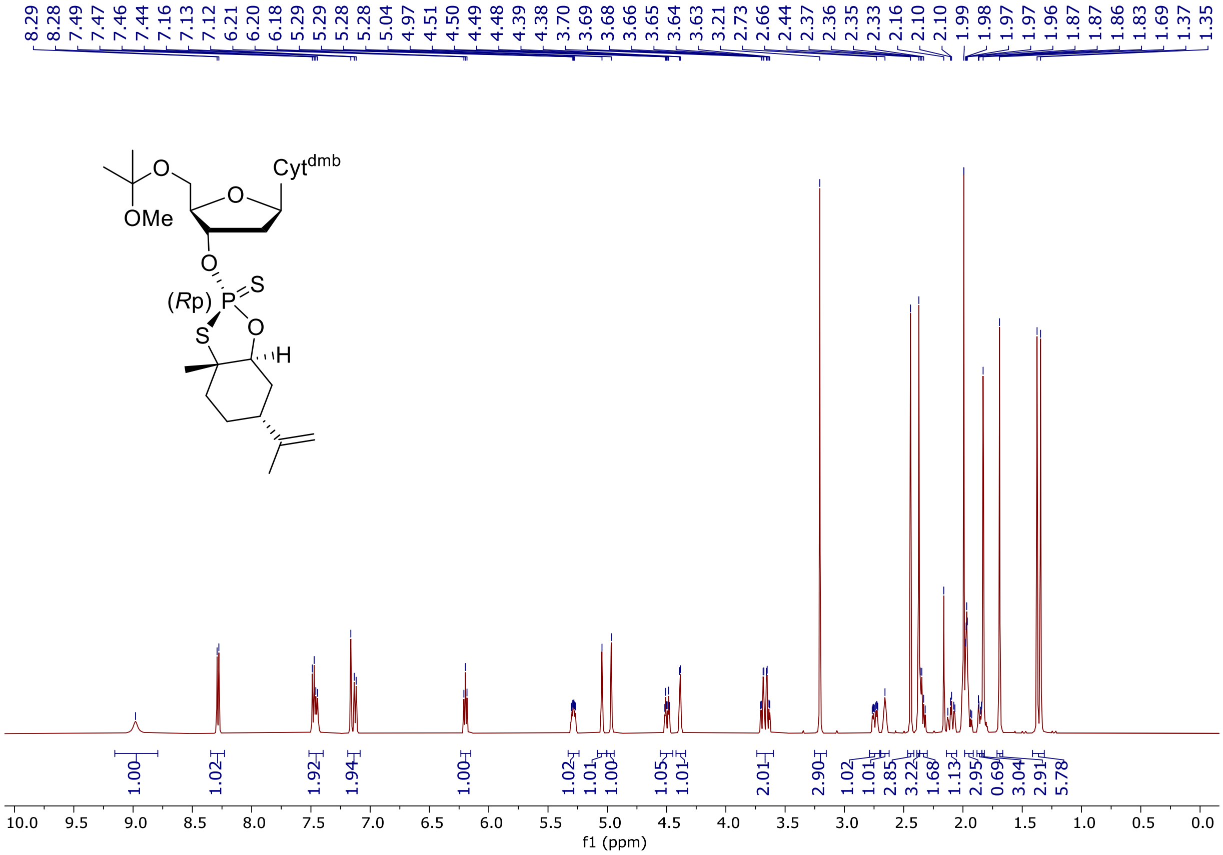

Figure S13. ^{1}H NMR (500 MHz, CD_3CN) spectrum of $\mathbf{2}^{\boldsymbol{Rp}}$.

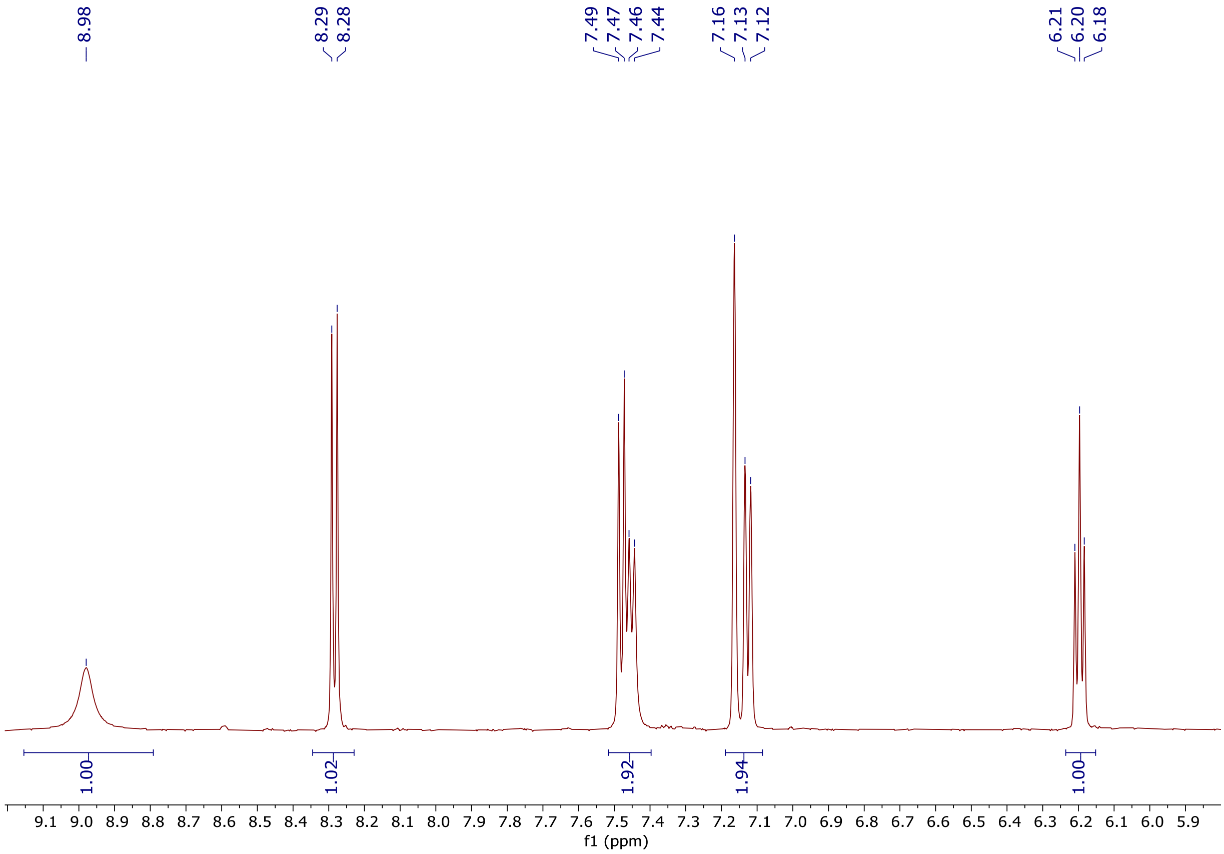

Figure S14. Magnified region (δ_H = 9.2 ppm to 5.8 ppm) of the ^{1}H NMR spectrum of $\mathbf{2}^{\boldsymbol{Rp}}$.

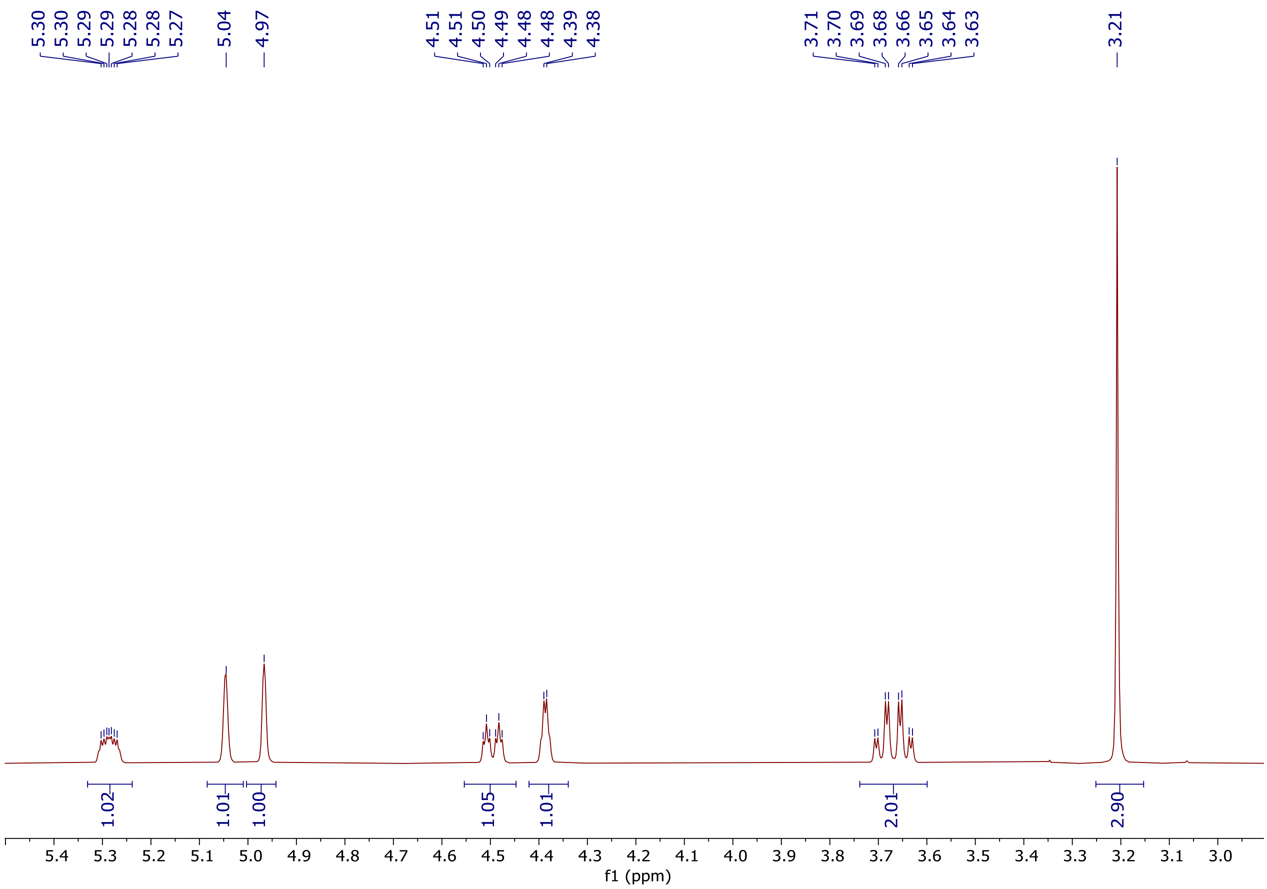

Figure S15. Magnified region (δ_H =5.5 ppm to 2.9 ppm) of the ^{1}H NMR spectrum of $\mathbf{2}^{\mathbf{\mathit{Rp}}}$.

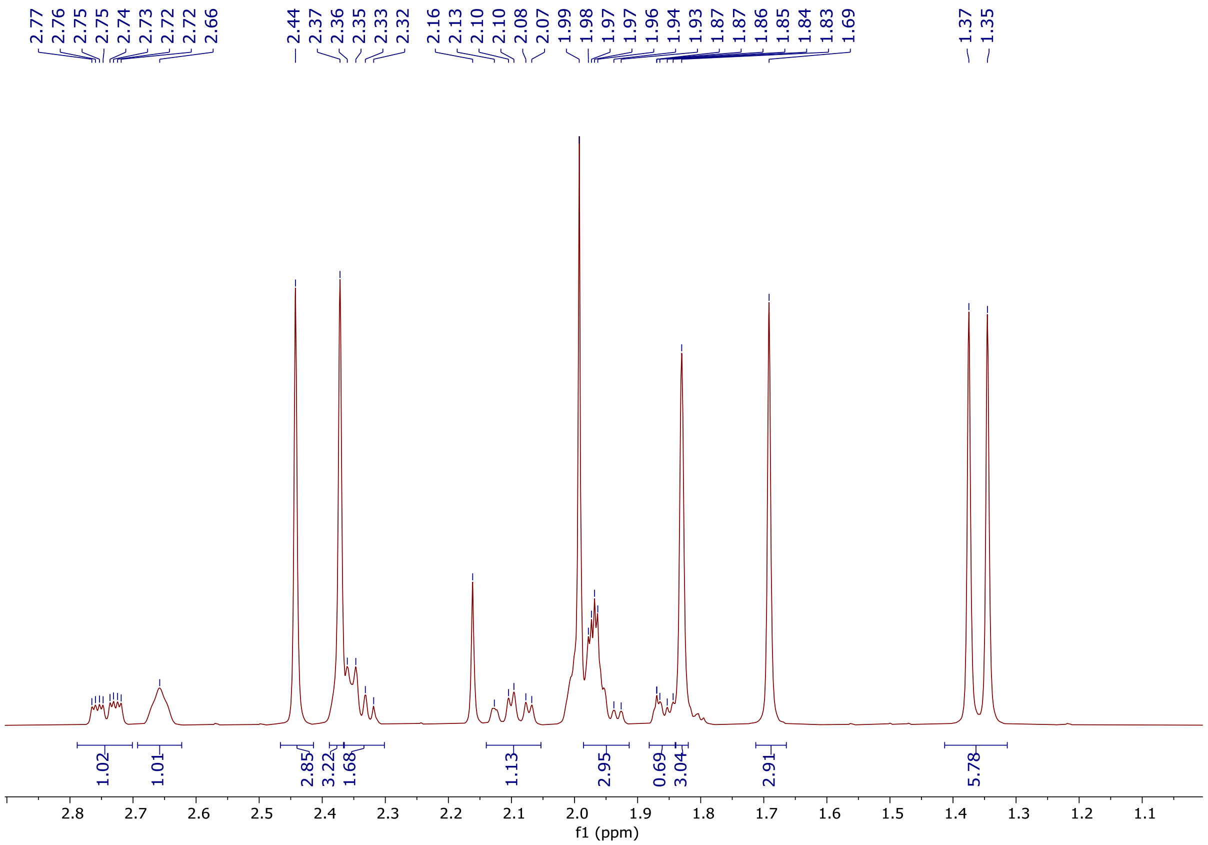

Figure S16. Magnified region (δ_H = 2.9 ppm to 1.0 ppm) of the ^{1}H NMR spectrum of $\mathbf{2}^{\mathbf{\mathit{Rp}}}$.

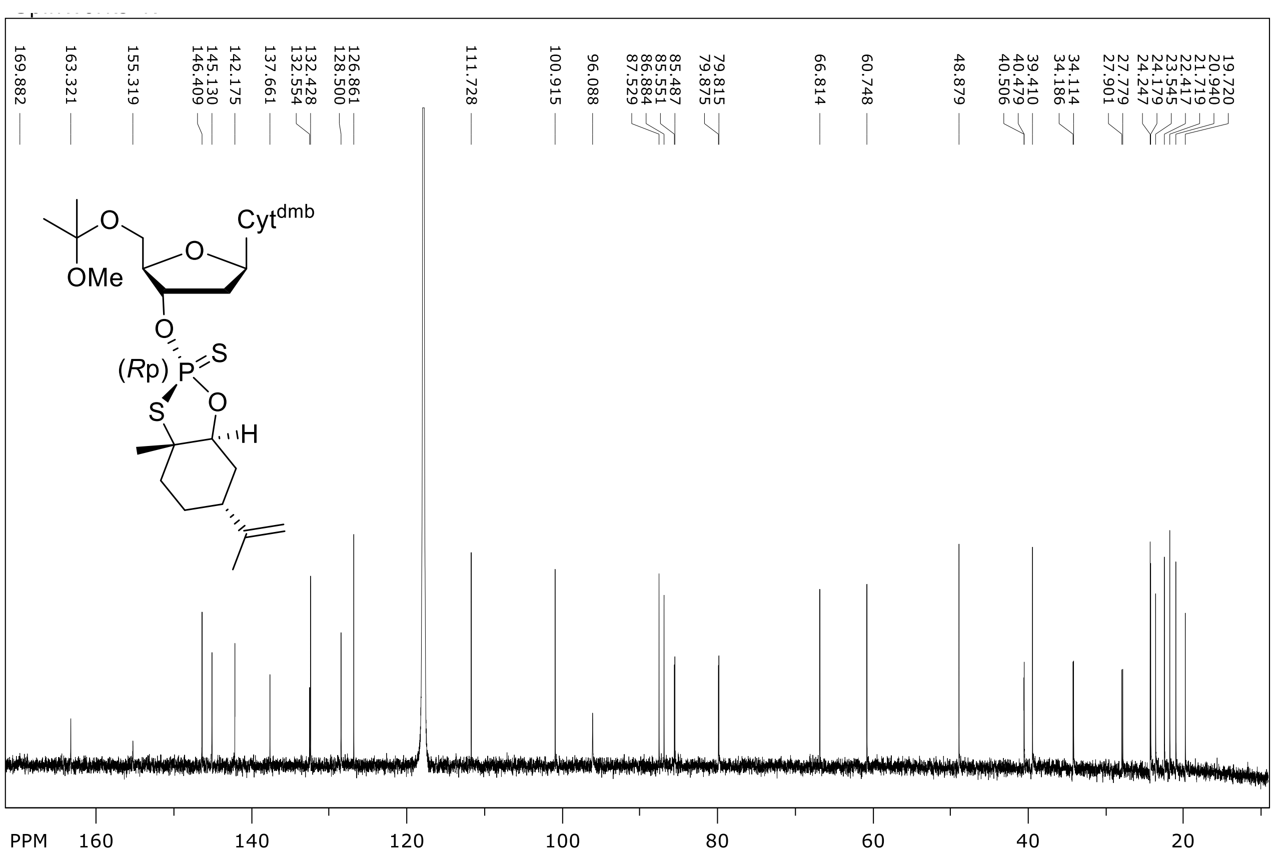

Figure S17. ^{13}C NMR (126 MHz, CD_3CN) spectrum of $\mathbf{2}^{\boldsymbol{Rp}}$.

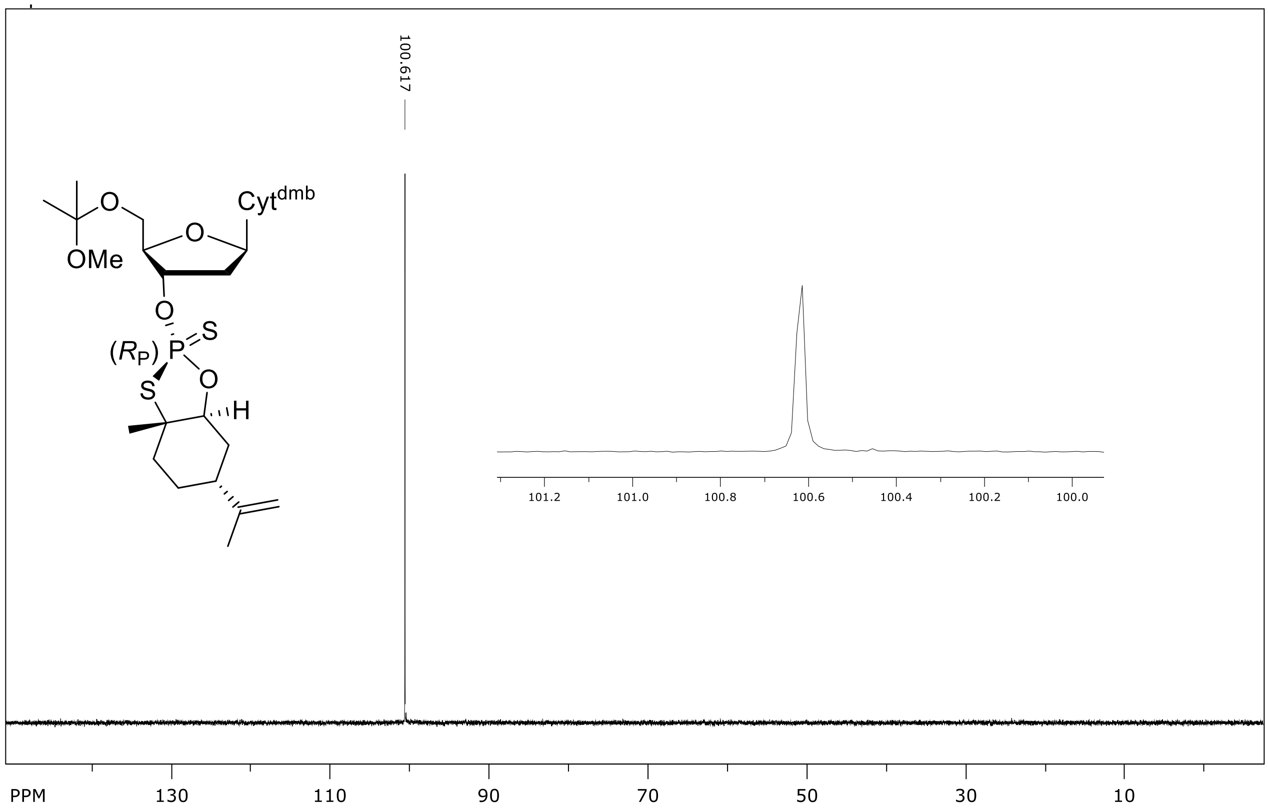

Figure S18. ^{31}P NMR (202 MHz, CD_3CN) spectrum of $\mathbf{2}^{\boldsymbol{Rp}}$

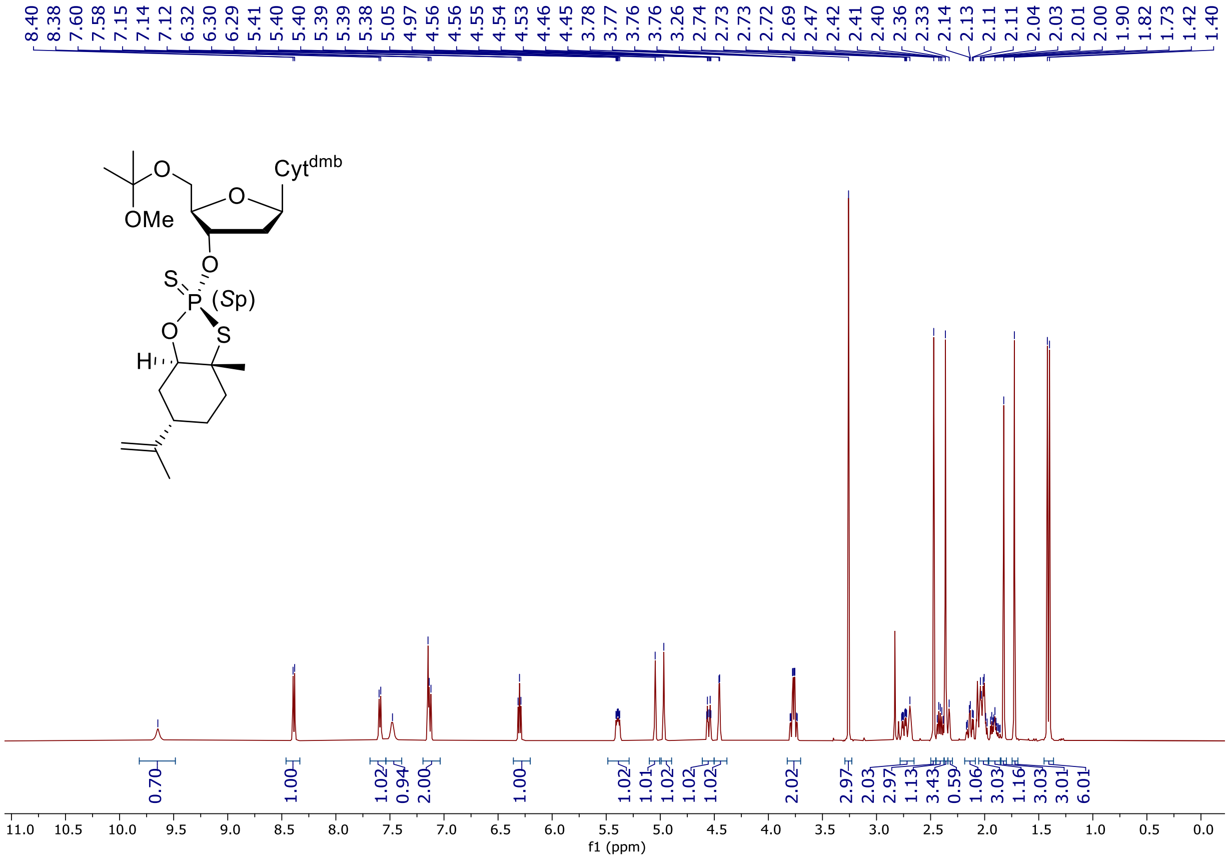

Figure S19. ^{1}H NMR (500 MHz, $(CD_3)_2CO$) spectrum of **2*^{Sp}***.

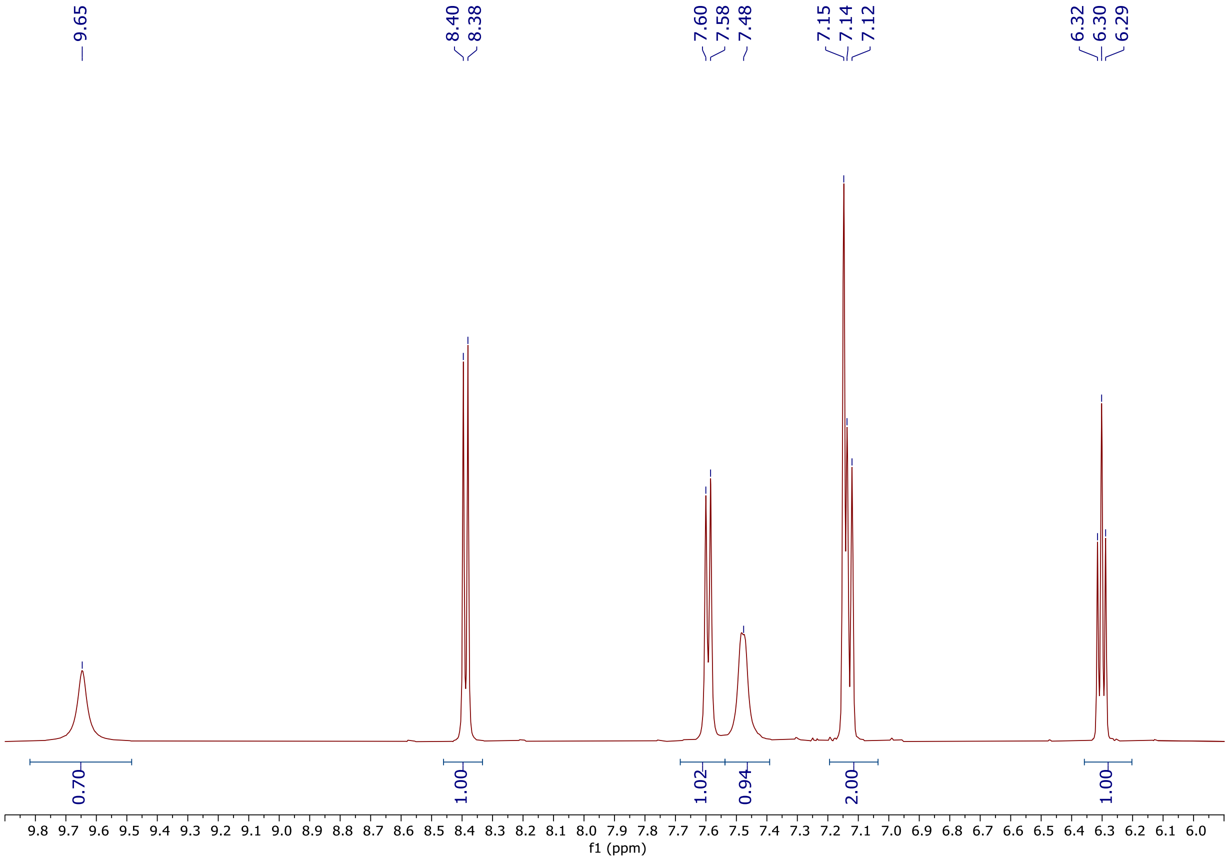

Figure S20. Magnified region (δ_H = 9.9 ppm to 5.9 ppm) of the ^{1}H NMR spectrum of **2*^{Sp}***.

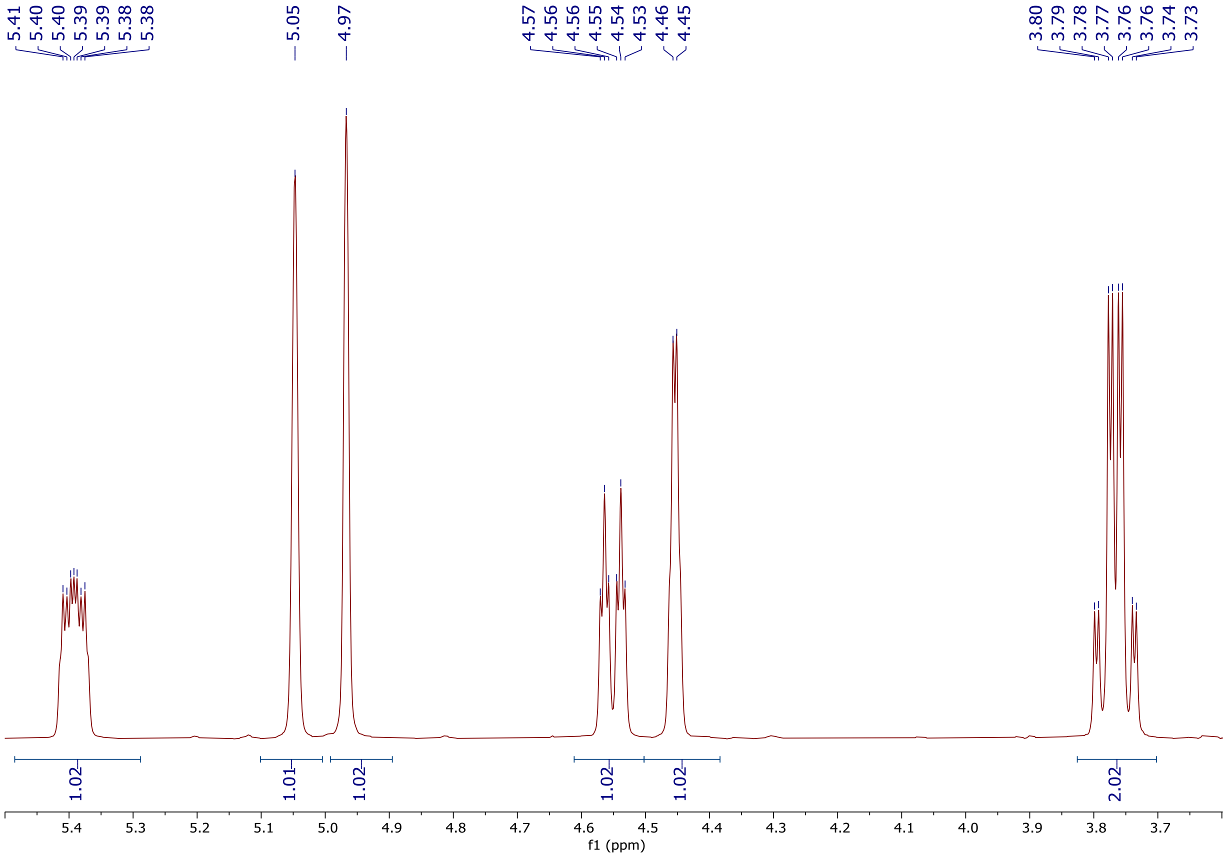

Figure S21. Magnified region (δ_H = 5.5 ppm to 3.6 ppm) of the ^{1}H NMR spectrum of $\mathbf{2}^{\boldsymbol{Sp}}$.

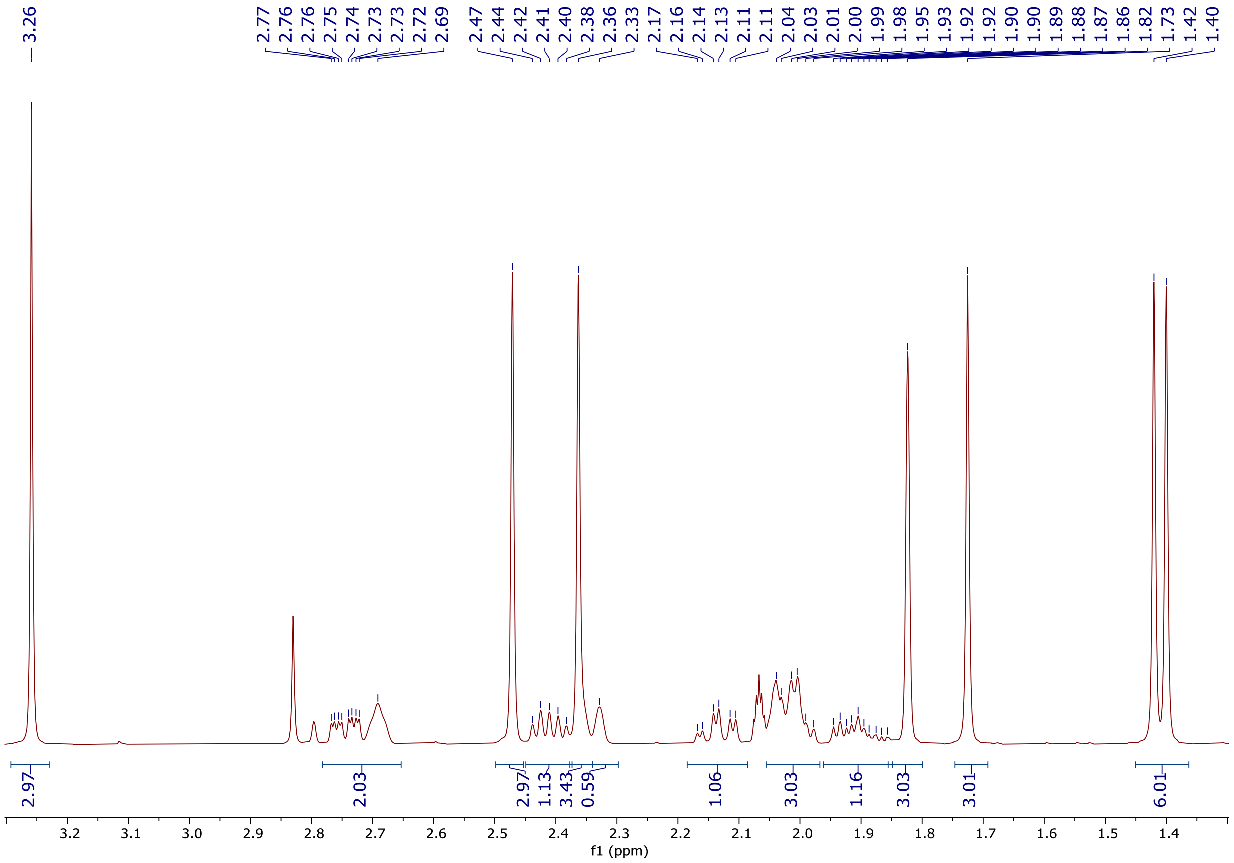

Figure S22. Magnified region (δ_H = 3.3 ppm to 1.3 ppm) of the ^{1}H NMR spectrum of $\mathbf{2}^{\boldsymbol{Sp}}$.

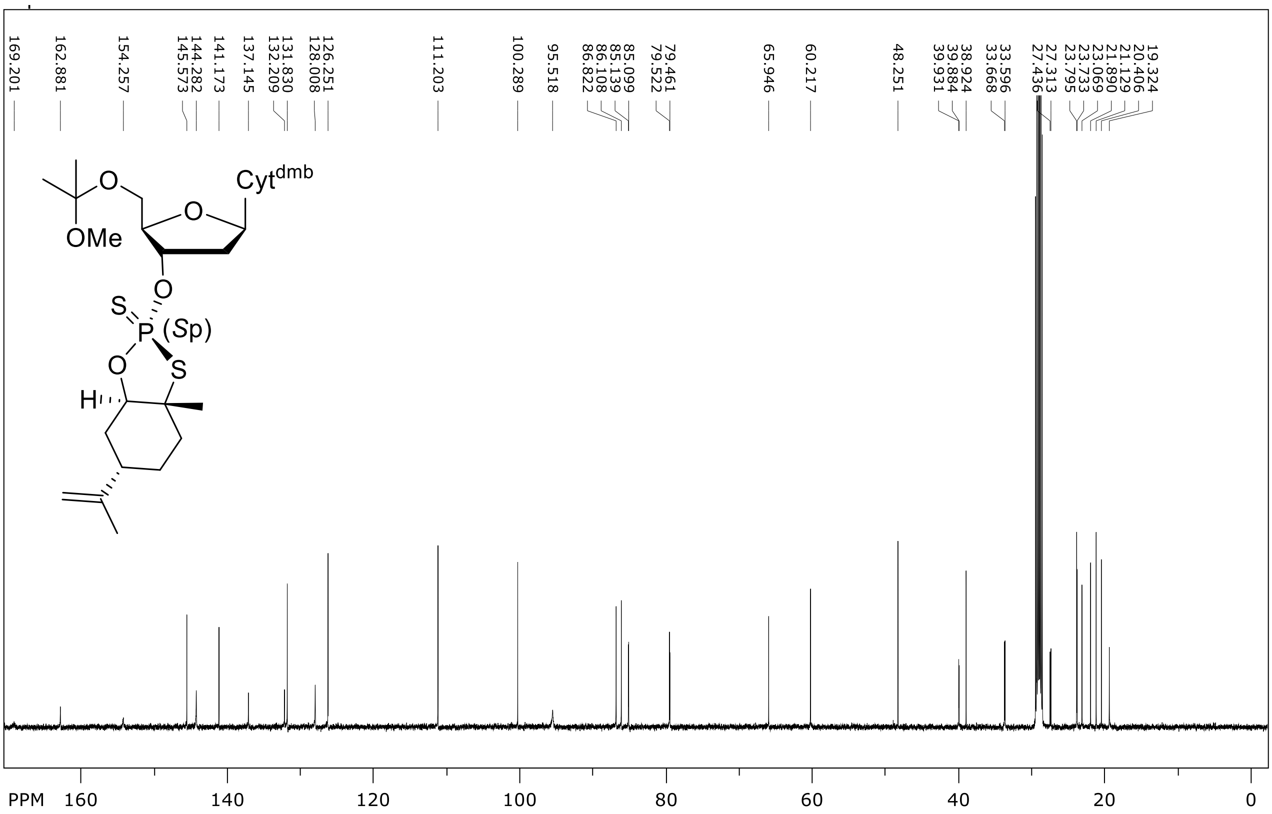

Figure S23. ^{13}C NMR (126 MHz, $(CD_3)_2CO$) spectrum of **2*Sp***.

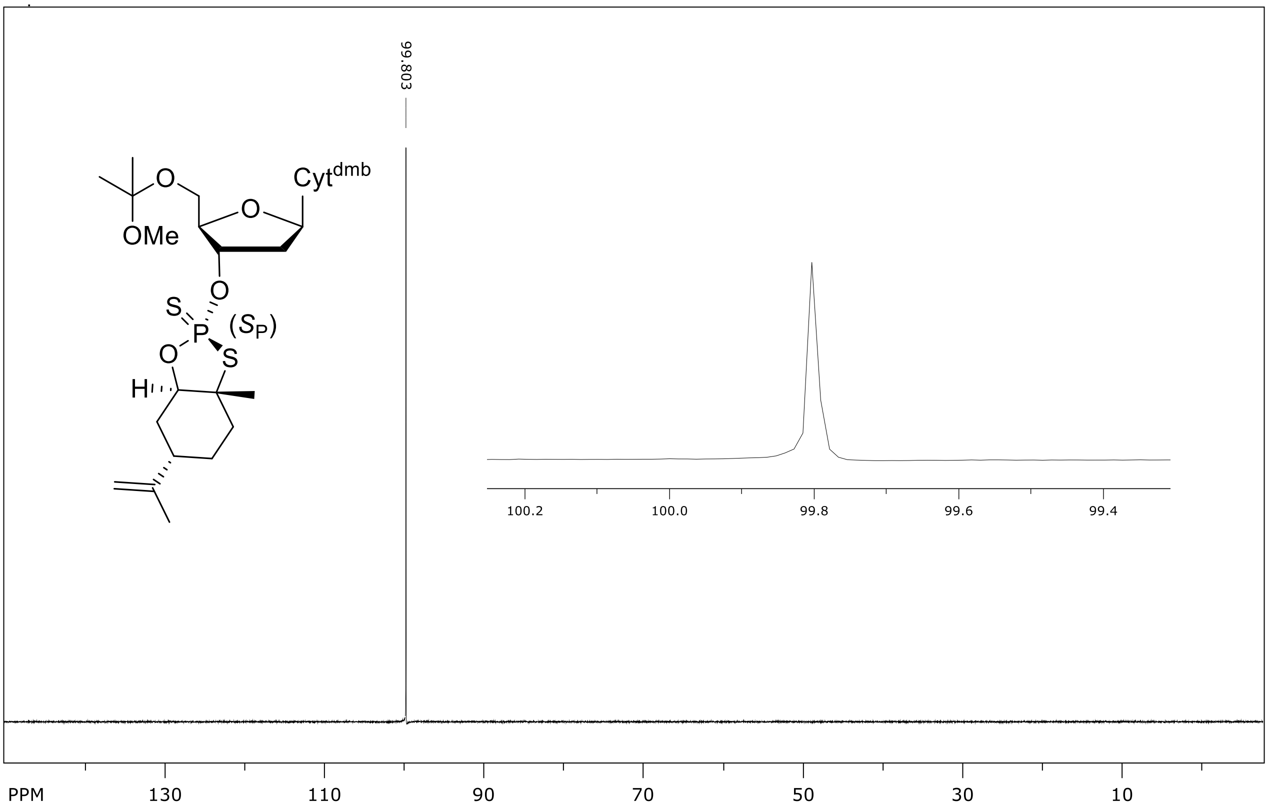

Figure S24. ^{31}P NMR (202 MHz, $(CD_3)_2CO$) spectrum of **2*Sp***

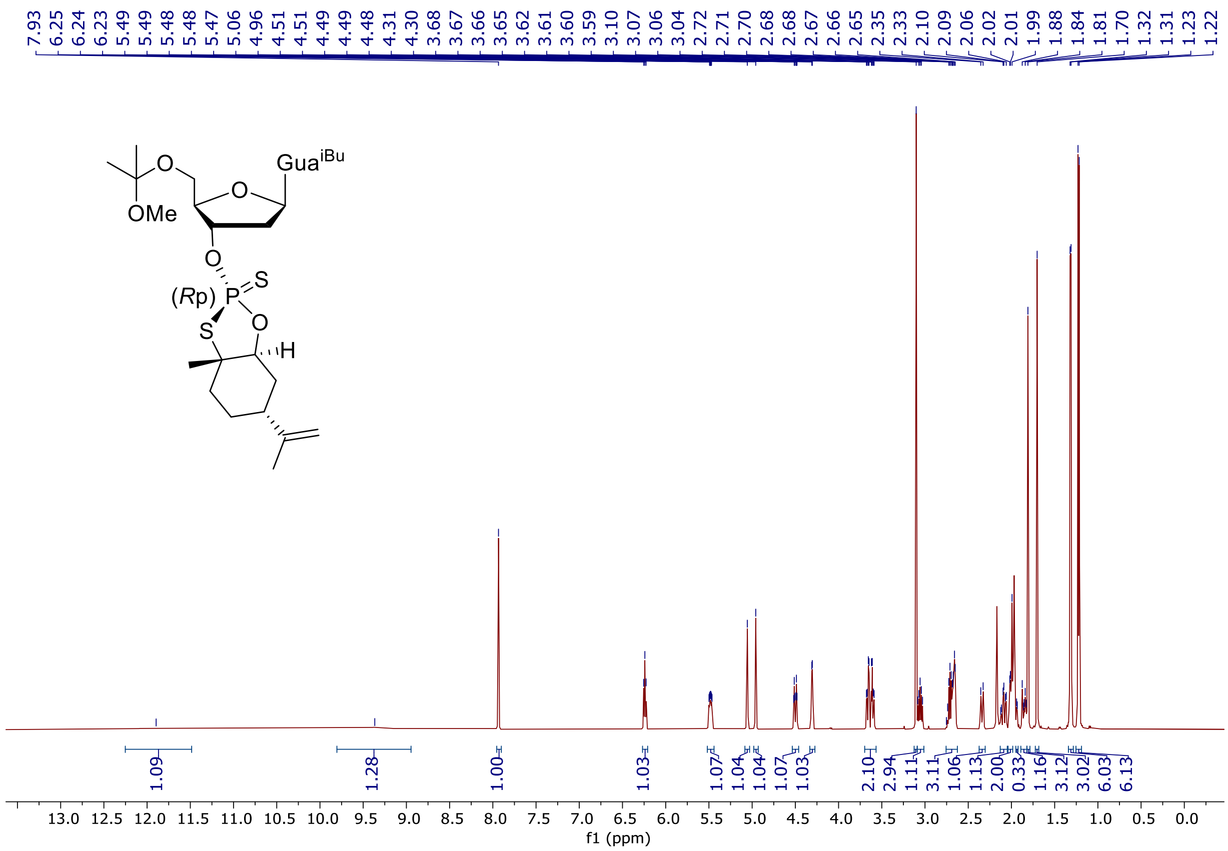

Figure S25. ^{1}H NMR (500 MHz, CD_3CN) spectrum of $\mathbf{3}^{\boldsymbol{Rp}}$.

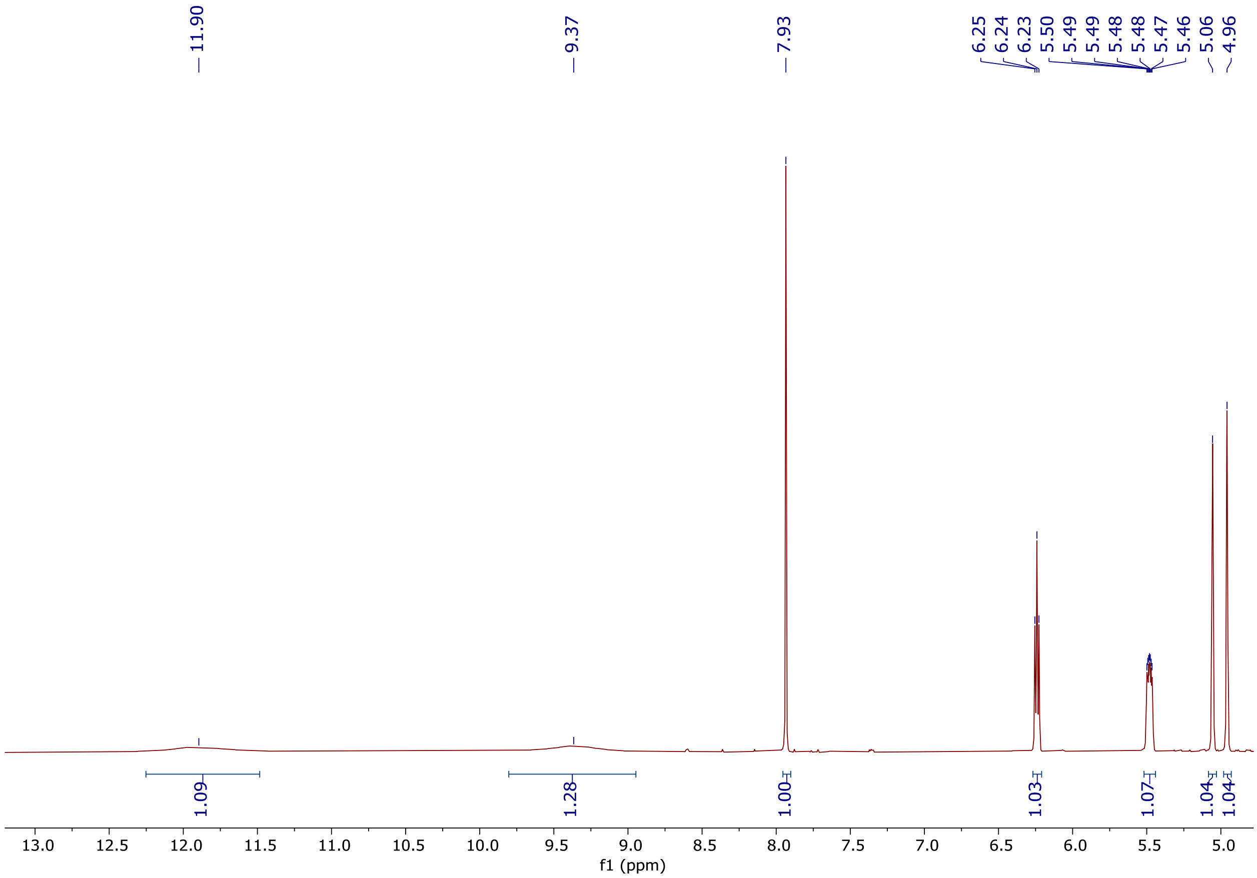

Figure S26. Magnified region (δ_H = 13.2 ppm to 4.8 ppm) of the ^{1}H NMR spectrum of $\mathbf{3}^{\boldsymbol{Rp}}$.

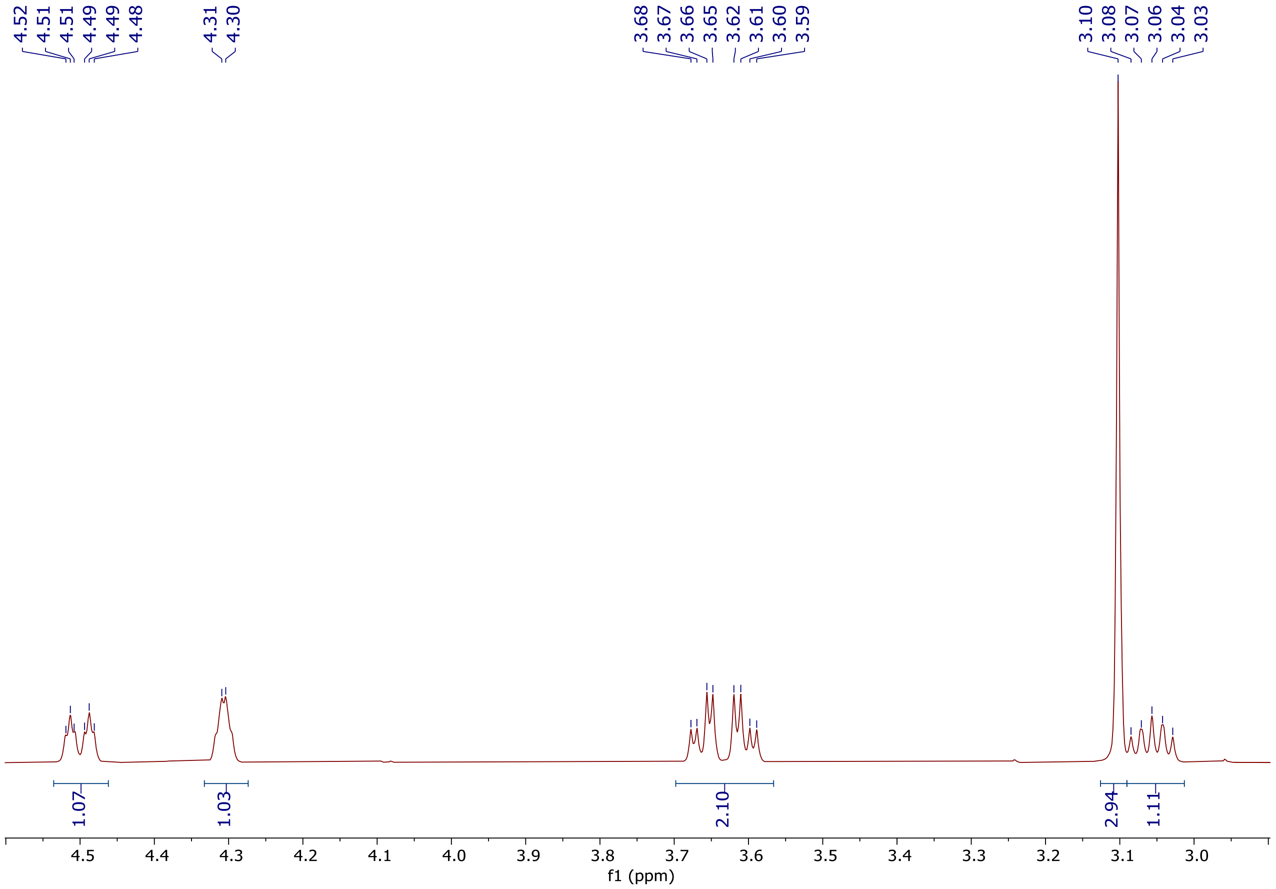

Figure S27. Magnified region (δ_H = 4.6 ppm to 2.9 ppm) of the ^{1}H NMR spectrum of $\mathbf{3}^{\boldsymbol{Rp}}$.

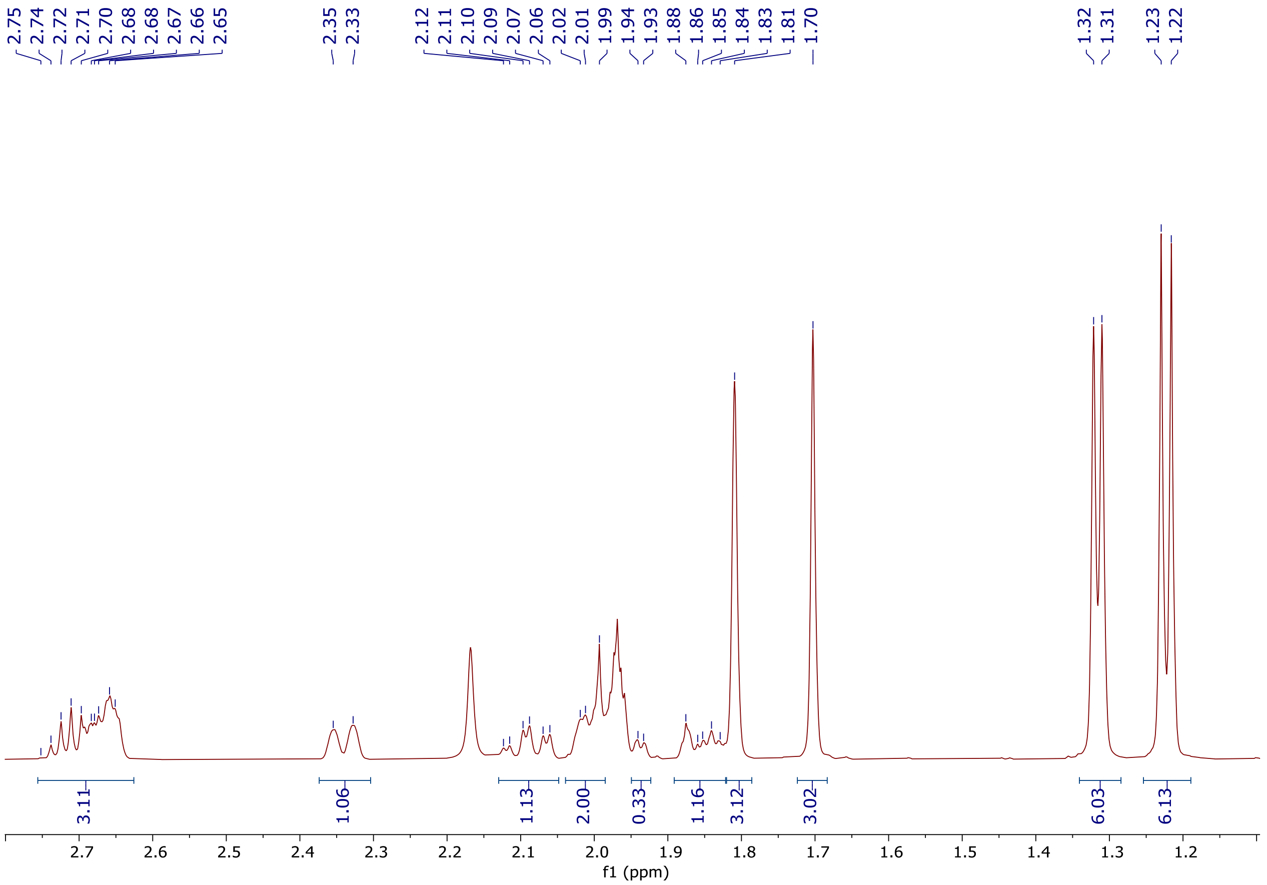

Figure S28. Magnified region (δ_H = 2.8 ppm to 1.1 ppm) of the ^{1}H NMR spectrum of $\mathbf{3}^{\boldsymbol{Rp}}$.

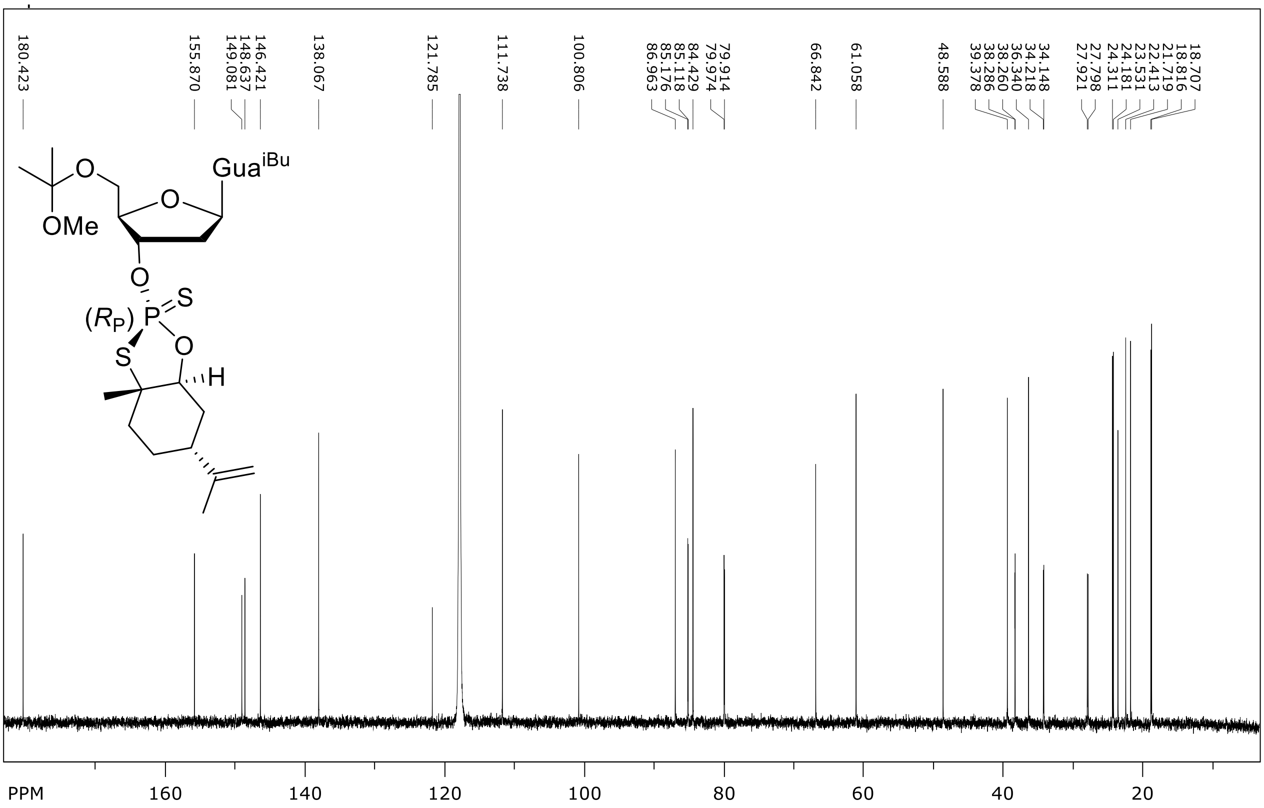

Figure S29. ^{13}C NMR (126 MHz, CD$_3$CN) spectrum of **3*^{Rp}***.

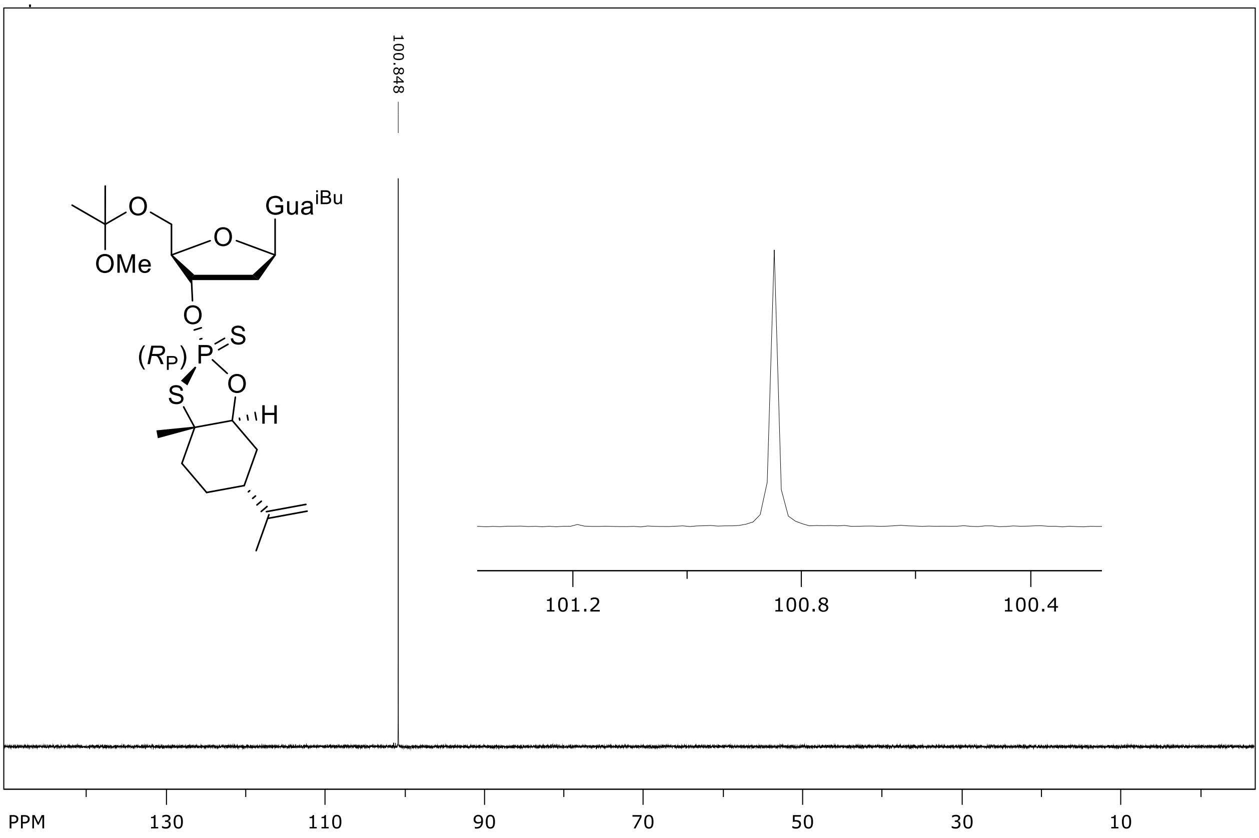

Figure S30. ^{31}P NMR (202 MHz, CD$_3$CN) spectrum of **3*^{Rp}***

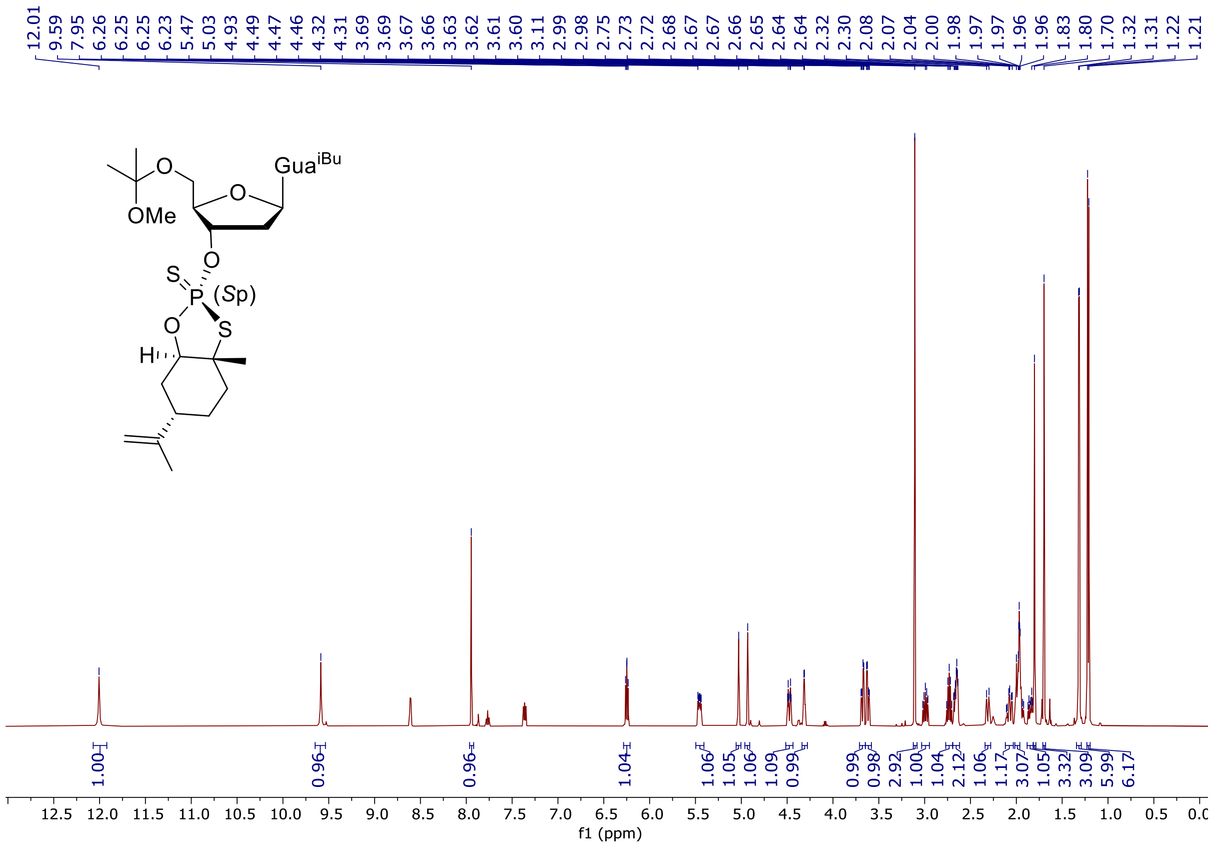

Figure S31. ^{1}H NMR (500 MHz, CD_3CN) spectrum of **3*Sp***.

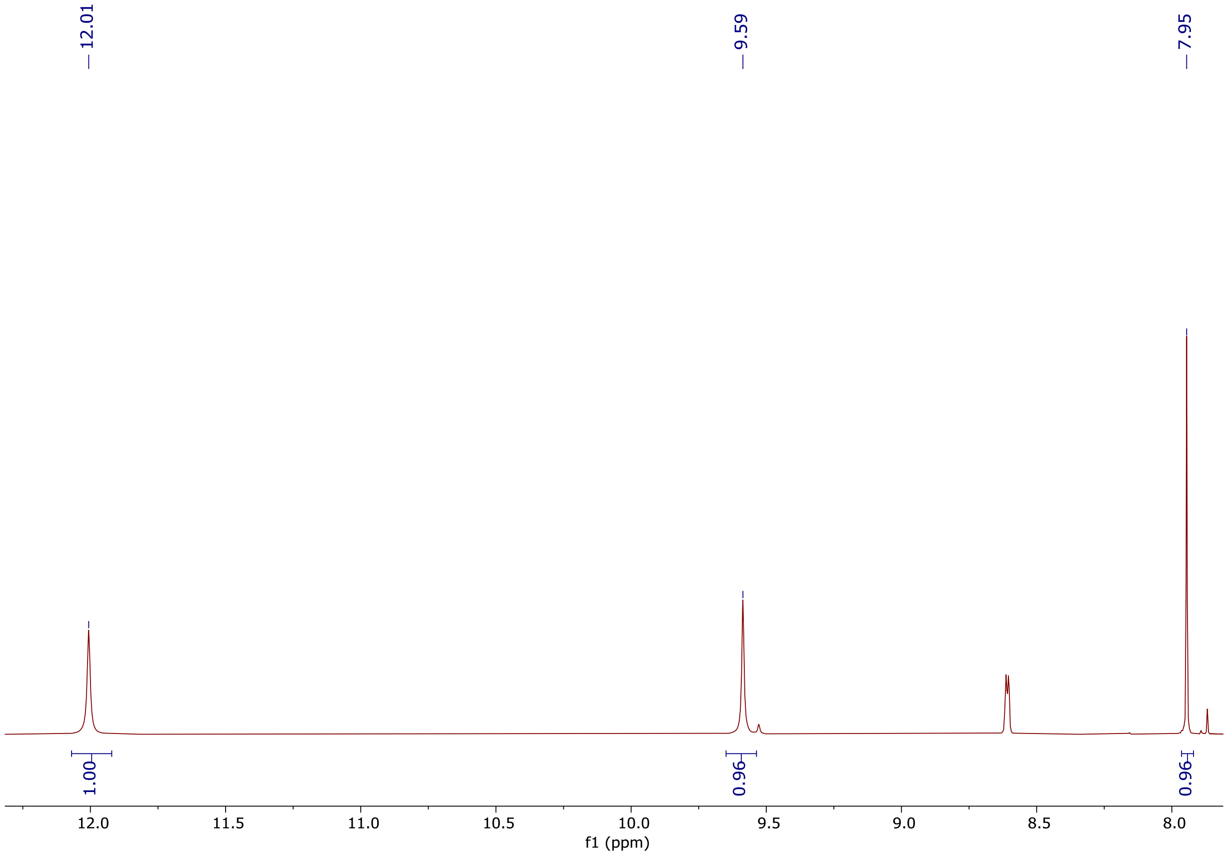

Figure S32. Magnified region (δ_H = 12.3 ppm to 7.8 ppm) of the ^{1}H NMR spectrum of **3*Sp***.

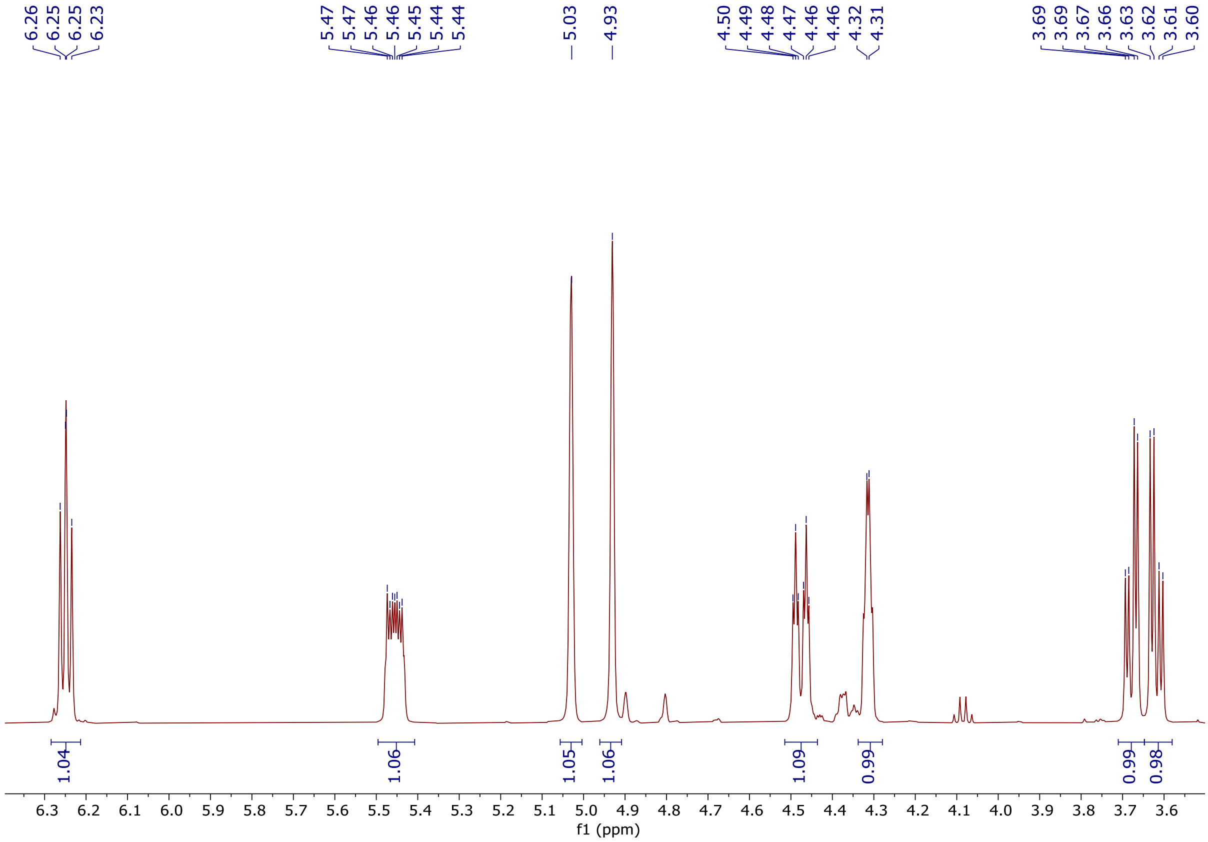

Figure S33. Magnified region (δ_H = 6.4 ppm to 3.5 ppm) of the ^{1}H NMR spectrum of $\mathbf{3}^{Sp}$.

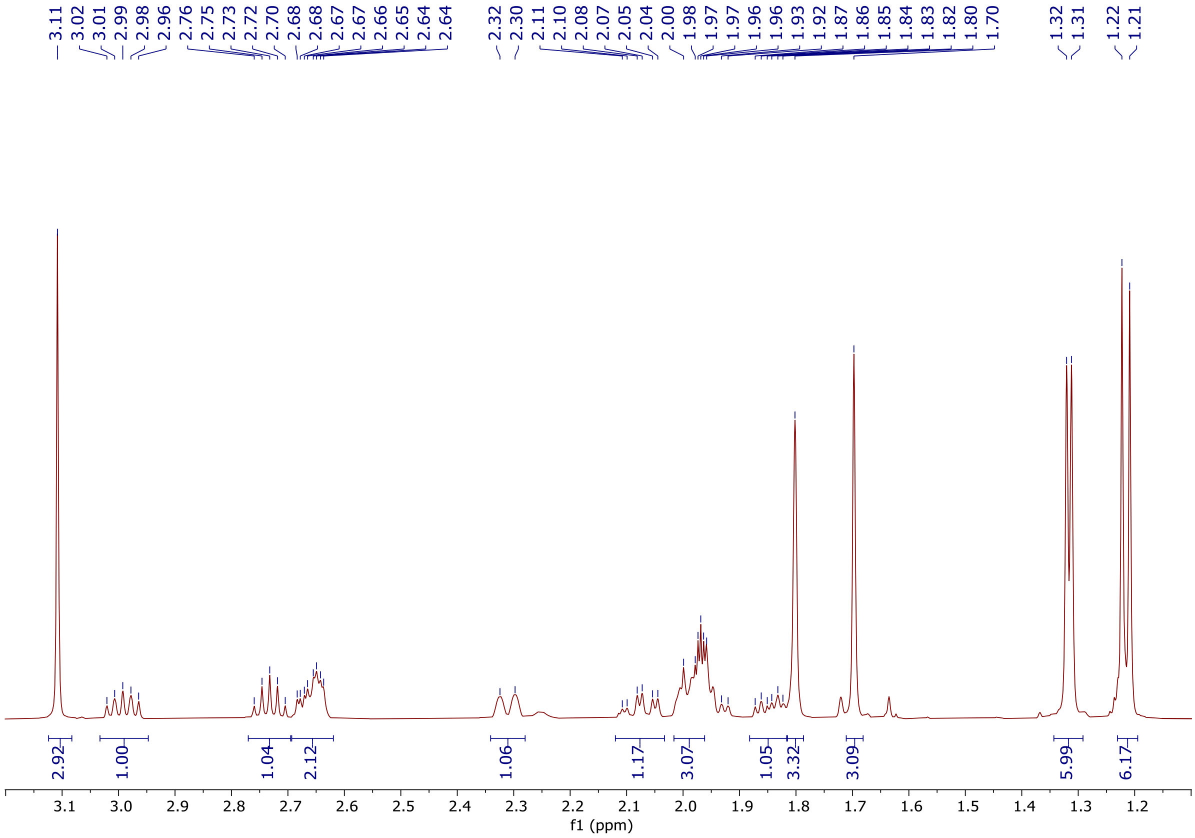

Figure S34. Magnified region (δ_H = 3.2 ppm to 1.1 ppm) of the ^{1}H NMR spectrum of $\mathbf{3}^{Sp}$.

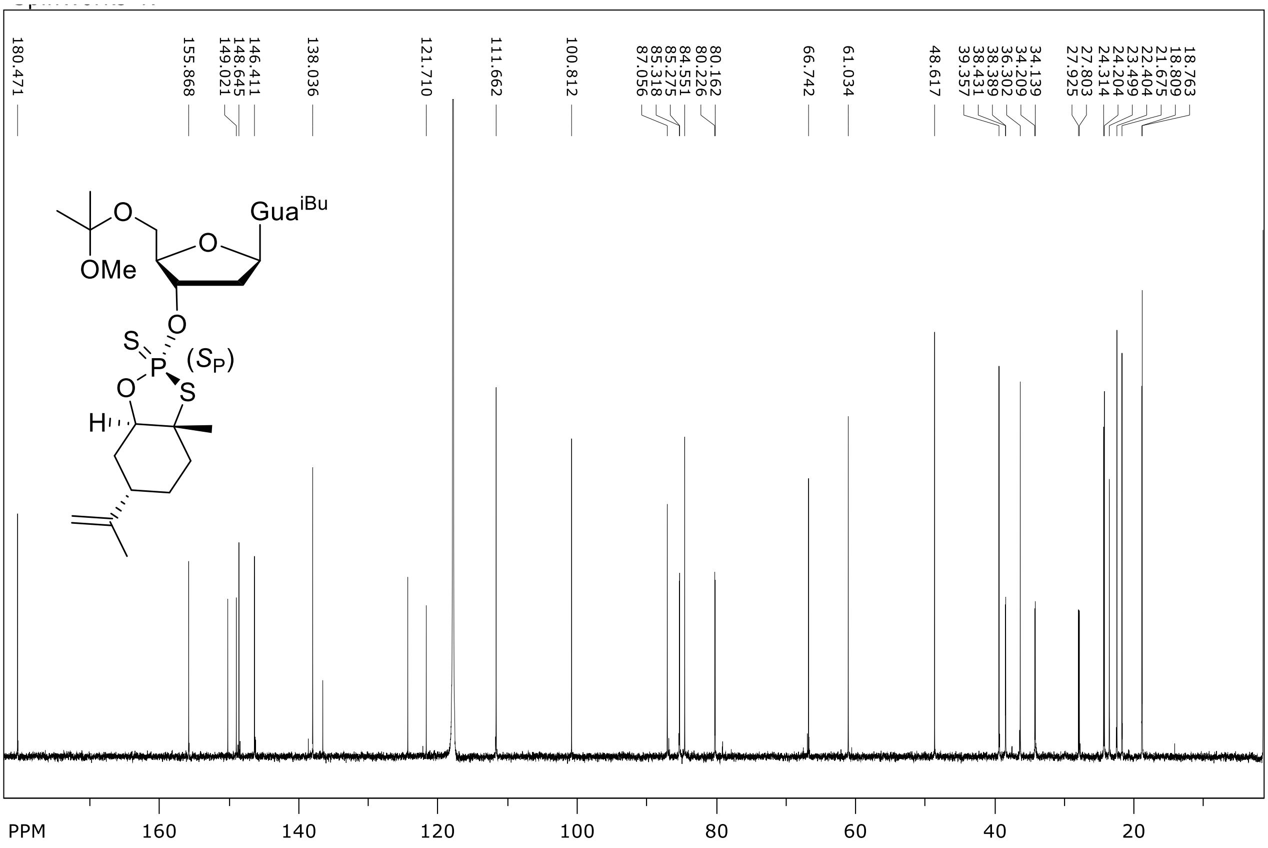

Figure S35. ^{13}C NMR (126 MHz, CD_3CN) spectrum of **3^{Sp}**.

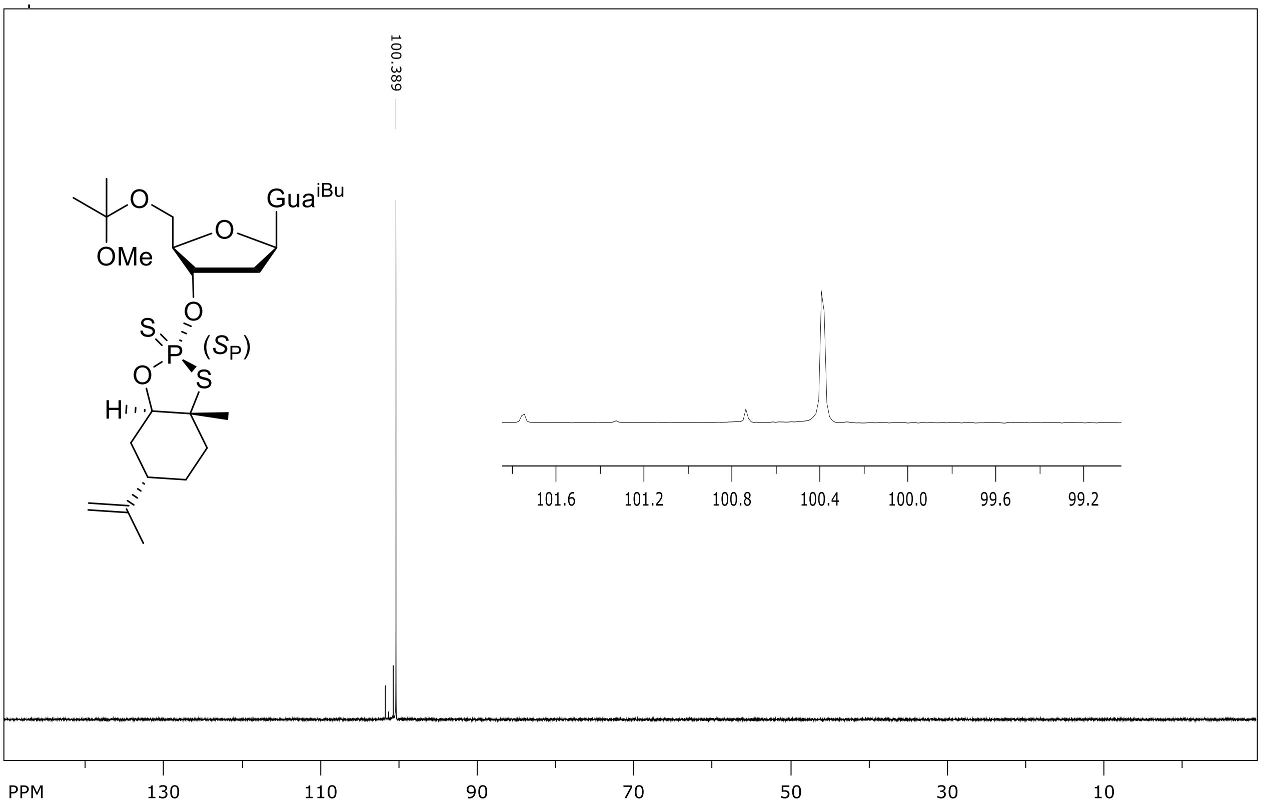

Figure S36. ^{31}P NMR (202 MHz, CD_3CN) spectrum of **3^{Sp}**

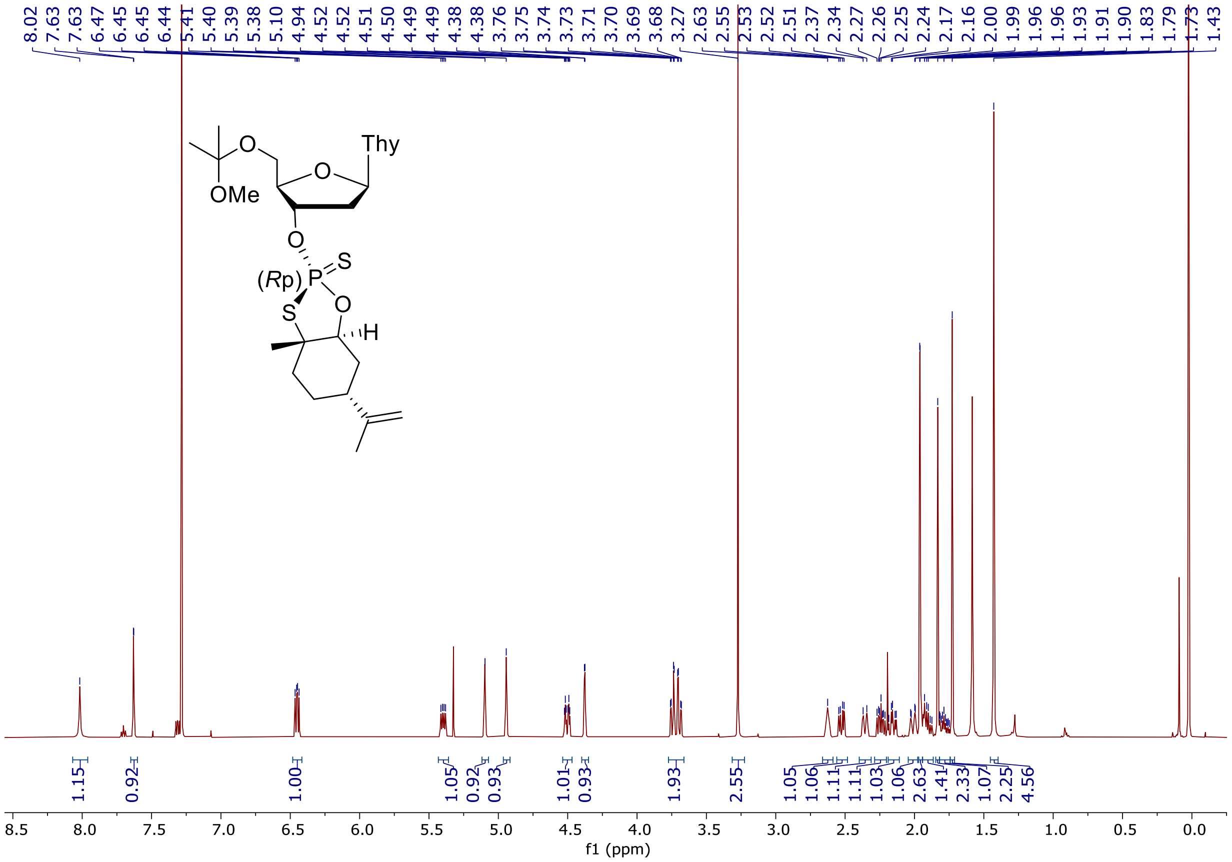

Figure S37. ^{1}H NMR (500 MHz, CD_3CN) spectrum of **4*Rp***.

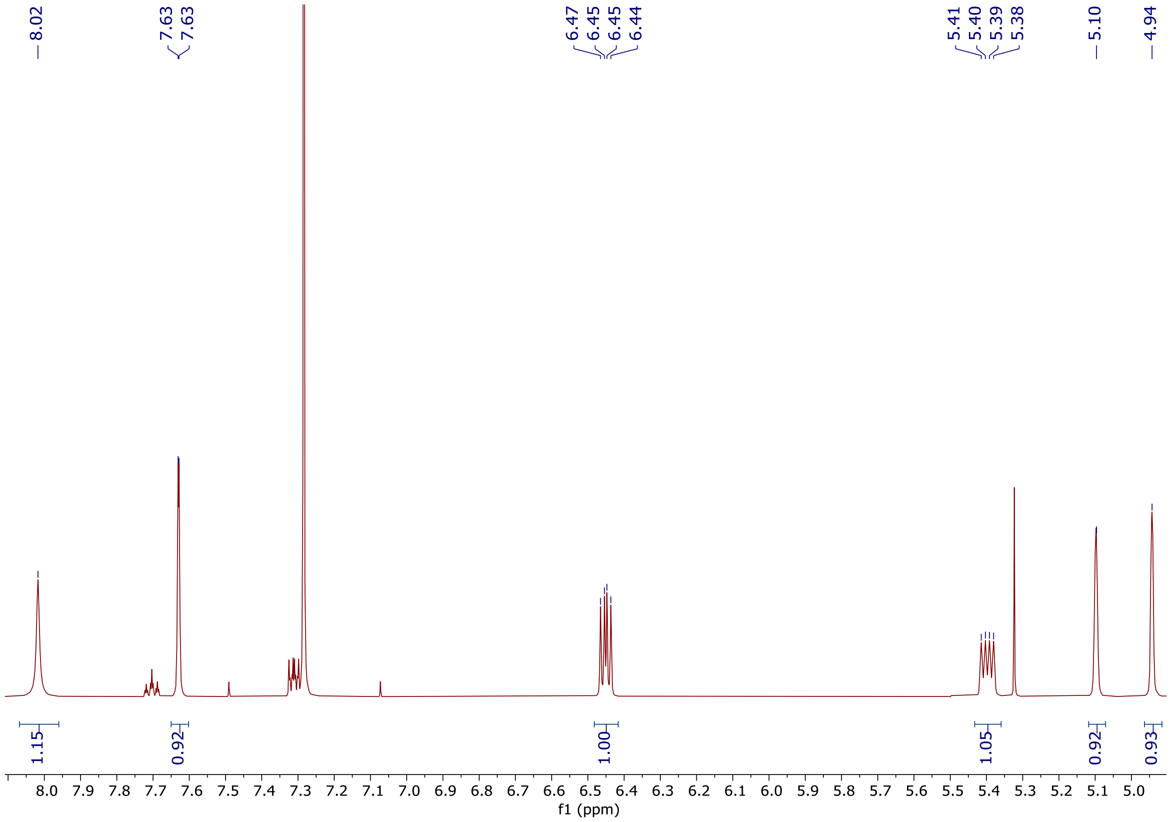

Figure S38. Magnified region (δ_H = 8.1 ppm to 4.9 ppm) of the ^{1}H NMR spectrum of **4*Rp***.

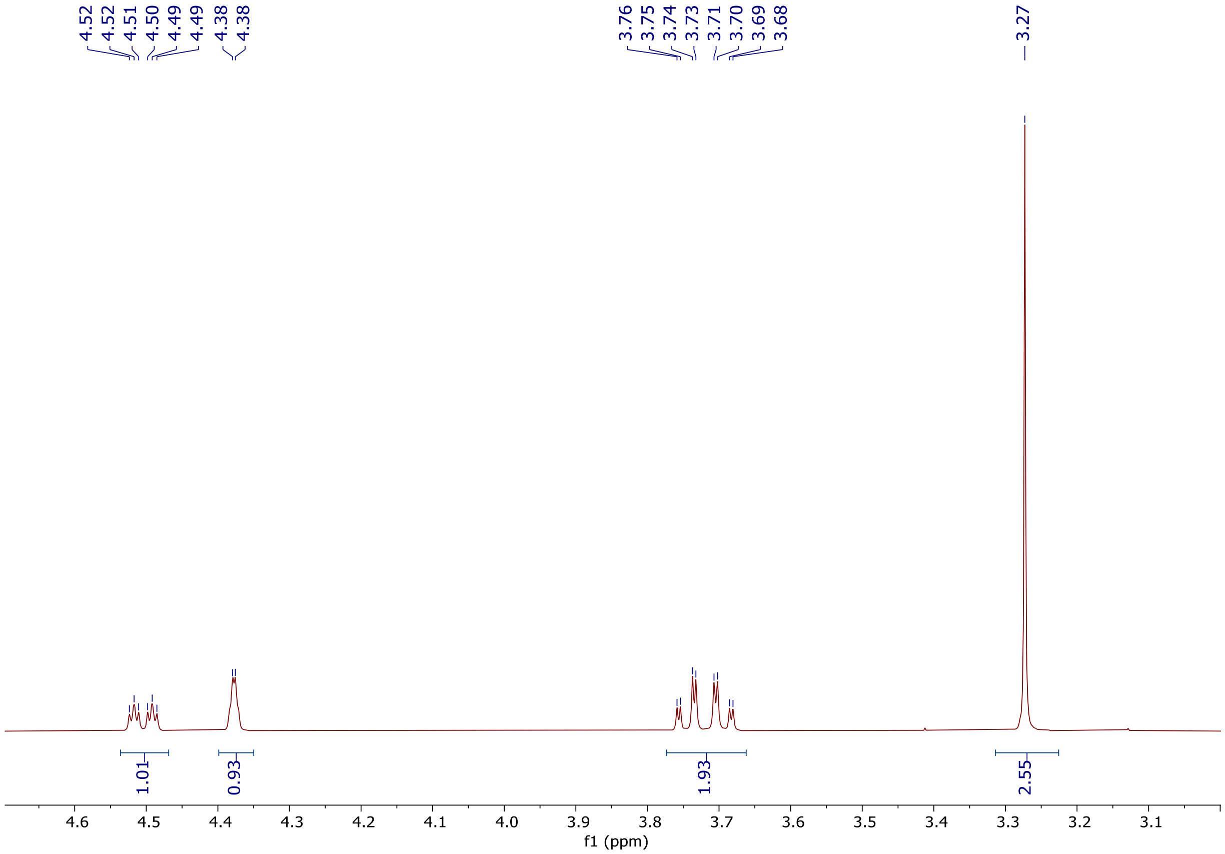

Figure S39. Magnified region (δ_H = 4.7 ppm to 3.0 ppm) of the ^{1}H NMR spectrum of $\mathbf{4}^{\boldsymbol{Rp}}$.

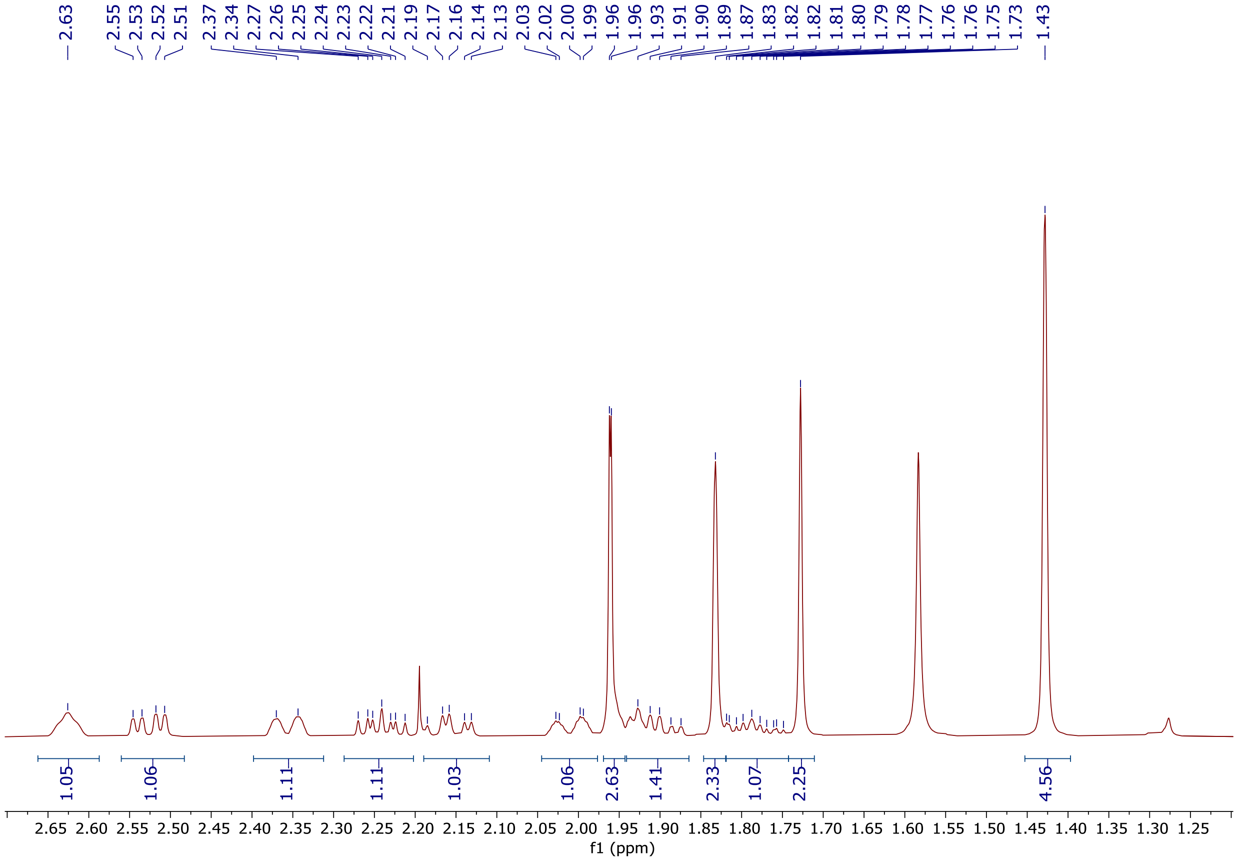

Figure S40. Magnified region (δ_H = 2.7 ppm to 1.2 ppm) of the ^{1}H NMR spectrum of $\mathbf{4}^{\boldsymbol{Rp}}$.

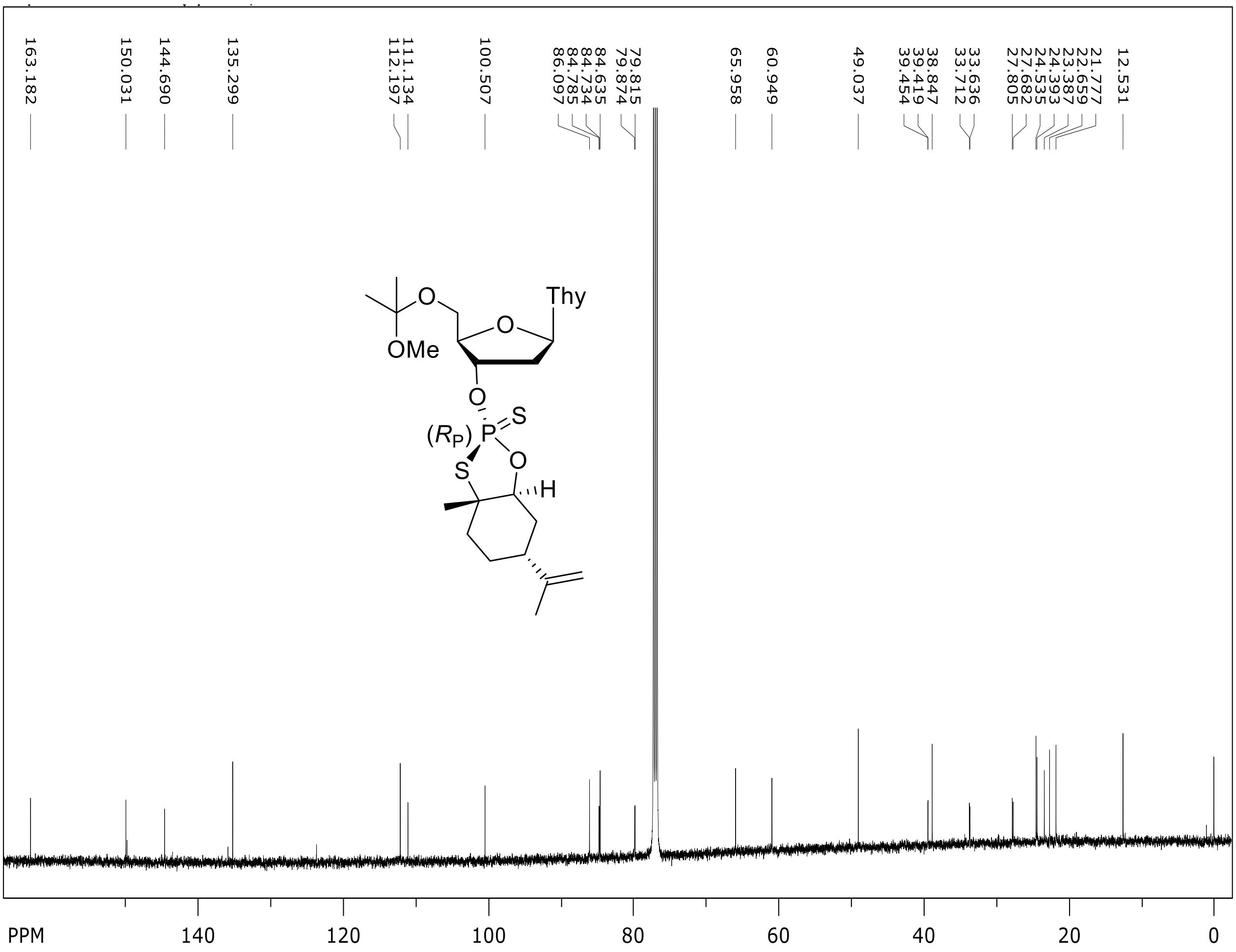

Figure S41. ^{13}C NMR (126 MHz, CD_3CN) spectrum of $\mathbf{4}^{\boldsymbol{Rp}}$.

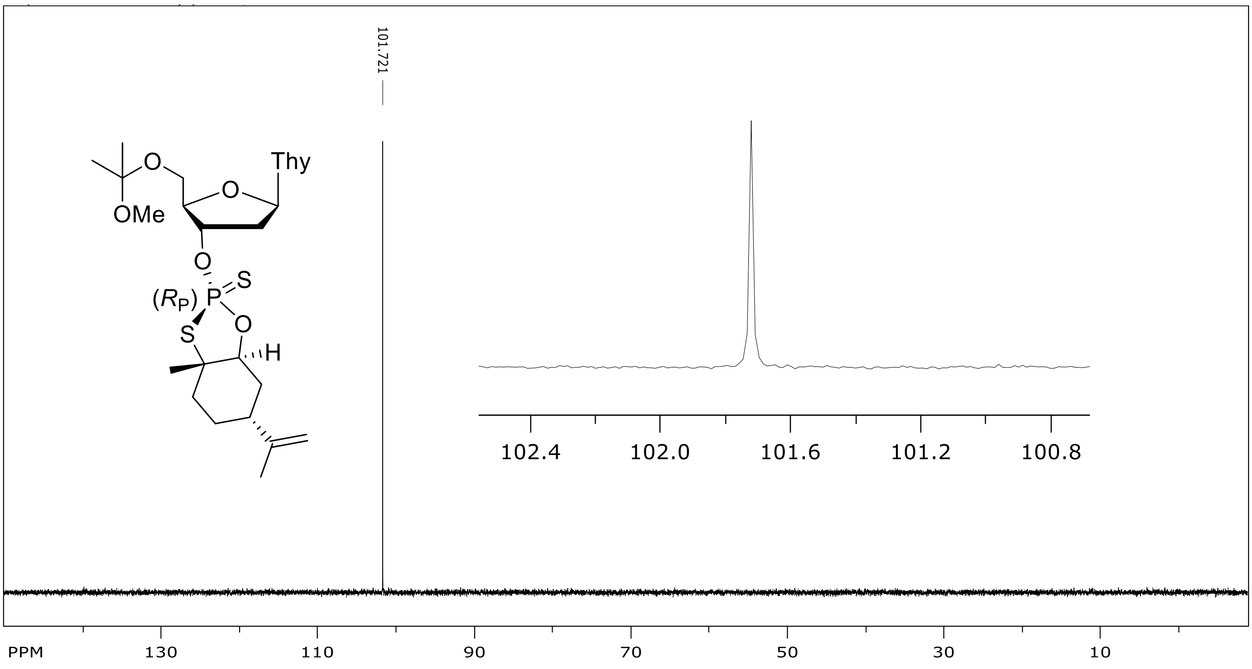

Figure S42. ^{31}P NMR (202 MHz, CD_3CN) spectrum of $\mathbf{4}^{\boldsymbol{Rp}}$

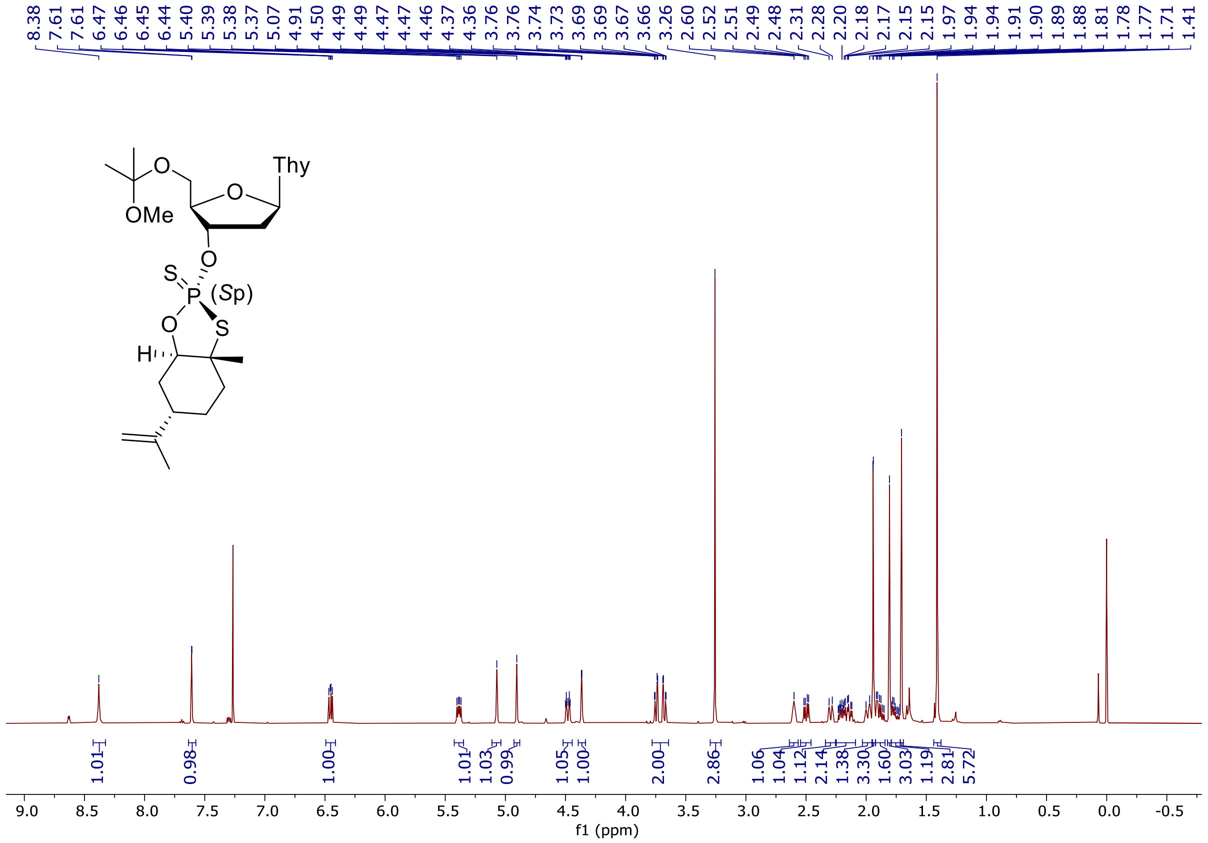

Figure S43. ^{1}H NMR (500 MHz, CD^{3}CN) spectrum of **4**^{Sp}.

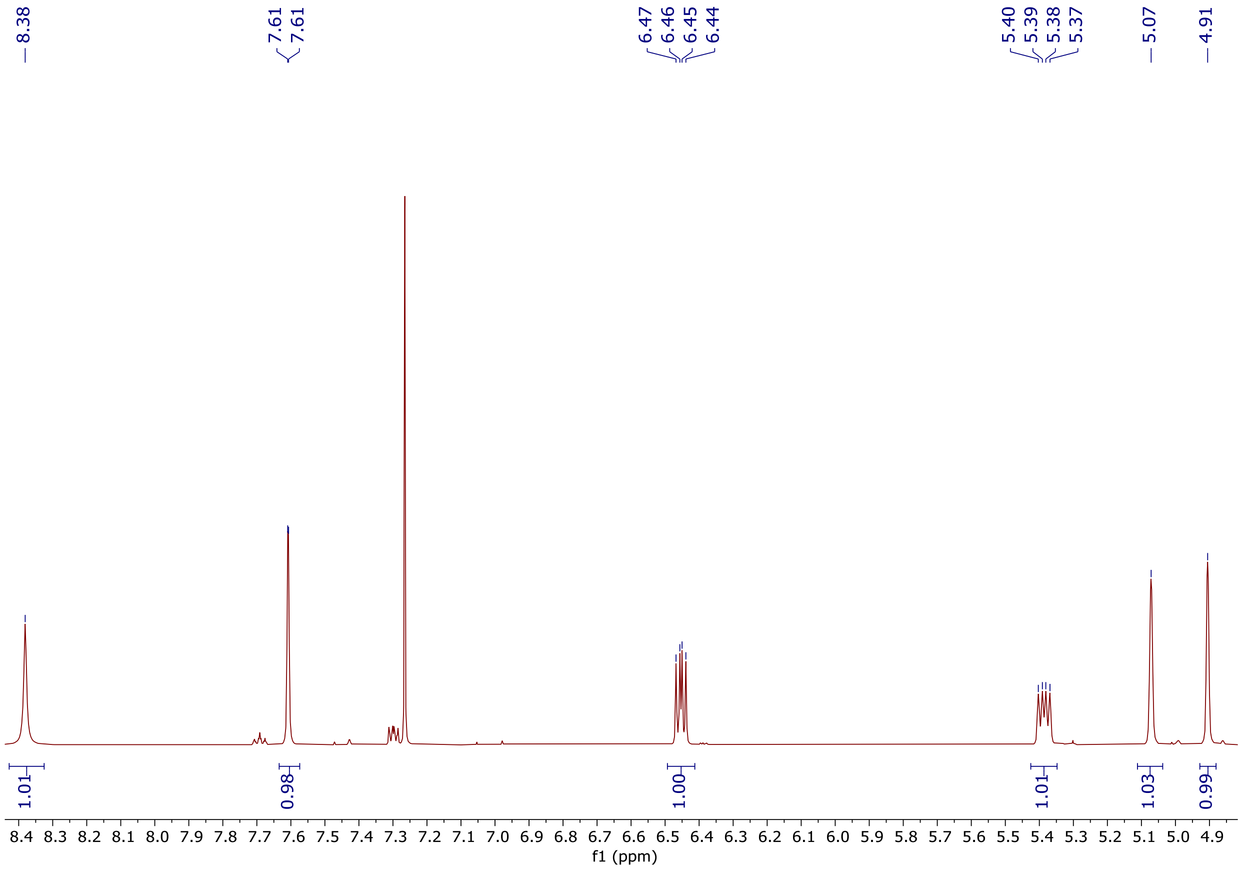

Figure S44. Magnified region (δ_H = 8.4 ppm to 4.8 ppm) of the ^{1}H NMR spectrum of **4**^{Sp}.

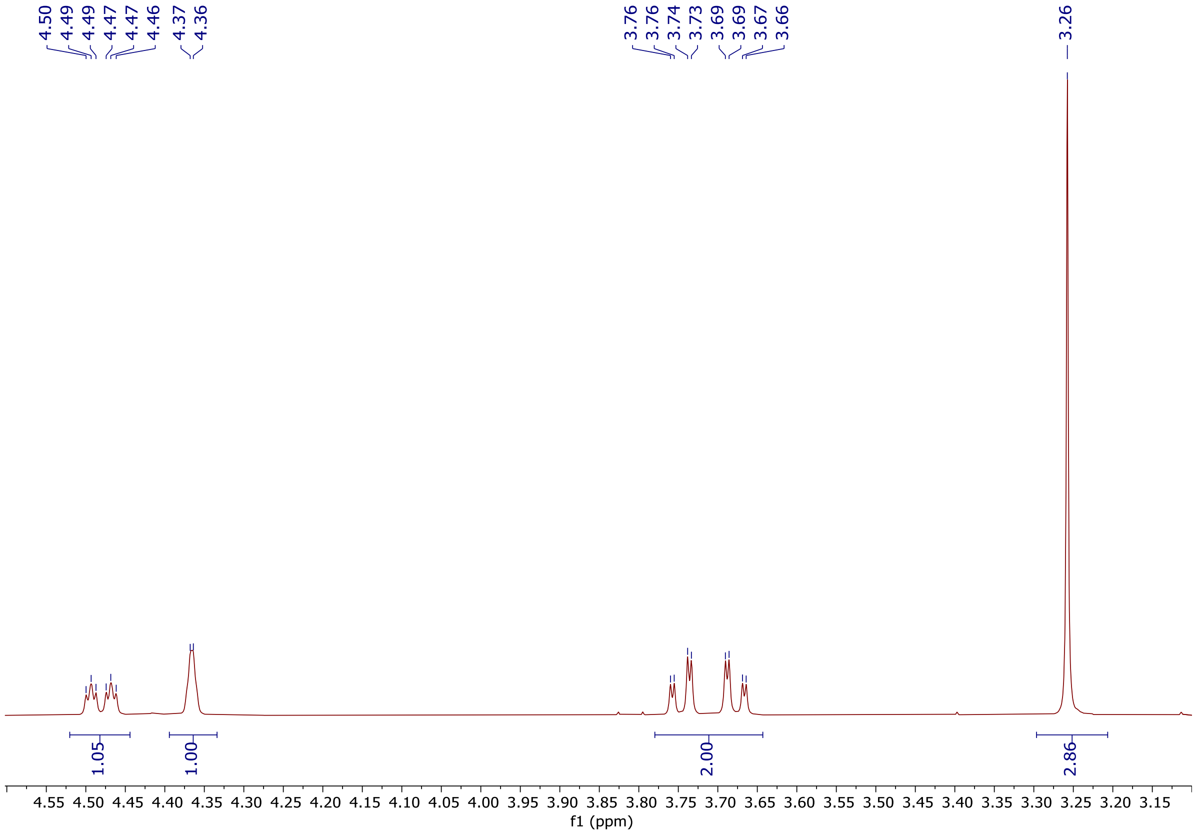

Figure S45. Magnified region (δ_H = 4.6 ppm to 3.1 ppm) of the ^{1}H NMR spectrum of $\mathbf{4}^{\boldsymbol{Sp}}$.

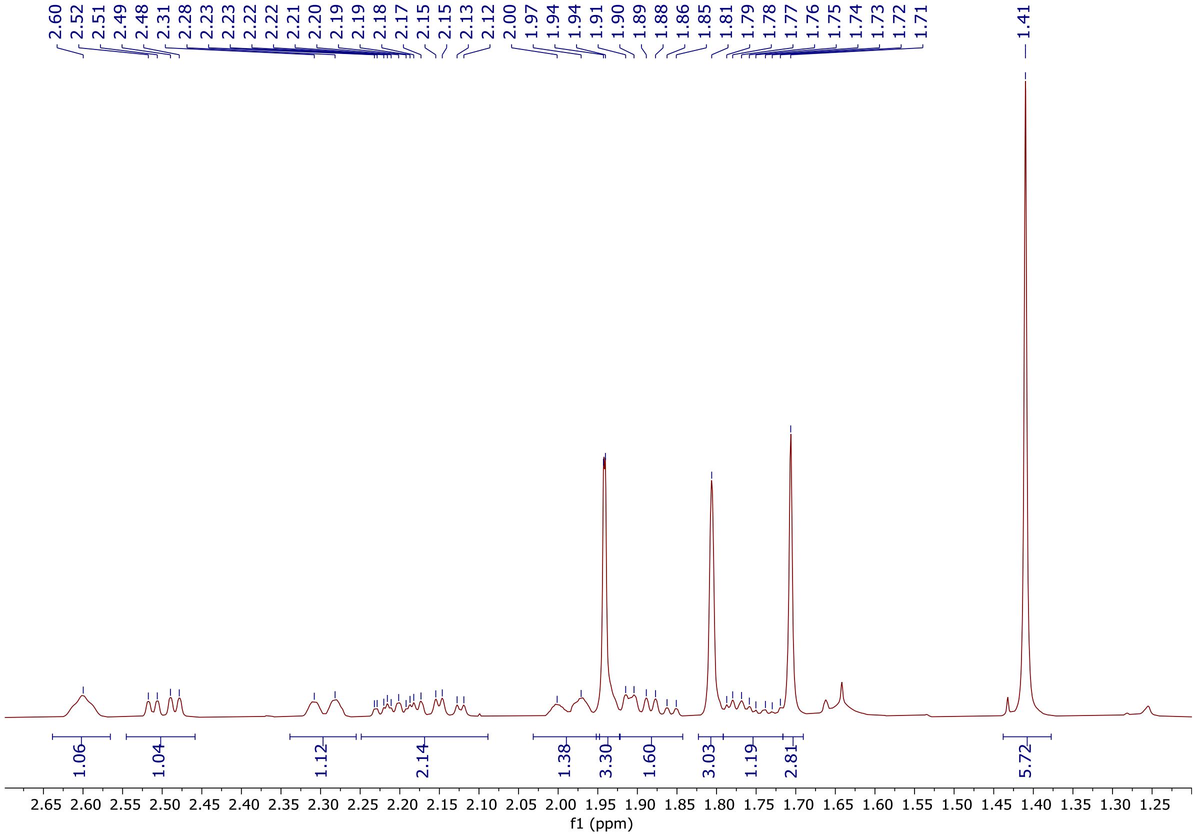

Figure S46. Magnified region (δ_H = 2.7 ppm to 1.2 ppm) of the ^{1}H NMR spectrum of $\mathbf{4}^{\boldsymbol{Sp}}$.

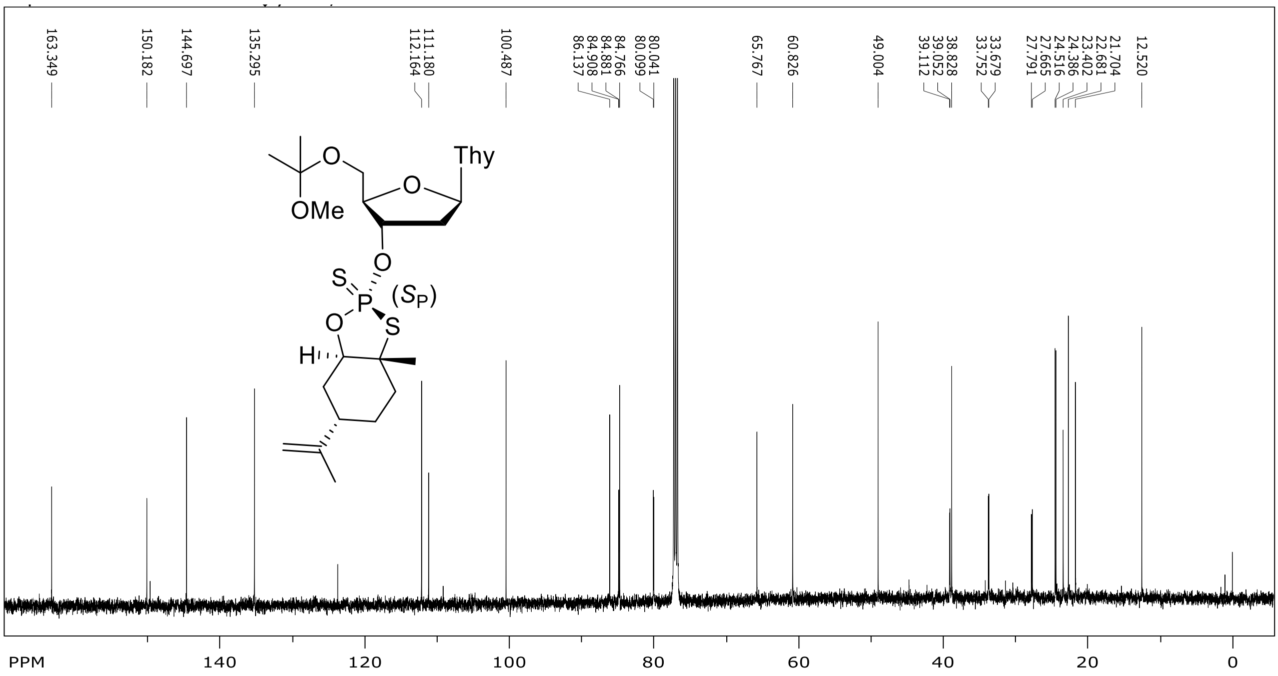

Figure S47. ^{13}C NMR (126 MHz, CD_3CN) spectrum of **4*Sp***.

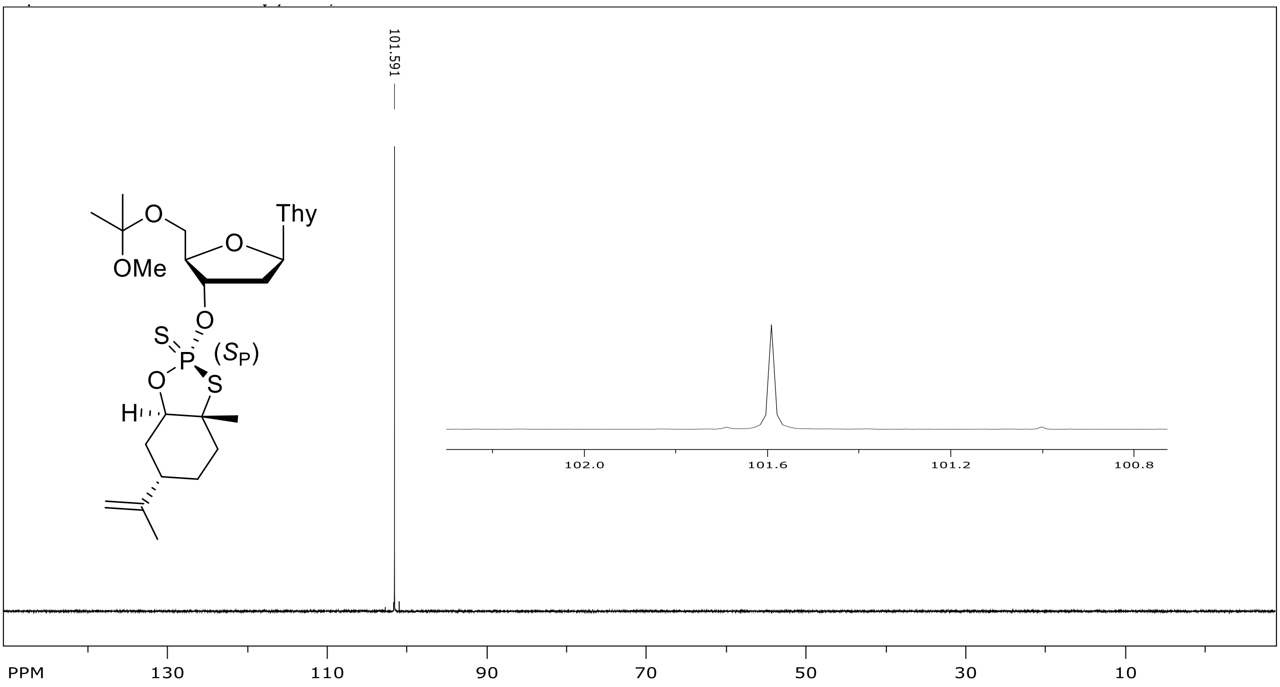

Figure S48. ^{31}P NMR (202 MHz, CD_3CN) spectrum of **4*Sp***

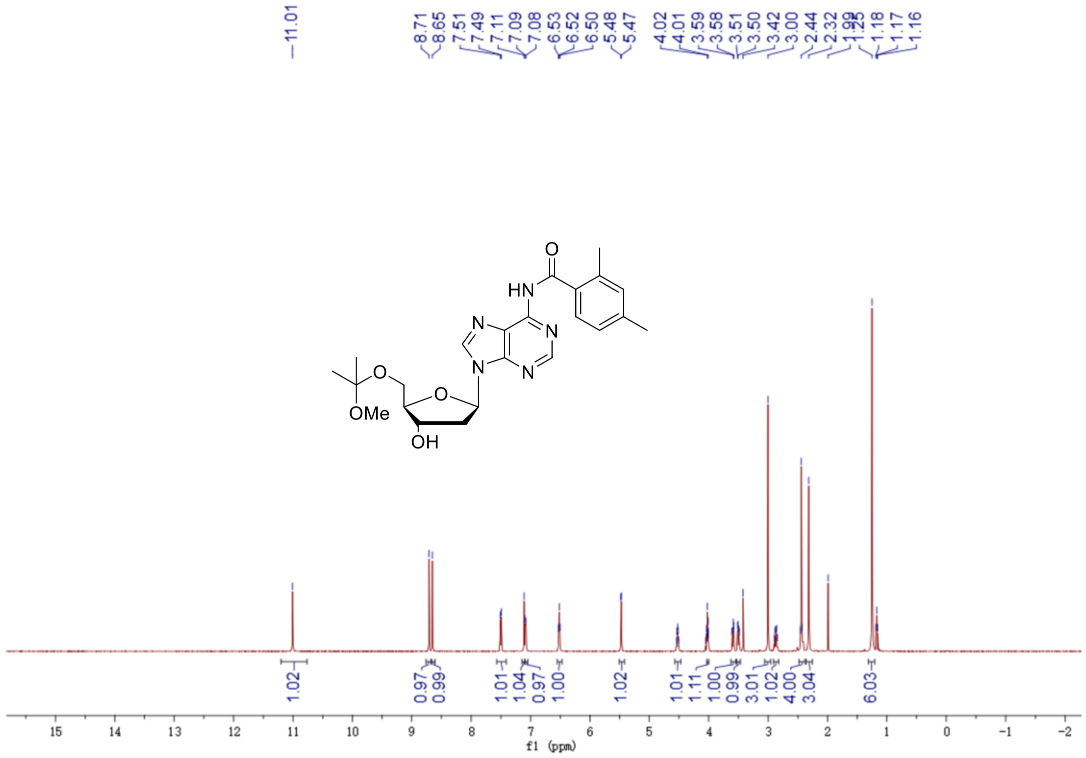

Figure S49. ^{1}H NMR (500 MHz, DMSO-d_6) spectrum of **9**.

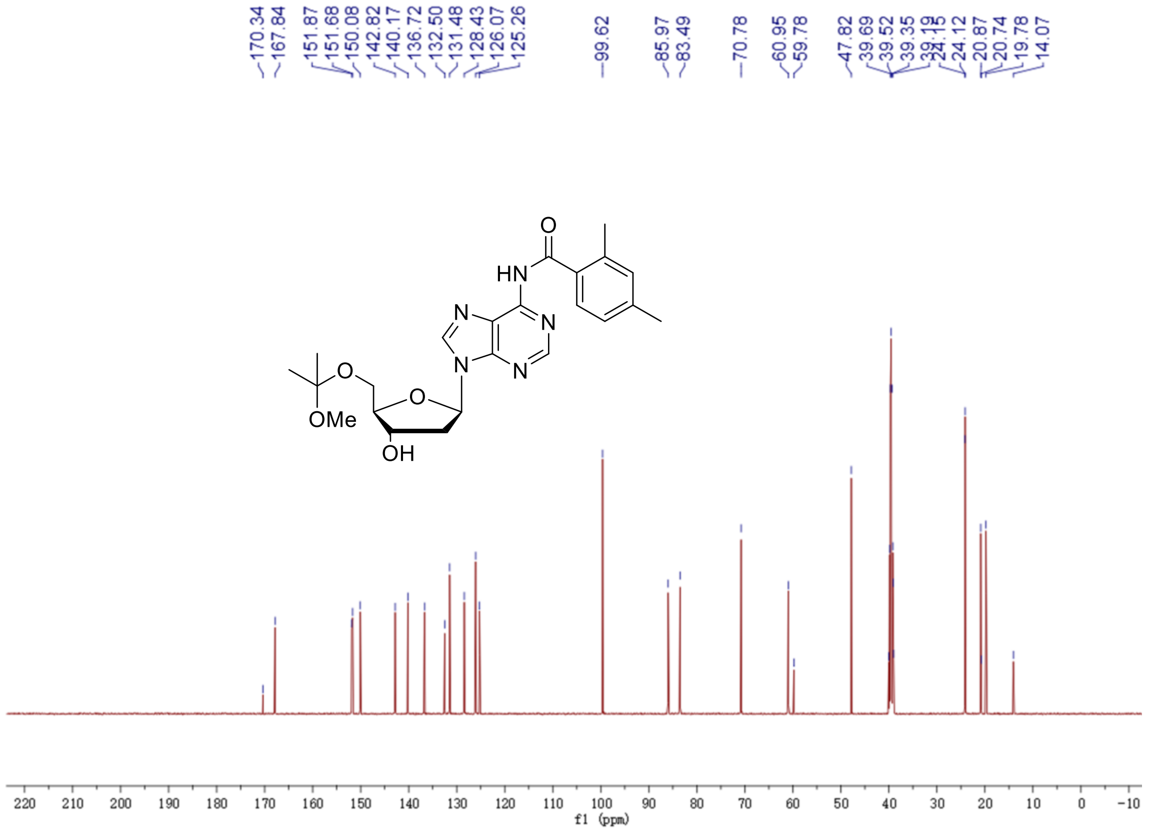

Figure S50. ^{13}C NMR (126 MHz, DMSO-d_6) spectrum of **9**. (DMSO and EtOAc solvent peaks: 170.3, 59.8, 39.7, 39.4, 39.2 and 14.07).

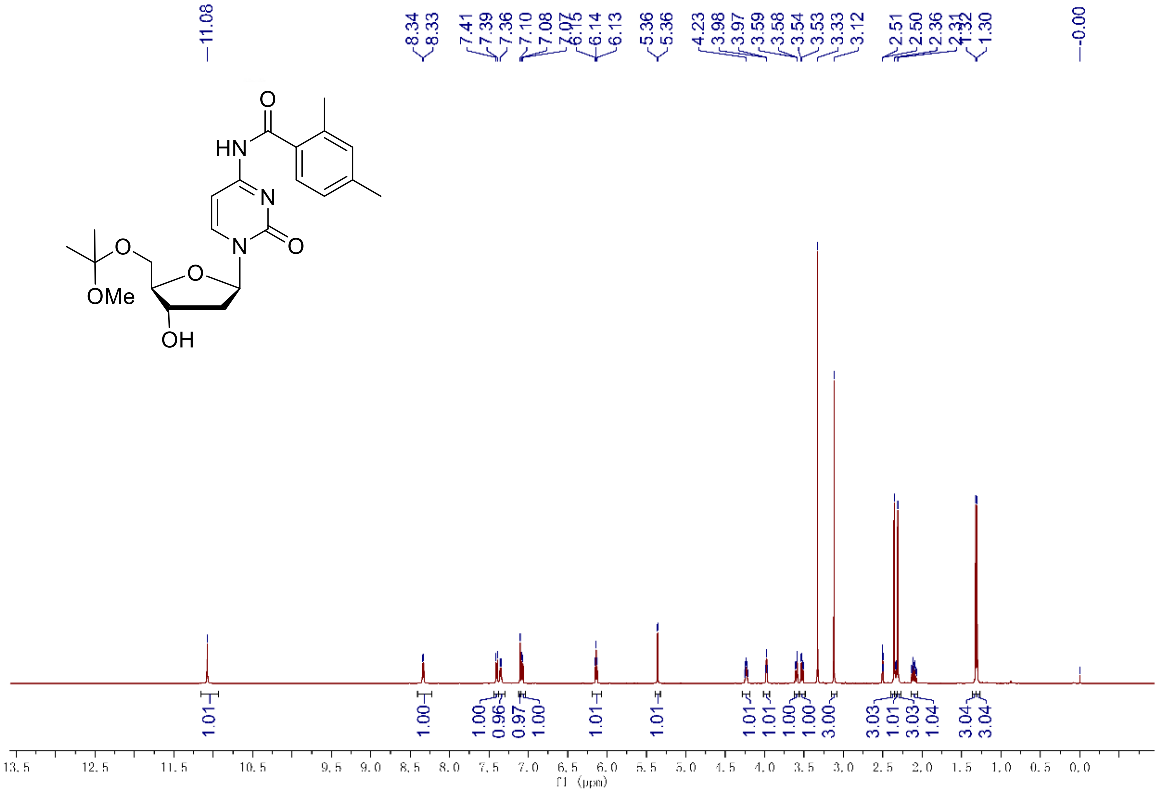

Figure S51. ^{1}H NMR (500 MHz, DMSO-d_6) spectrum of **10**.

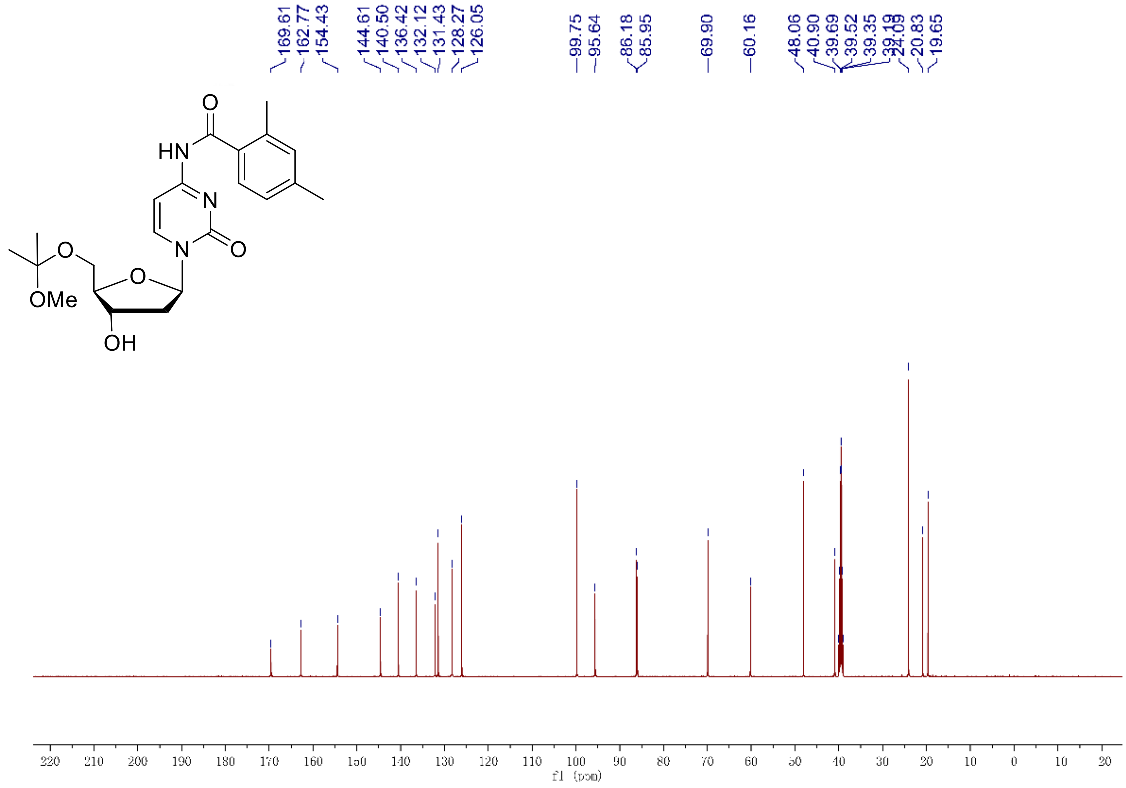

Figure S52. ^{13}C NMR (126 MHz, DMSO-d_6) spectrum of **10**. (DMSO solvent peaks: 39.7, 39.5, 39.4 and 39.2).

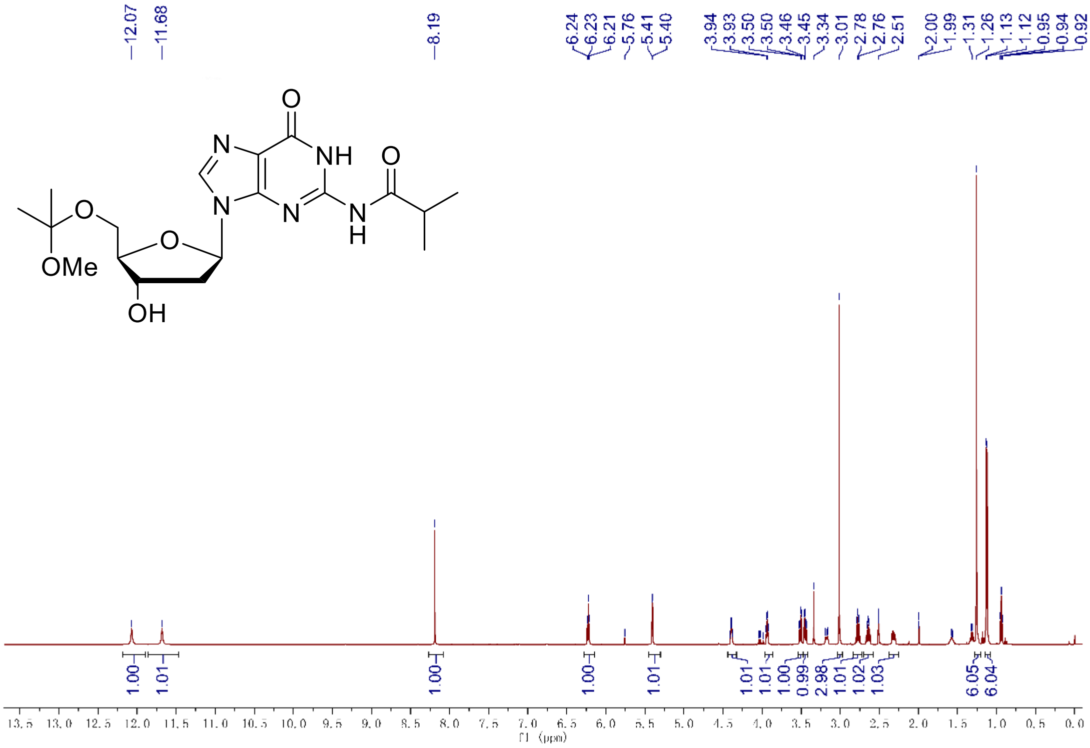

Figure S53. ^{1}H NMR (500 MHz, DMSO-d_6) spectrum of **11**.

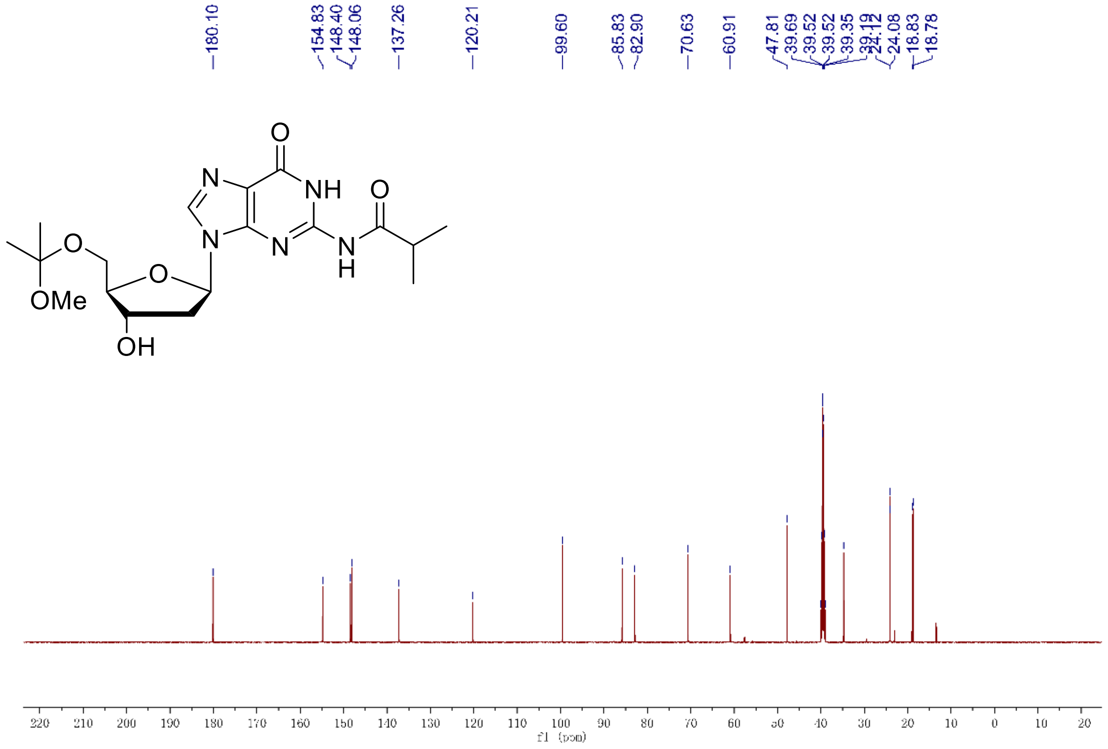

Figure S54. ^{13}C NMR (126 MHz, DMSO-d_6) spectrum of **11**. (DMSO solvent peaks: 39.7, 39.5, 39.4 and 39.2).

Scheme S1

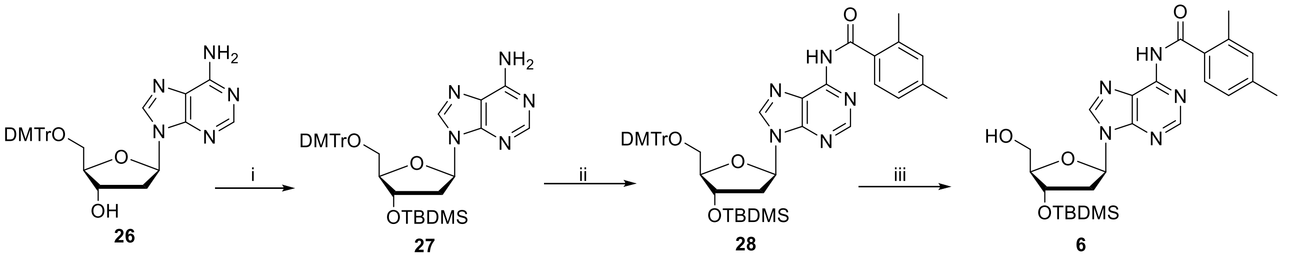

Conditions: i) Imidazole, TBDMSCl, DMF, 0 - 30°C, overnight, ii) 2,4-dimethylbenzoyl chloride, Et_3N, DCM, r.t., overnight, iii) $CH_3(CH_2)_{11}SH$, DCA, DCM, 0 – 5 °C, 1 h.

Synthesis of *N*^{6}-(2,4-dimethylbenzoyl)-3'-*O*-tert-butyldimethylsilyl-2'-deoxyadenosine (6)

26 (145 g, 261.9 mmol) was dissolved in 1.5 L anhydrous DMF, and cooled to 0-5 ℃. Imidazole (44.6 g, 655 mmol, 2.5 eq.) and TBDMSCl (59.3 g, 393 mmol, 1.5 eq.) were added and the mixture was stirred overnight at 30°C. 4.5 L ice water was added dropwise, and the mixture was stirred for 1 h. and filtered to give 160 g (240 mmol, 92 %) of **27** as off-white solid. MS(ESI): calculated for $C_{37}H_{45}N_5O_5Si^+$ 668.3268, found 668.3294 $[M+H]^+$.

27 (160.2 g, 240 mmol) was dissolved in 1.6 L anhydrous DCM and cooled to 0-5 ℃. After addition of TEA (170 g, 1680 mmol, 7.0 eq.) and 2,4-dimethylbenzoyl chloride (80.6 g, 480 mmol, 2.0 eq.), the solution was stirred at room temperature overnight. The reaction was quenched with $NaHCO_3$ solution, then partitioned, and the organic phase was dried and concentrated to give a brownish-yellow solid, consisting mainly of bis-acylated product (imide). The above solid was dissolved in 1.2 L ACN, 360 mL of ammonia was added, and stirred overnight at room temperature to cleave the imide intermediate to the desired amide. After the reaction, the ACN and excess ammonia were removed by evaporation at 30 ℃. The concentrate was dissolved in 1.2 L DCM, extracted with 1 L water, and the aqueous phase was washed twice with DCM (2x500 mL). The organic phase was dried over $MgSO_4$, filtered and concentrated to give the crude product. Purification by silica gel chromatography (Heptane/Ethyl acetate = 5:1~4:1, V/V) gave **28** (117.6 g, 146 mmol, 61% yield) as pale-yellow solid. ESI-MS (ES+) calculated for $C_{46}H_{53}N_5O_6Si^+$ 800.3843, found 800.3806 $(M+H)^+$.

28 (117.2 g, 146 mmol) and n-dodecanethiol (44.8 g, 221 mmol, 1.5 eq.) were dissolved in DCM (1.2 L). The mixture was cooled to 0-5 ℃ and dichloroacetic acid (28.6 g, 221 mmol, 1.5 eq.) was added dropwise. The mixture was stirred for 1 h, and quenched by addition aqueous $NaHCO_3$. The organic phase was separated, dried over Na_2SO_4 and concentrated to obtain a yellow oil. Purification by silica gel chromatography (Heptane/Ethyl acetate =3:1~1:3, V/V) gave 67.8 g (135 mol, 92 %) of **6** as white solid. ^{1}H NMR (500 MHz, DMSO-*d*6) δ 11.00 (s, 1H), 8.68 (d, *J* = 3.0 Hz, 2H), 7.47 (d, *J* = 7.8 Hz, 1H), 7.13 (s, 1H), 7.10 (d, *J* = 7.9 Hz, 1H), 6.46 (t, *J* = 6.8 Hz, 1H), 5.07 (t, *J* = 5.6 Hz, 1H), 4.64 (dt, *J* = 5.6, 2.9 Hz, 1H), 3.90 (dd, *J* = 7.4, 4.7 Hz, 1H), 3.63 (dt, *J* = 10.8, 5.3 Hz, 1H), 3.57 – 3.49 (m, 1H), 2.90 (ddd, *J* = 13.2, 7.2, 5.8 Hz, 1H), 2.42 (s, 3H), 2.38 – 2.34 (m, 1H), 2.33 (s, 3H), 0.91 (s, 9H), 0.13 (s, 6H). ^{13}C{1H} NMR (126 MHz, DMSO-d6) δ 167.8, 151.8, 151.6, 150.1, 143.1, 140.1, 136.7, 132.5, 131.5, 128.4, 126.1, 125.4, 88.1, 83.8, 72.5, 61.3, 40.0, 25.7(tBu), 20.9, 19.8, 17.7, -4.8, -4.9. MS(ESI): calculated for $C_{25}H_{36}N_5O_4Si^+$ 498.2537, found 498.2552 $[M+H]^+$.

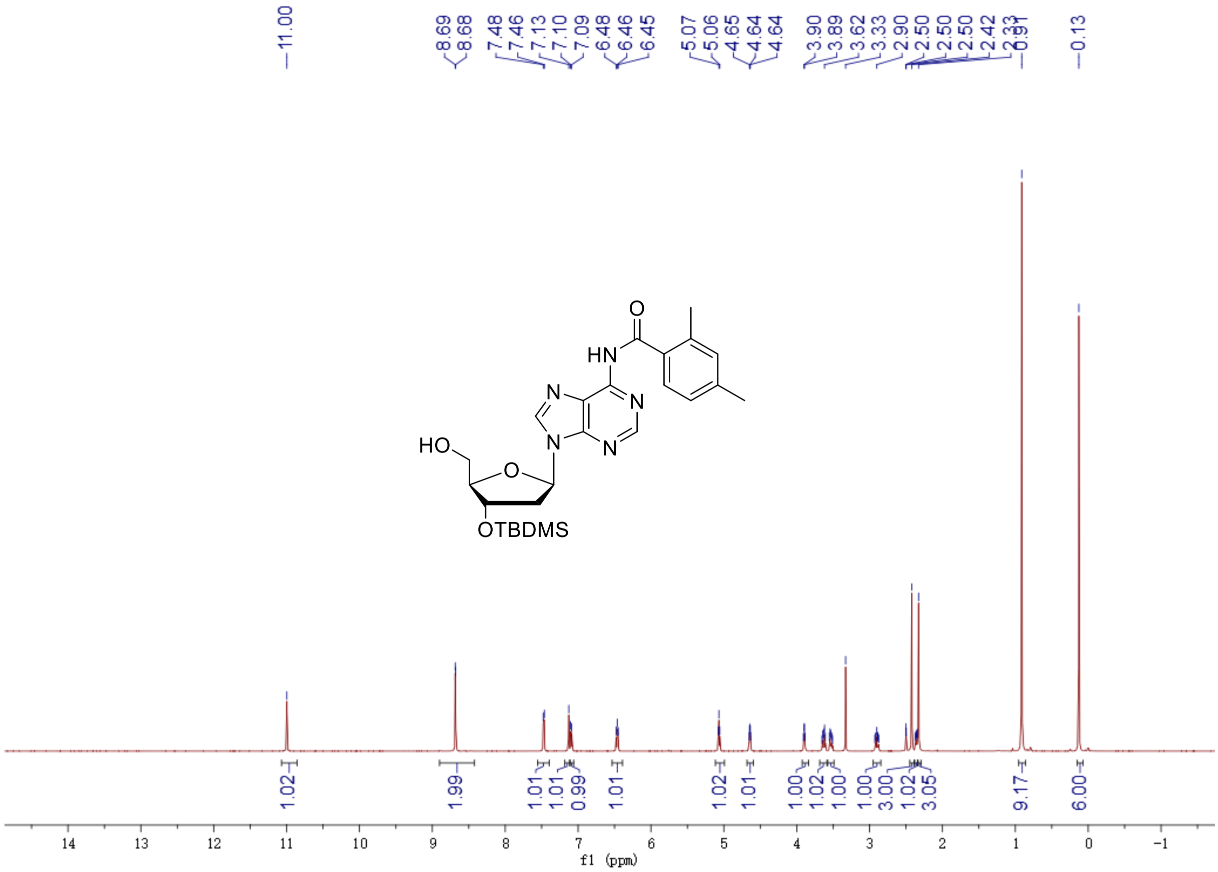

Figure S55. ^{1}H NMR (500 MHz, DMSO-d_6) spectrum of **6**.

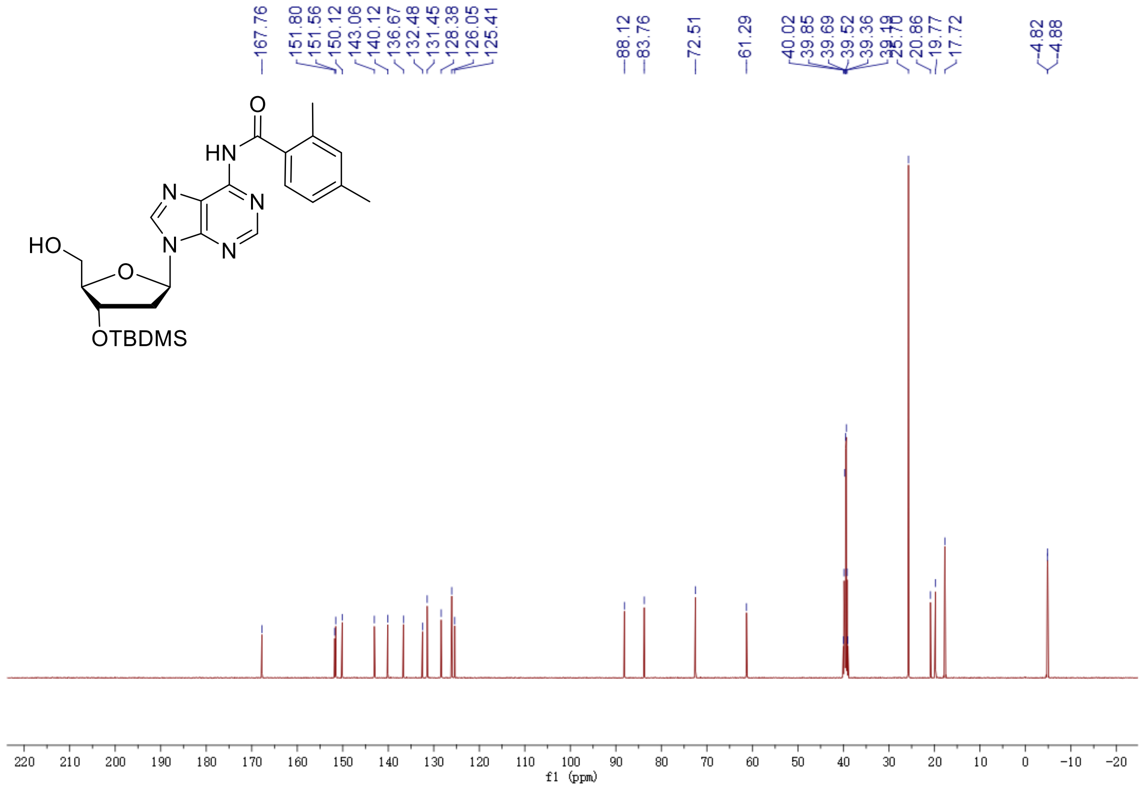

Figure S56. ^{13}C NMR (126 MHz, DMSO-d_6) spectrum of **6**. DMSO peaks 39.9, 39.7, 39.5, 39.4 and 39.2.

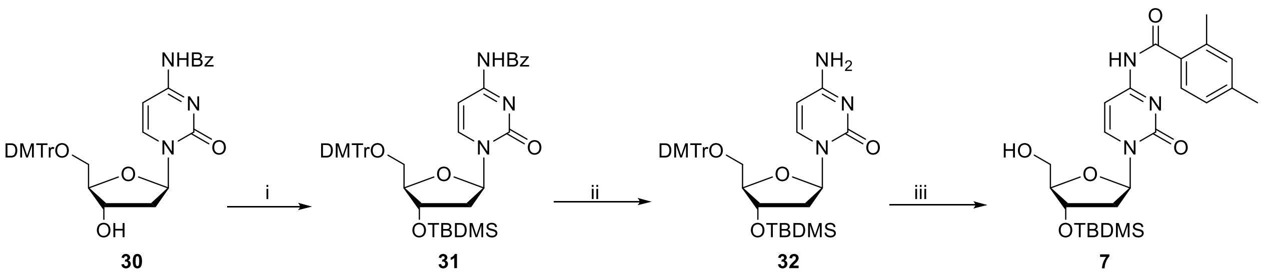

Conditions: i) Imidazole, TBDMSCl, DCM, r.t., 8h, ii) MeOH/NH3·H2O (1:1, *v/v*), 25 °C, 3 h, iii) 1) HOBt, EDCl, Et_3N, 2,4-dimethylbenzoic acid, DCM, 25 °C, 24 h, 2) $CH_3(CH_2)_{11}SH$, DCA, DCM, 25 °C, 2 h.

Synthesis of *N*^{4}-(2,4-dimethylbenzoyl)-3'-*O*-tert-butyldimethylsilyl-2'-deoxycytidine (7)

To a mixture of **30** (150.0 g, 236.7 mmol, 1.0 eq.) and imidazole (40.3 g, 591.8 mmol, 2.5 eq.) in DCM (1.5 L), TBDMSCl (71.4 g, 473.4 mmol, 2.0 eq.) was added in one portion. The reaction mixture was stirred at 25 °C for 8 hours. Then the reaction was quenched with $NaHCO_3$ (23.9 g, 284.0 mmol, 1.2 eq.), EtOH (13.1 g, 284.0 mmol, 1.2 eq.) and water (1.5 L), and stirred for 30 min. After quenching, the solution was washed with 10% NaCl aqueous solution twice. The organic phase was separated, dried over Na_2SO_4, filtered, and concentrated to give crude **31** as white solid (180 g, 236.7mmol) which was used for next step without any further purification. MS(ESI): calculated for $C_{43}H_{49}N_3O_7Si^+$ 748.3418, found 748.3453 (M+H$^+$).

31 (180 g crude, 236.7 mmol, 1.0 eq.) was dissolved in a mixed solvent of MeOH and NH3·H2O (1:1, *v/v*, 1.5 L) and stirred at 25 °C for 3 hours. Then the reaction mixture was concentrated under reduced pressure to dryness. After concentration, the residue was re-dissolved in DCM (1.5 L) and washed with 10 % NaCl aqueous solution twice. The organic phase was separated, dried over Na_2SO_4, filtered, and concentrated under reduced pressure to dryness to give crude **32** as white solid (170 g, 236.7 mmol) which was used for next step without any further purification. MS(ESI): calculated for $C_{36}H_{45}N_3O_6Si^+$ 644.3156, found 644.3127 (M+H$^+$).

Et_3N (125.9 g, 747.6 mmol, 5.0 eq.), HOBT (50.5 g, 373.8 mmol, 1.5 eq.), EDCI (71.6 g, 373.8 mmol, 1.5 eq.) and 2,4-dimethylbenzoic acid (45.0 g, 299.0 mmol, 1.2 eq.) were dissolved in DCM (800 mL). Then the mixture was added in a solution of **32** (170 g, 236.7 mmol, 1.0 eq.) in DCM (800 mL). After stirring at 25 °C for 24 hours, the reaction mixture was washed with 10 % citric acid aqueous solution for three times. The organic phase was separated, dried over Na_2SO_4 and filtered; the filtrate was used for next step directly. 1-Dodecanethiol (100.8 g, 498.4 mmol, 2.0 eq.) and dichloroacetic acid (45.8 g, 355.1 mmol, 1.5 eq.) were added to the above filtrate successively and the solution was stirred at 25 °C for 2 hours. Then the reaction was cooled to 0 – 10 °C and quenched with $NaHCO_3$ (47.1 g, 498.4 mmol, 2.0 eq.) and EtOH (21.8 g, 473.4 mmol, 2.0 eq.). The organic phase was separated and washed with 10 % NaCl aqueous solution, dried over Na_2SO_4, filtered and concentrated to give crude **7**. The crude product was purified by silica gel chromatography (EtOAc/DCM = 1:6~1:1, *v/v*) to give **7** as white solid (58 g, 122.5mmol, yield: 51.7%, over four steps). ^{1}H NMR (500 MHz, $CDCl_3$) δ 8.80 (s, 1H), 8.43 (d, *J* = 7.5 Hz, 1H), 7.60 (d, *J* = 7.4 Hz, 1H), 7.30 (s, 1H), 6.97 (s, 1H), 6.92 (d, *J* = 7.8 Hz, 1H), 6.12 (t, *J* = 6.0 Hz, 1H), 4.44 (dd, *J* = 10.4, 4.7 Hz, 1H), 4.01 – 3.92 (m, 2H), 3.89 (s, 1H), 3.74 (dd, *J* = 11.7, 2.3 Hz, 1H), 2.49 – 2.42 (m, 1H), 2.34 (s, 3H), 2.28 (s, 3H), 2.26 – 2.19 (m, 1H), 0.85 (s, 9H), 0.04 (s, 6H). ^{13}C{1H} NMR (126 MHz, $CDCl_3$) δ 169.0, 162.4, 155.5, 145.9, 141.9, 137.6, 132.5, 131.2, 127.3, 126.6, 96.6, 88.3, 88.1, 71.0, 61.3, 42.0, 25.8 (tBu), 21.4, 20.1, 18.0, -4.7, -4.9. MS(ESI): calculated for $C_{24}H_{36}N_3O_5Si^+$ 474.2424, found 474.2412 (M+H$^+$).

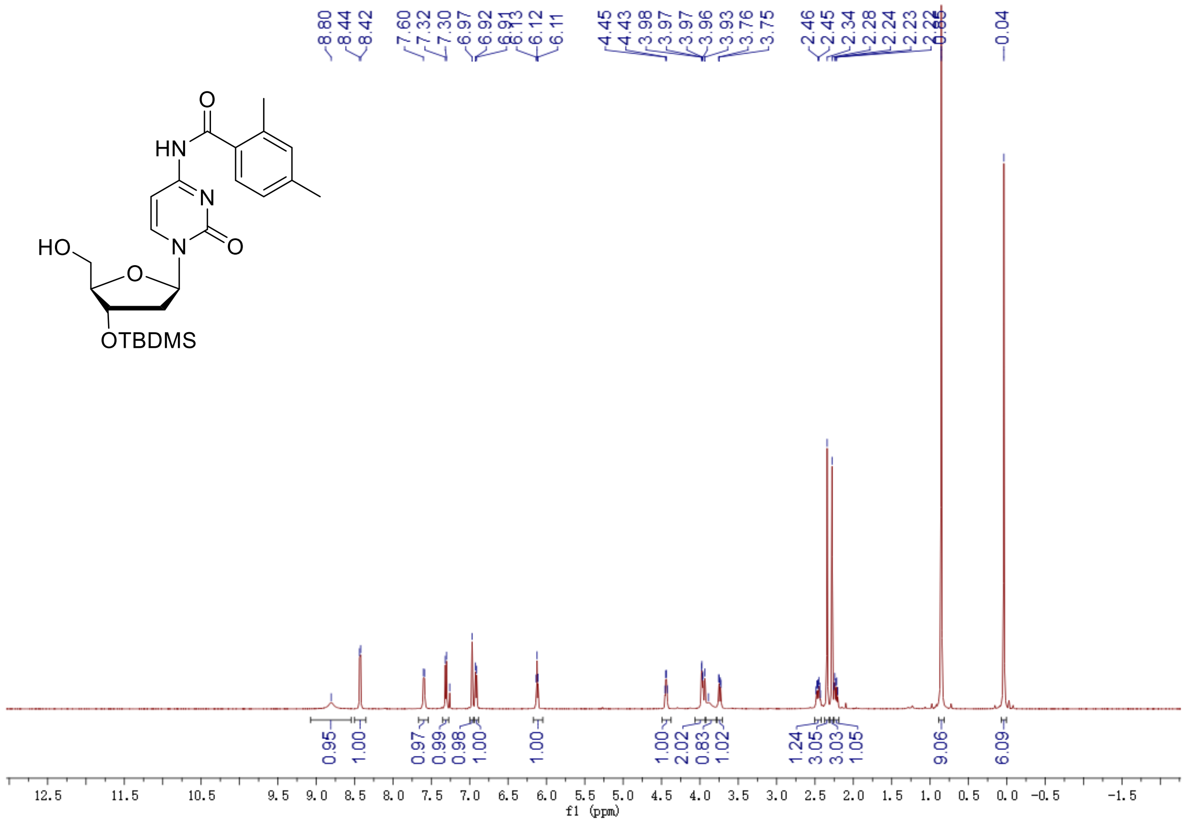

Figure S57. ^{1}H NMR (500 MHz, $CDCl_3$) spectrum of **7**.

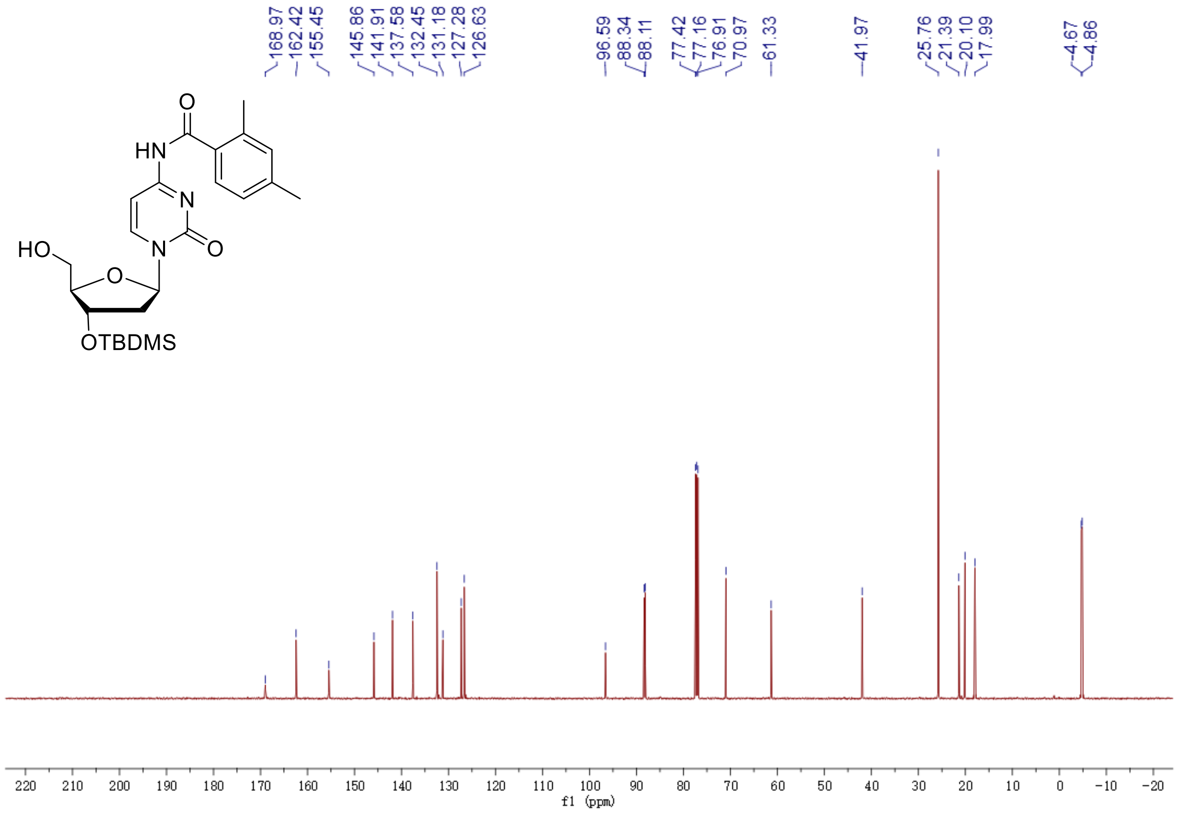

Figure S58. ^{13}C NMR (126 MHz, $CDCl_3$) spectrum of **7**. $CDCl_3$ peak 77.4, 77.2 and 76.9.

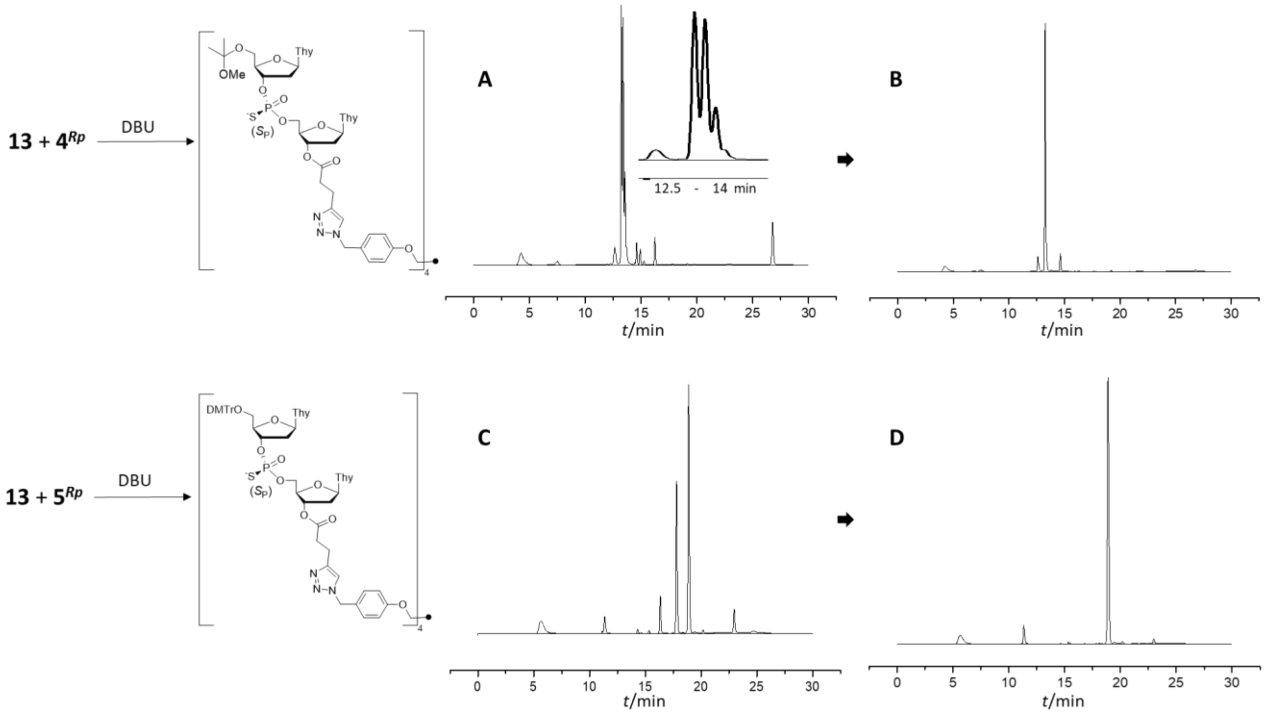

Figure S59. RP HPLC profiles of the coupling reactions with **4^{Rp}** and **5^{Rp}** on a soluble support **13**. The traces refer to aliquots withdrawn from the reactions solutions at tr = 1 min (A and C) and at tr = 30 min (B and D). Incomplete coupling can be seen as a set of tetra- tri- and divalent products in A and C.

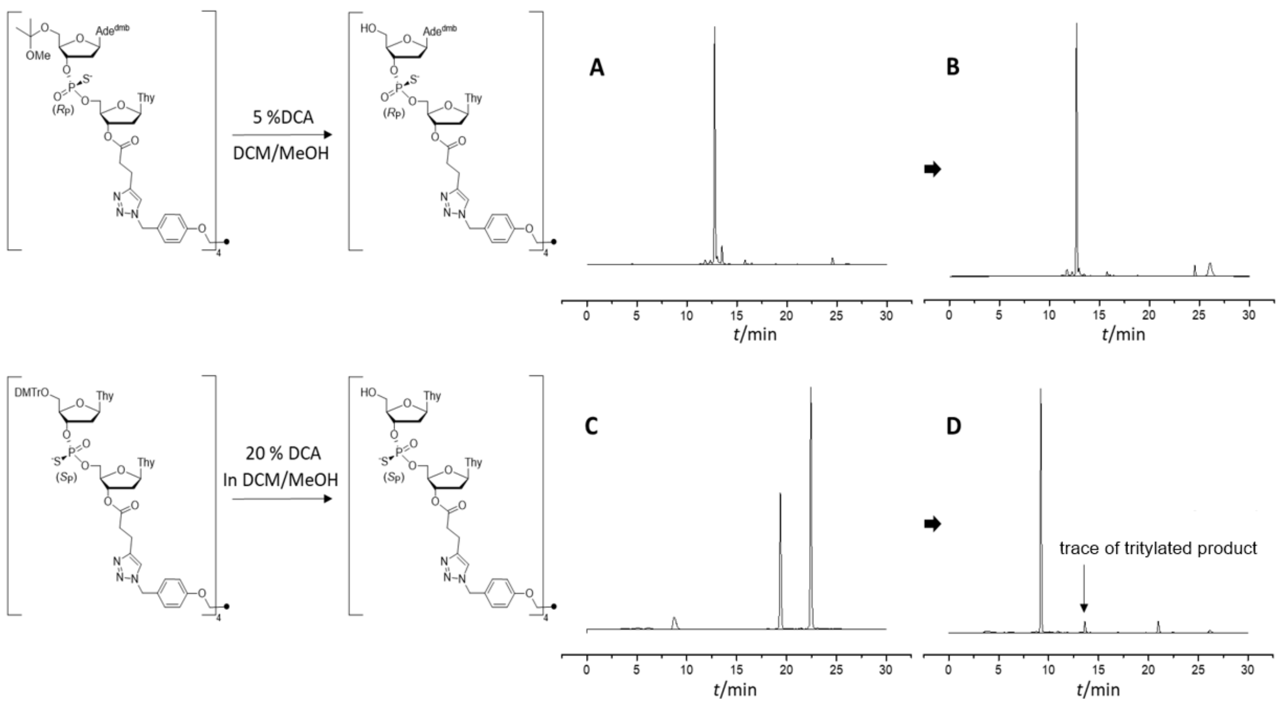

Figure S60. RP HPLC profiles of the 5´-deacetalization and 5´-detritylation reactions by 5 % or 20 % DCA in DCM/MeOH, respectively. The traces refer to aliquots withdrawn from the reaction solutions at (A) 2 min and (B) 4 min for the deacetalization, and (C) ~ 0 min and (D) 30 min for the detritylation.

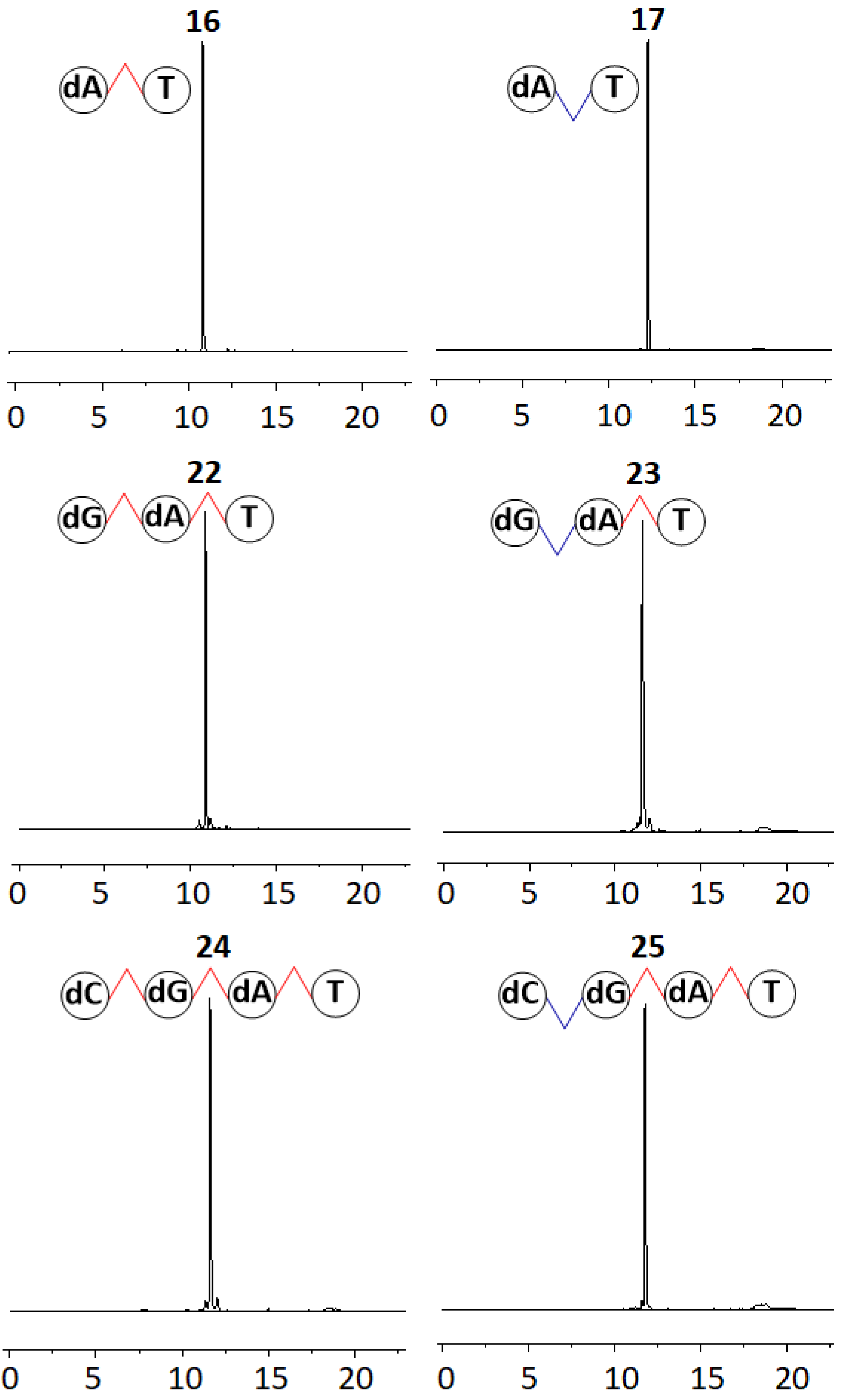

Figure S61. RP HPLC profiles of crude product (**16, 17, 22-25**) mixtures (cf. Figure 2 in the article).

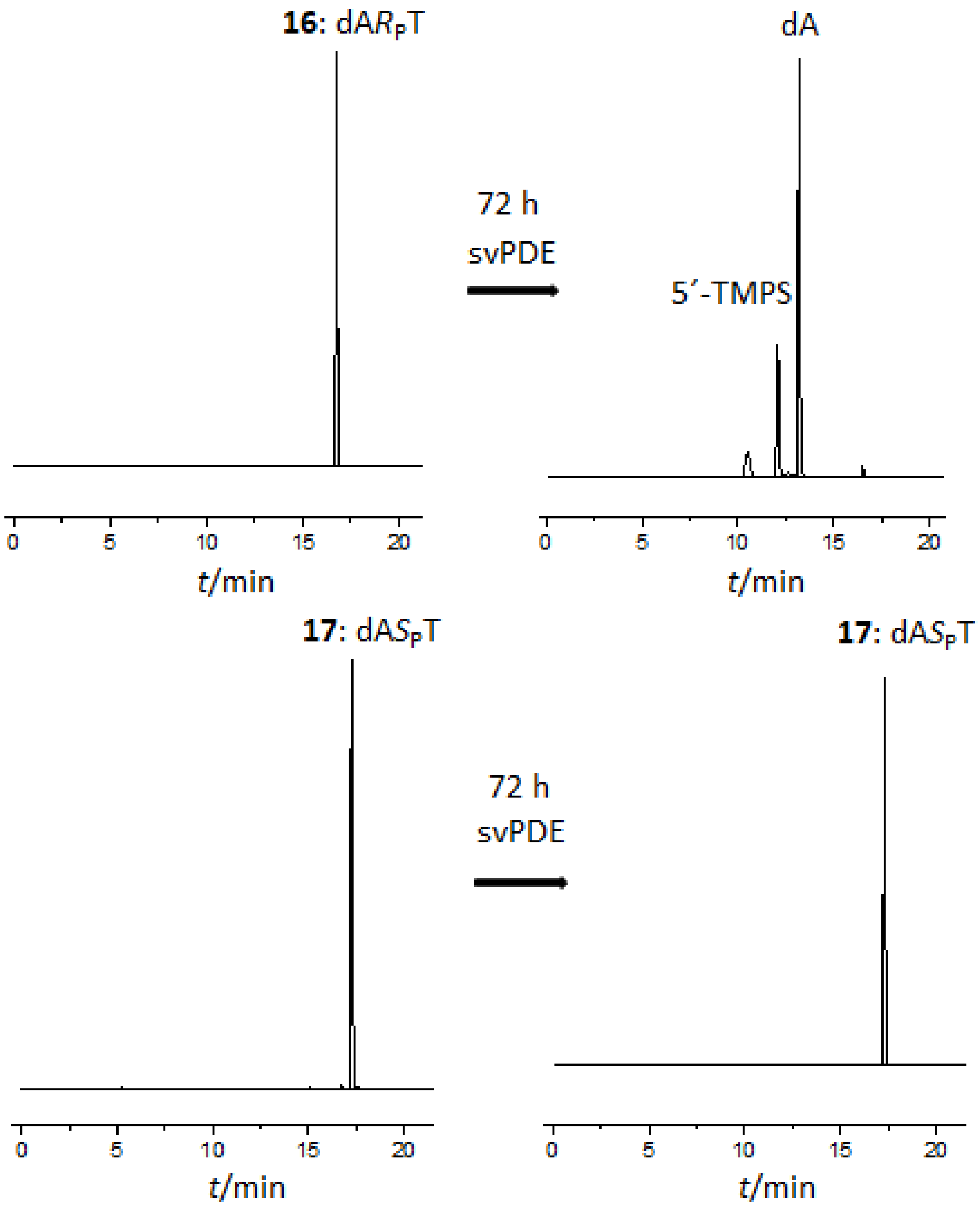

Figure S62. Product distribution for the enzyme-catalyzed hydrolysis of **16** and **17**. (snake venom phosphodiesterease at pH 8.5 and 37 °C ([$MgCl_2$] = 15 mM). The products 5´-TMPS ($[M+H]^+$ at *m/z* 339.0) and dA ($[M+H]^+$ at *m/z* 252.1) could be detected by MS analysis.

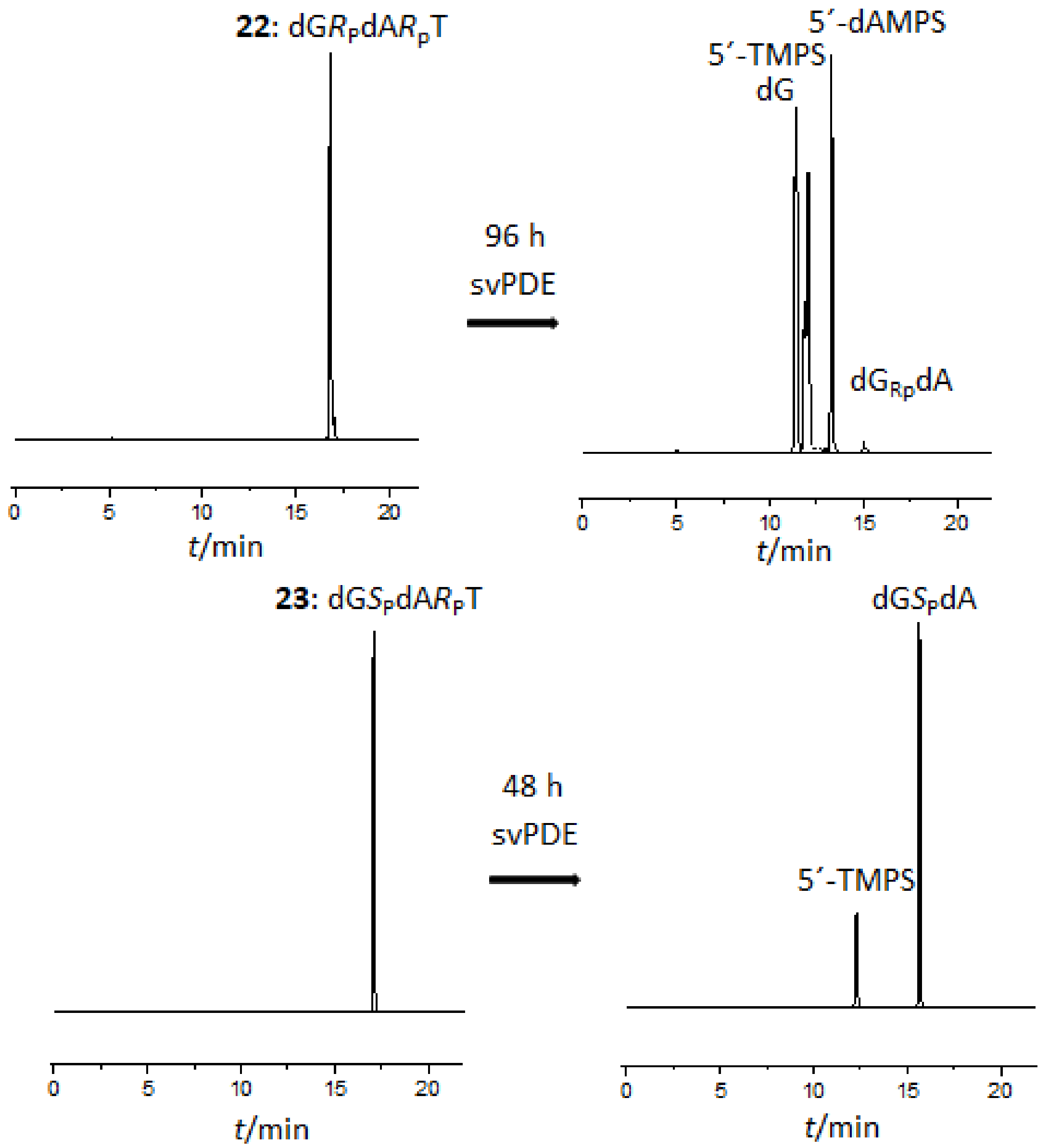

Figure S63. Product distribution for the enzyme-catalyzed hydrolysis of **22** and **23** (nake venom phosphodiesterease at pH 8.5 and 37 °C ([$MgCl_2$] = 15 mM). The products dG ([M-H]$^-$ at *m/z* 266.1 and [M+H]$^+$ at *m/z* 268.1), dG*R*PdA and dG*S*PdA ([M+H]$^+$ at *m/z* 597.2), 5´-dAMPS ([M-H]$^-$ at *m/z* 346.0 and [M+H]$^+$ at *m/z* 348.0) and 5´-TMPS ([M+H]$^+$ at *m/z* 339.0) could be detected by MS analysis.

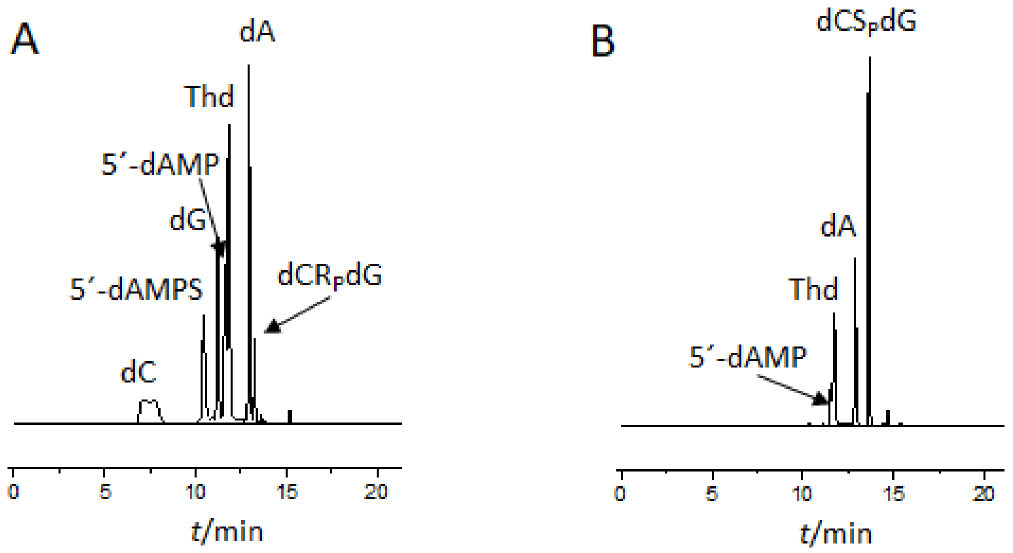

Figure S64. Product distribution for the enzyme-catalyzed hydrolysis of (A) **24** and (B) **25** (snake venom phosphodiesterease at pH 8.5 and 37 °C ($[MgCl_2]$ = 15 mM). The products dA ($[M+H]^+$ at *m/z* 252.1), Thd ($[M-H]^-$ at *m/z* 241.1), dG ($[M-H]^-$ at *m/z* 266.1 and $[M+H]^+$ at *m/z* 268.1), dC*R*PdG and dC*S*PdG ($[M+H]^+$ at *m/z* 573.1), dC ($[M+H]^+$ at *m/z* 228.1) and 5´-dAMPS or 5´-dGMP ($[M-H]^-$ at *m/z* 346.0 and $[M+H]^+$ at *m/z* 348.0) could be detected by MS analysis. Prolonged treatment with Tris-HCL buffer resulted in slow desulfurization of thiophosphodiester bond. A small amount of 5´-dAMP ($[M-H]^-$ at *m/z* 330.1 and $[M+H]^+$ at *m/z* 332.1) was observed as a product.
